# Chiral ^1^H NMR of Atropisomeric Quinazolinones With Enantiopure Phosphoric Acids

**DOI:** 10.3389/fchem.2018.00300

**Published:** 2018-08-17

**Authors:** Chaofei Wu, Hongxin Liu, Juan Li, Hong-Ping Xiao, Xinhua Li, Jun Jiang

**Affiliations:** College of Chemistry and Materials Engineering, Wenzhou University, Wenzhou, China

**Keywords:** chiral recognition, ^1^H NMR analysis, quinazolinones, chiral phosphoric acid, high accuracy

## Abstract

A chiral phosphoric acid promoted enantioselective NMR analysis of atropisomeric quinazolinones was described, in which a variety of racemic arylquinazolinones such as afloqualone and IC-87114 were well recognized with up to 0. 21 ppm ΔΔδ value. With this method, the optical purities of different non-racemic substrates can be fast evaluated with high accuracy.

After the first experimental detection of atropisomerism by Christie and Kenner in 1922 (Christie and Kenner, 1922), axial chirality was gradually recognized as an important type of molecular asymmetry which derived from the restricted rotation of a single bond in biaryls, amines, etc. For example, axially chiral BINAP and its analogs were found to be excellent ligands in various asymmetric catalytic transformations (Miyashita et al., 1980; Akutagawa, 1995; Kumobayashi et al., 2001; Brunel, 2005, 2007; Genet et al., 2014), while a lot of optically active biaryl natural products were successfully isolated and identified in the past few decades (Bringmann et al., 2011; Smyth et al., 2015). Besides, atropisomers were found to exhibit different pharmacodynamics and pharmacokinetics in many cases (Eichelbaum and Gross, 1996; Clayden et al., 2009). Thus, exploring efficient chiral recognition and determination method for atropisomeric compounds is crucial to the asymmetric synthesis as well as structure-bioactivity study. With the fast development of analysis technology, GC (Schurig and Nowotny, 1990), IR (Reetz et al., 1998), HPLC (Han, 1997), circular dichroism (Ding et al., 1999; Nieto et al., 2008, 2010; Ghosn and Wolf, 2009), fluorescence spectroscopy (James et al., 1995; Mei and Wolf, 2004; Pu, 2004; Zhao et al., 2004; Li et al., 2005; Tumambac and Wolf, 2005; Liu et al., 2009), electrophoresis technologies (Reetz et al., 2000) and NMR spectroscopy have been frequently employed in chiral determinations. Among these methods, we are particularly interested in the NMR based chiral analysis method which employs chiral shift reagents (CSRs) (Frazer et al., 1971; Goering et al., 1971; Yeh et al., 1986; Ghosh et al., 2004; Yang et al., 2005) or chiral solvating reagents (CSAs) (Pirkle, 1966; Lancelot et al., 1969; Parker, 1991; Wenzel and Wilcox, 2003; Seco et al., 2004; Lovely and Wenzel, 2006; Ema et al., 2007; Wenzel, 2007; Iwaniuk and Wolf, 2010; Moon et al., 2010; Gualandi et al., 2011; Pham and Wenzel, 2011; Quinn et al., 2011; Wenzel and Chisholm, 2011; Ma et al., 2012; Labuta et al., 2013; Zhou et al., 2015; Akdeniz et al., 2016; Bian et al., 2016a,b; Huang et al., 2016) to directly differentiate enantiomers of the analytes, since it takes many advantages such as easy operation, fast evaluation, broad analyte scope and so on. In 2017, we reported a chiral phosphoric acid (CPA) promoted enantioselective NMR analysis of indoloquinazoline alkaloid type tertiary alcohols with high efficiency and wide application; this methodology was also employed in the fast optimization of reaction conditions in amino acid metal salt catalyzed asymmetric aldol reaction via direct analysis of the reaction mixture without purification (Liu et al., 2017). Inspired by this result and given the growing interest in axially chiral compounds, atropisomeric arylquinazolinones, which are constituents of various biologically active natural products and pharmaceutical compounds, were chosen as the next target to further evaluate the chiral recognition ability of chiral phosphoric acids (Figure 1). Herein, we wish to report our preliminary results on this topic: in the presence of 0.2–1.5 equiv. of α-naphthyl phosphoric acid, a variety of racemic arylquinazolinones including afloqualone and IC-87114 were well recognized with up to 0.21 ppm ΔΔδ value; additionally, the corresponding analysis system can also be employed in the accurate determination of enantioselectivities of chiral arylquinazolinones.

**Figure 1:**
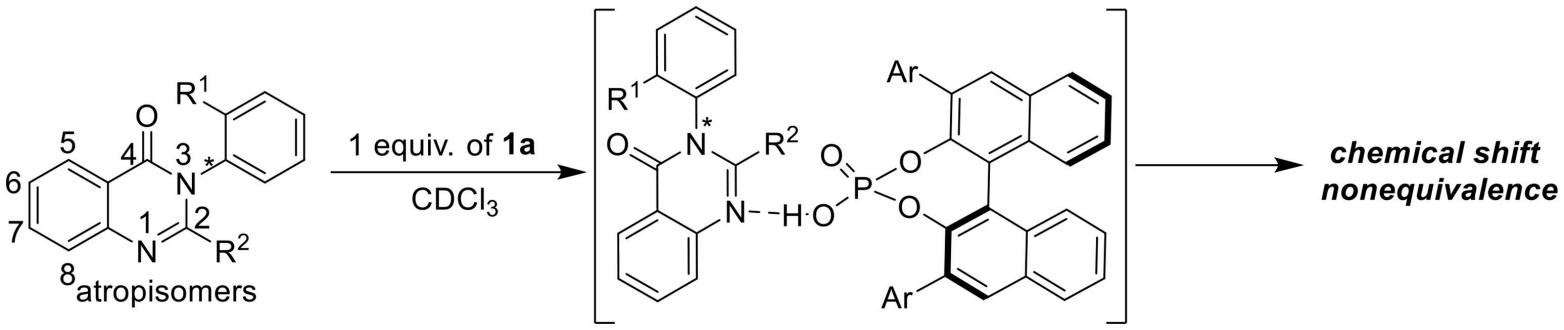
Chiral ^1^H NMR analysis of arylquinazolinones with a chiral phosphoric acid.

Our exploration of this hypothesis began with evaluating the interaction between chiral phosphoric acids 1 and racemic 2-(furan-2-yl)-3-(o-tolyl)quinazolin-4(3H)-one 2a in CDCl_3_ at 25°C. As shown in Table 1, after additions of 1 equiv. of chiral phosphoric acids to racemic 2a, chemical shift non-equivalences were observed. However, the structure of phosphoric acids has obvious influence on the recognition result. For example, BINOL derived phosphoric acid 1a was found to be the best host which afforded a baseline resolution and the largest chemical shift nonequivalence of methyl H signal of 2a (0.16 ppm), while spiro-phosphoric acid 1i with the same substituents exhibited poor chiral recognition ability; additionally, phosphoric acids with very bulky substituents such as 1b and 1j also failed to provide satisfactory discriminating results (For details, see Supplementary Materials). With optimal host 1a in hand, the effect of deuterated solvents was also studied. It was shown that the interaction between chiral phosphoric acid 1a and guest 2a existed even in protic and polar solvents (CD_3_OD, acetone-D_6_, DMSO-D_6_); however, CDCl_3_ was still found to be the best choice of solvent. To further explore the chiral recognition ability of 1a, attempts to evaluate the influence of the amount of 1a were also carried out. Generally, larger amount of 1a led to better recognition results (entries 1, 16–18). Noticeably, it was found that 20 mol% 1a was enough to give clear baseline resolution of 2a under standard analysis condition, albeit with smaller chemical shift nonequivalence (entry 16, 0.05 ppm). Finally, as the optimal compromise between discriminating result and atom economy, 1 equiv. of chiral phosphoric acid 1a was chosen in standard analysis conditions.

**Table 1:**
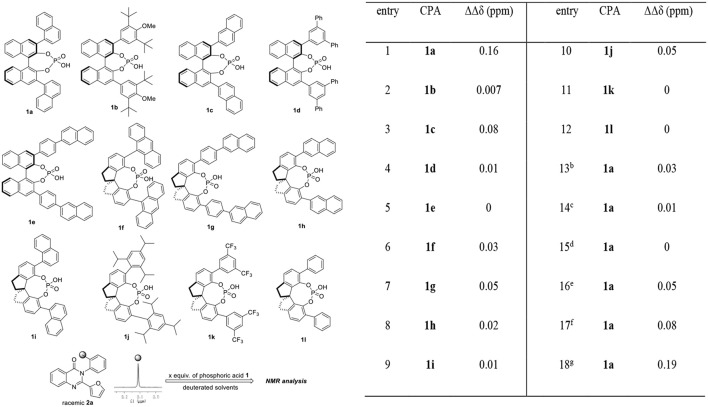
Evaluating the chiral recognition abilities of chiral phosphoric acids **1** with **2a**.

^a^* All samples were prepared by mixing 1 (0.01 mmol) and the guests 2a (0.01 mmol) in CDCl_3_ at 25°C*.

^b^*Acetone-D_6_ was used as deuterated solvent*.

^c^*CD_3_OD was used as deuterated solvent*.

^d^*DMSO-D_6_ was used as deuterated solvent*.

^e^*0.2 equiv. of 1a was used*.

^f^*0.5 equiv. of 1a was used*.

^g^*1.5 equiv. of 1a was used*.

The general applicability of these conditions for a variety of racemic 3-arylquinazolinones was fully demonstrated in Table 2. In the presence of 1 equiv. of phosphoric acid **1a** in 0.5 mL CDCl_3_ at 25°C, a number of racemic 3-arylquinazolinones derivatives **2b**−**2m** with various substituents can be well resolved. For example, other aromatic moieties such as thiophene or quinoline on 2 position of 3-arylquinazolinones can also result in good enantiodiscrimination, albeit with smaller ΔΔδ value (**2b** and **2c**, 0.02–0.06 ppm); besides, 2-methyl substituted 3-arylquinazolinones with either electron-withdrawing group or electron-donating group on 6, 7, 8 position were proved to be good guests under optimal conditions, affording baseline resolutions with good chemical shift nonequivalence (**2d**−**2k**, 0.02-0.21 ppm). Noticeably, 3-(2-methoxyphenyl)-2-methyl-6-nitroquinazolin-4(3H)-one **2e** was well recognized with the largest ΔΔδ value of 0.21 ppm. Additionally, different substituents such as methyl, methoxyl or halogen group on the N-3 benzene ring were also well tolerated. However, when 2′-iodo-benzene was used as substituents on N-3 position, 1.5 equiv of **1a** was found necessary to afford satisfactory result (entry 12).

**Table 2:**
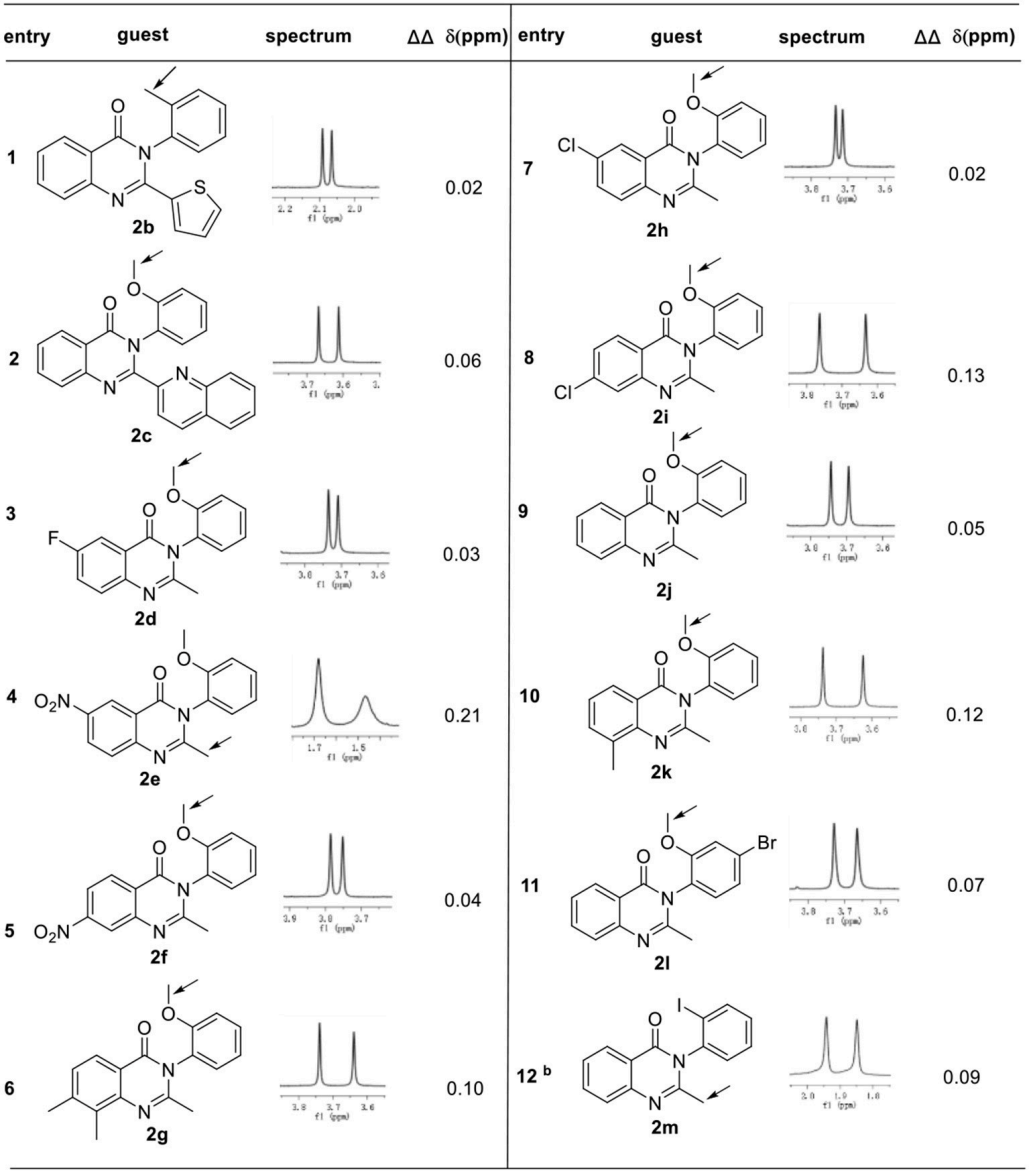
Differentiating the enantiomers of different racemic 3-arylquinazo-linones derivatives **2** in the presence of phosphoric acid **1a**^a^.

^a^*All samples were prepared by mixing **1a** (0.01 mmol) and the guests **2** (0.01 mmol) in CDCl_3_ at 25°C*.

*^b^ 0.015 mmol **1a** was used*.

To demonstrate the practical utility of our methodology, commercial drugs (candidate) were next selected for chiral recognition with phosphoric acid **1a**. To our delight, racemic 3-arylquinazolinone type bioactive molecules such as multifunctional afloqualone and IC-87114 can be well enantiodiscriminated with baseline resolutions under standard analysis conditions (Figure 2), for details, see Supplementary Materials.

**Figure 2:**
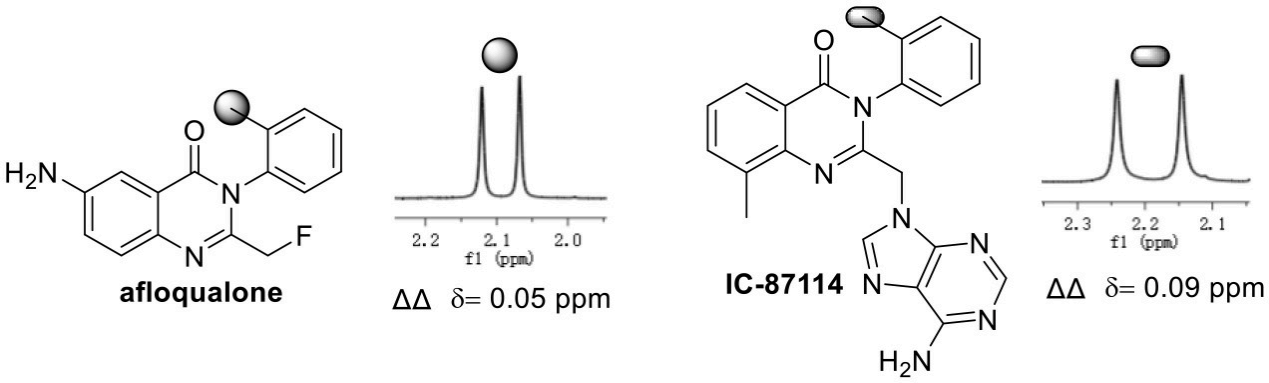
afloqualone and IC-87114 were discriminated under standard conditions.

Encouraged by these good discrimination results, this methodology was subsequently applied to the enantiomeric determination of various non-racemic **2a** samples. As shown in Figure 3, the optical purities of **2a** can be accurately obtained by integrating the corresponding H signals of methyl group of **2a** in the presence of 1 equiv. of **1a**. Compared with those data obtained from chiral HPLC analysis, excellent linear relationship and up to 1.4% absolute errors were obtained.

**Figure 3:**
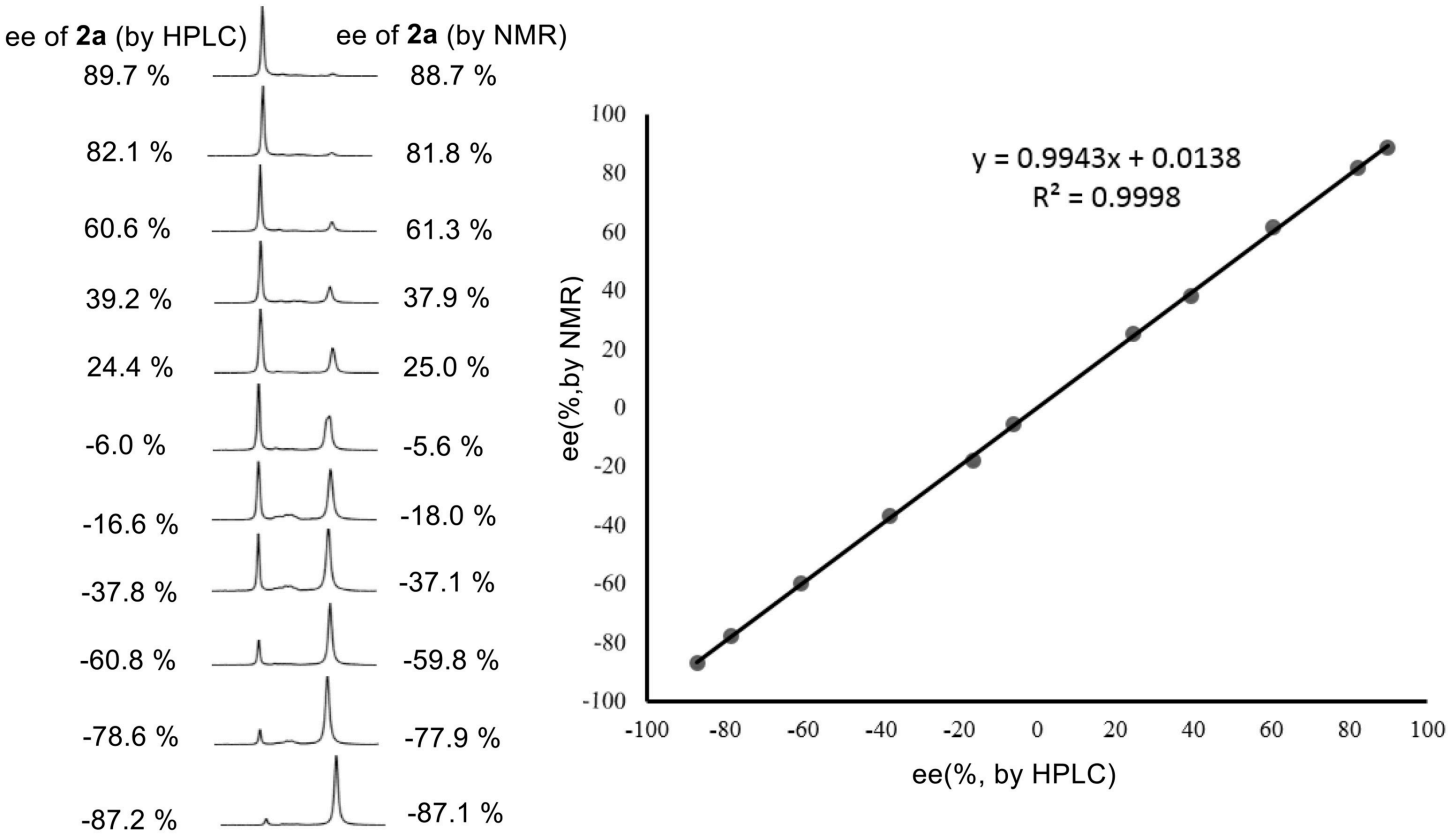
^1^H NMR signals of non-racemic **2a** samples in the presence of 1 equiv. of **1a** in CDCl_3_ (**left**); linear relationship between NMR measured ee values versus the HPLC determined ee values (**right**).

## Conclusions

In conclusion, we developed an efficient chiral NMR analysis method for atropisomeric quinazolinones, in which chiral phosphoric acid shows excellent abilities to discriminate the enantiomers of various 3-arylquinazolinones with good chemical shift non-equivalence. With this method, the optical purities of different non-racemic **2a** can be fast evaluated with high accuracy. Further studies on the interactions of chiral phosphoric acid with other analytes are currently underway.

## Author contributions

JJ and HL designed the project, guided the study, and polished the manuscript. CW and JL: conducted the experiments and characterized the samples. H-PX and XL revised the manuscript.

### Conflict of interest statement

The authors declare that the research was conducted in the absence of any commercial or financial relationships that could be construed as a potential conflict of interest.
