# Supplementary material for: Chiral 1H NMR of Atropisomeric Quinazolinones With Enantiopure Phosphoric Acids
Source: Front Chem. 2018 Aug 17;6:300. doi: 10.3389/fchem.2018.00300 (PMC6107746; doi:10.3389/fchem.2018.00300)
Supplement: Supplementary file 1 [file Presentation_1.PDF]

Supplementary Material

Chiral 1H NMR of Atropisomeric Quinazolinones with Enantiopure Phosphoric Acids

Chaofei Wu, Hongxin Liu,* Juan Li, Hong-Ping Xiao, Xinhua Li and Jun Jiang*

College of Chemistry and Materials Science, Wenzhou University, Wenzhou 325035, PR China.

E-mail: junjiang@wzu.edu.cn; hongxin-107@163.com

Contents

1. General information 2

2. General procedure for the synthesis of substrates **2** 3

3. Procedure for the chiral analysis of Atropisomeric Quinazolinone. 4

4. Evaluating the chiral recognition abilities of chiral sensors **1** with **2a** 5

5. ^{1}H NMR spectra of chiral sensors **1** and racemic **2a**. 6

6. ^{1}H NMR spectra of **1a** and racemic **2**. 15

7. ^{1}H NMR spectra/HPLC data of **1a** and **2** with different optical purities 33

1 1. General information

All commercial reagents were used as received without further purification unless otherwise stated. ^{1}H (NMR 500 MHz) spectra were recorded in DMSO-D_6, CD_3OD, Acetone-D_6 or $CDCl_3$ solutions using a Bruker 500MHz spectrometer at 25 ºC. Chemical shifts were reported in parts per million (ppm, δ) relative to residual TMS. Chiral HPLC analyses were performed on a Shimdzu LC-20A liquid chromatography. **2a**-**2m** were synthesized following the modified procedure of literature; the chiral phosphoric acids (**1a** and **1c**) were synthesized by known procedures.

2. General procedure for the synthesis of substrates 2:

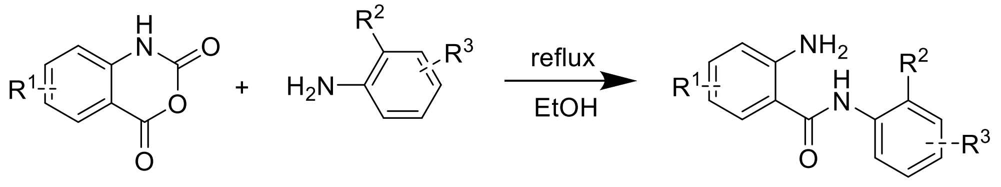

A **B** **C**

To a mixture of isatoic anhydride **A** (33 mmol; 1.0 equiv.), and amine **B** (33 mmol; 1.0 equiv.) in anhydrous ethanol (40 mL) and the mixture was stirred and refluxed at 90° C for 20 hours. After the reaction was completed, the reaction was stopped and solvent was removed, 0.06 N aqueous HCl (160 mL) was added and stirred vigorously. The suspended solid was filtered, Na_2CO_3 was added to adjust the pH=9, filtered again, the solid of two time was dried. The crude product was further purified by column chromatography to give the product **C** (75%-87% yield).

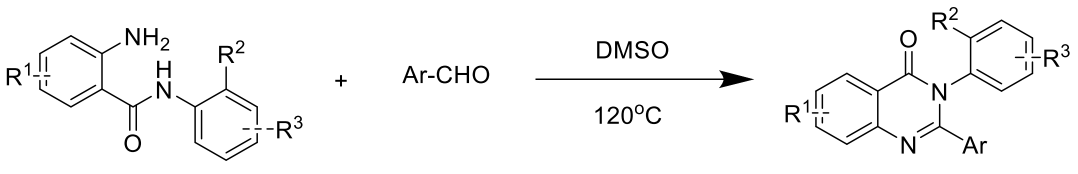

C **D** **2**

An N-substituted anthranilamide **C** (1.0 mmol; 1.0 equiv.) and an aldehyde **D** (1.2 mmol; 1.2 equiv.) were dissolved in DMSO (5 mL). Then, the reaction mixture was stirred at 120 °C in an open flask and monitored by TLC. After complete consumption of the starting materials, the reaction mixture was poured onto water and extracted with methylene chloride. The organic layer was combined, dried over anhydrous magnesium sulfate, and concentrated in vacuo. The crude mixture was purified by column chromatography on silica using hexanes/ethyl acetate (5:1) as an eluent to afford 2,3-disubstituted-4(3H)-quinazolinone **2** (65%-73% yield).

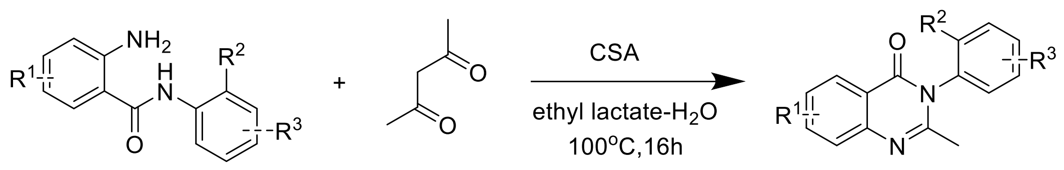

C **E** **2**

A flask was charged with the N-substituted anthranilamide **C** (0.2 mmol; 1.0 equiv.), pentane-2,3-dione **E** (0.3 mmol; 1.5 equiv.), CSA (0.02 mmol; 10% equiv.), and 1 : 9 (v/v) ethyl lactate-H_2O (1.0 mL).The

flask was sealed and the mixture was stirred at 100 ºC for 16 h. When the reaction was complete (TLC), the mixture was cooled to r.t., extracted with EtOAc, and washed with H_2O. The organic phase was dried (Na_2SO_4), filtered, and concentrated under reduced pressure. The residue was purified by column chromatography (silica gel) to give the product **2** (67%-93% yield).

3. Procedure for the chiral analysis of Atropisomeric Quinazolinone

Phosphoric acid **1a** (0.01 mmol) and the guests **2** (0.01 mmol) were mixed in $CDCl_3$ (0.6 mL), and ^1H NMR data were collected on a 500 MHz spectrometer at 25 °C. Chiral HPLC analyses were performed on a Shimdzu LC-20A liquid chromatography (Daicel Chiralpak AD-H, hexane/iso-propanol=90/10, flow rate 1.0mL/min).

4. Evaluating the chiral recognition abilities of chiral sensors 1a-1l (0.01 mmol) with 2a (0.01 mmol) in $CDCl_3$ at 25 ºC.

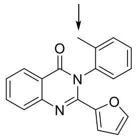
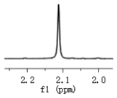

racemic 2a

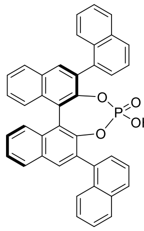

1a

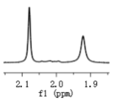

racemic **2a** with CPA **1a**
ΔΔδ= 0.16 ppm

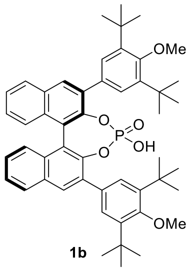

1b

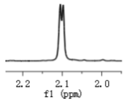

racemic **2a** with CPA **1b**
ΔΔδ= 0.007 ppm

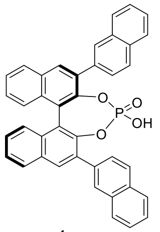

1c

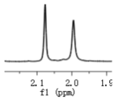

racemic **2a** with CPA **1c**
ΔΔδ= 0.08 ppm

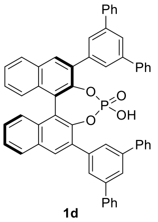

1d

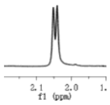

racemic **2a** with CPA **1d**
ΔΔδ= 0.01 ppm

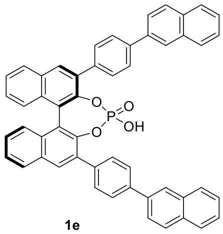

1e

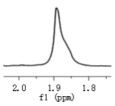

racemic **2a** with CPA **1e**
ΔΔδ= 0 ppm

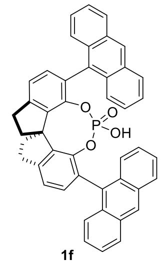

1f

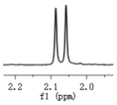

racemic **2a** with CPA **1f**
ΔΔδ= 0.03 ppm

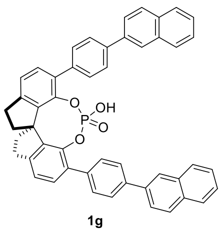

1g

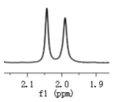

racemic **2a** with CPA **1g**
ΔΔδ= 0.05 ppm

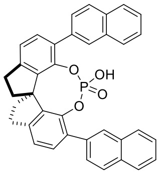

1h

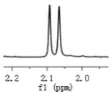

racemic **2a** with CPA **1h**
ΔΔδ= 0.02 ppm

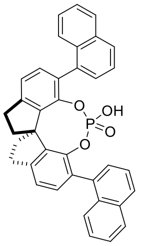

1i

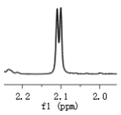

racemic **2a** with CPA **1i**
ΔΔδ= 0.01 ppm

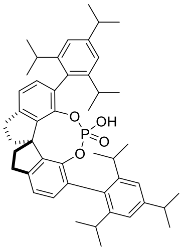

1j

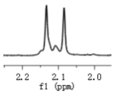

racemic **2a** with CPA **1j**
ΔΔδ= 0.05 ppm

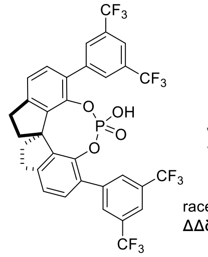

1k

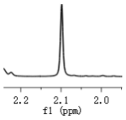

racemic **2a** with CPA **1k**
ΔΔδ= 0 ppm

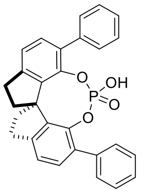

1l

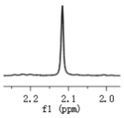

racemic **2a** with CPA **1l**
ΔΔδ= 0 ppm

5. ^{1}H NMR spectra of chiral sensors 1a-1f (0.01 mmol, 1.0 equiv.) and racemic 2a (0.01 mmol, 1.0 equiv.).

5.1. ^{1}H NMR (500 MHz, $CDCl_3$) of racemic 2a.

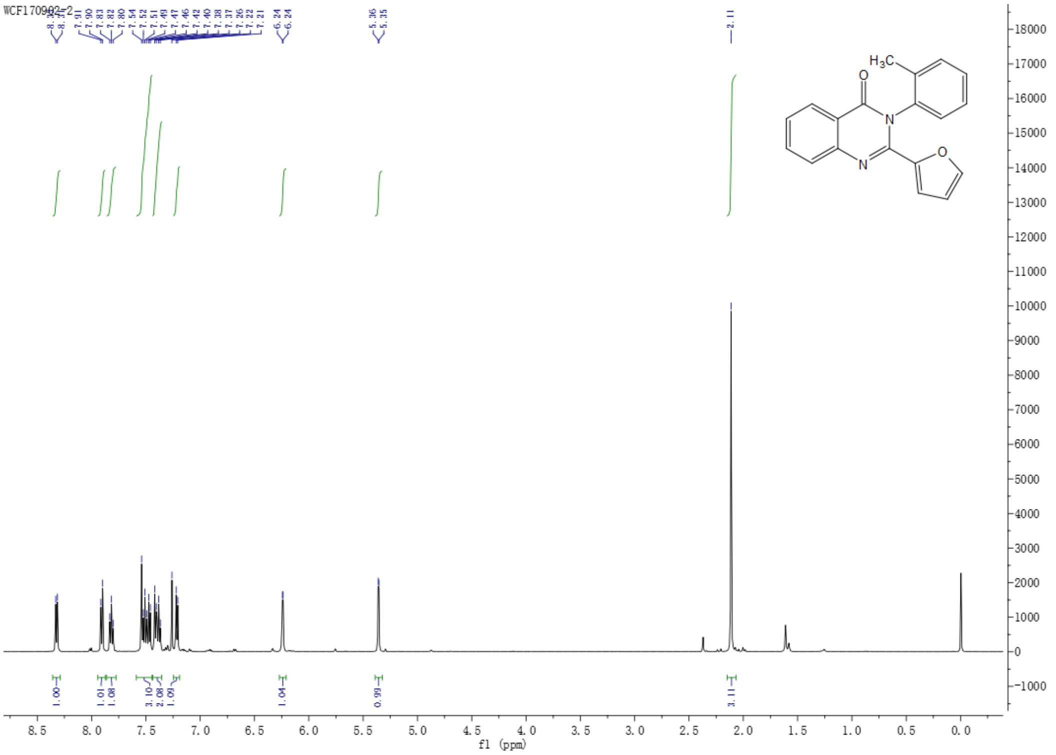

5.2. ^{1}H NMR (500 MHz, $CDCl_3$) of CPA 1a and guest 2a.

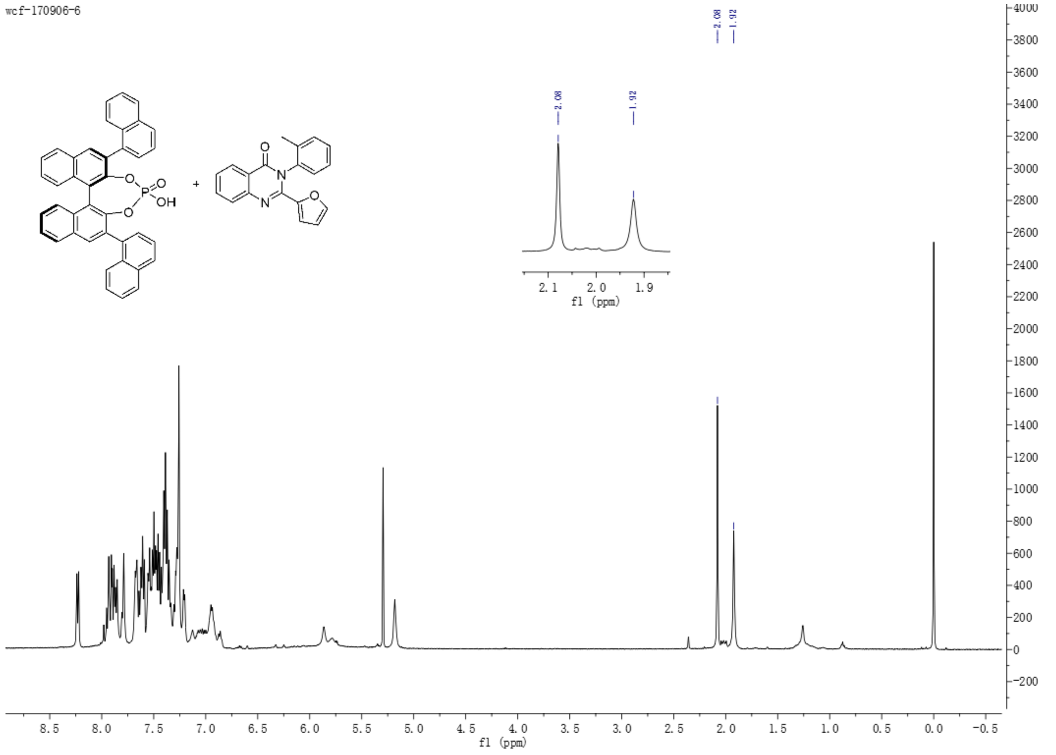

5.3. ^{1}H NMR (500 MHz, $CDCl_3$) of CPA 1b and guest 2a.

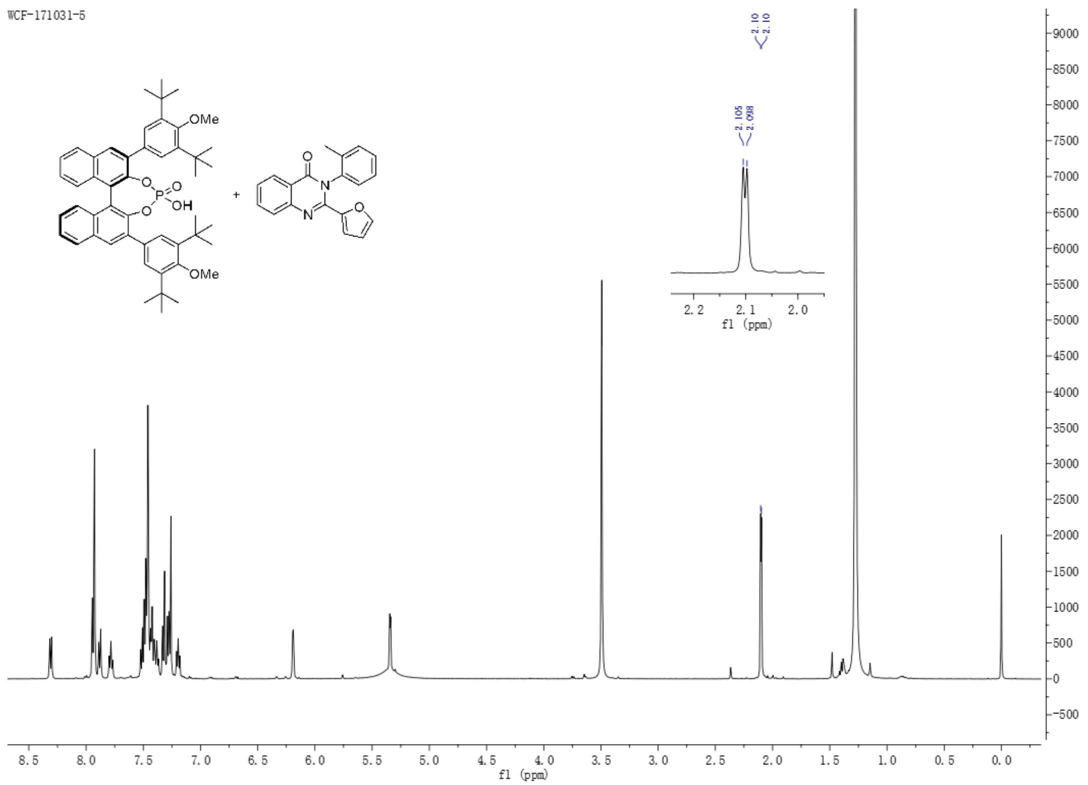

5.4. ^{1}H NMR (500 MHz, $CDCl_3$) of CPA 1c and guest 2a.

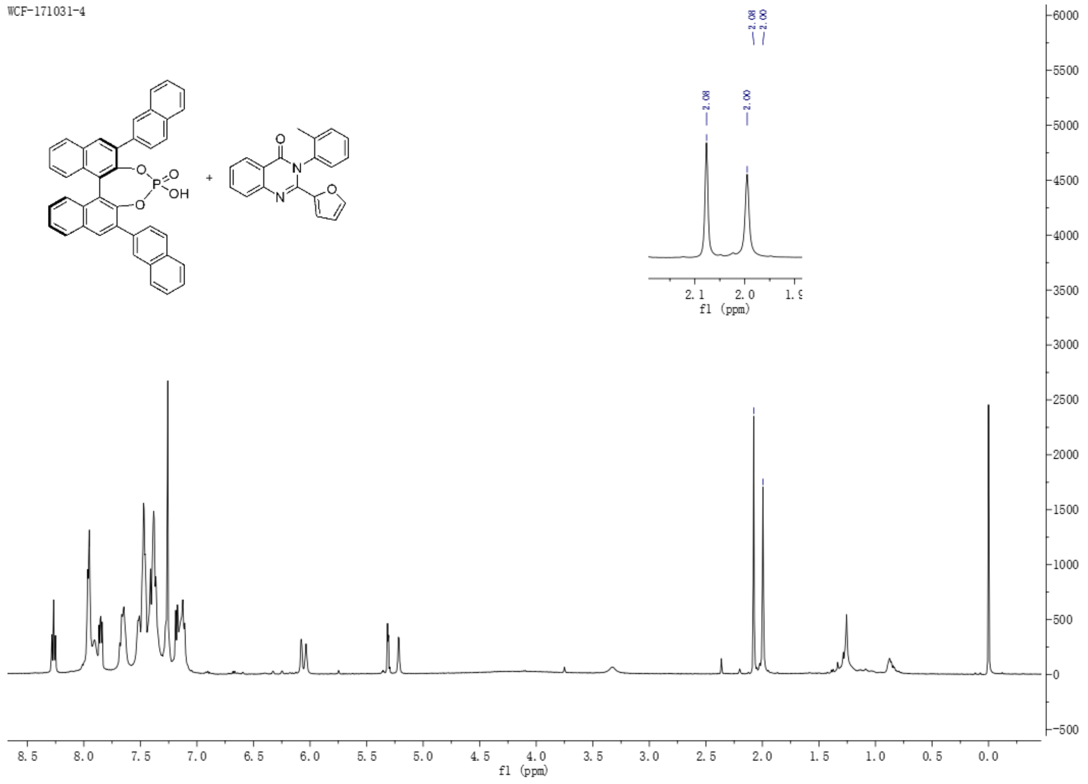

5.5. ^{1}H NMR (500 MHz, $CDCl_3$) of CPA 1d and guest 2a.

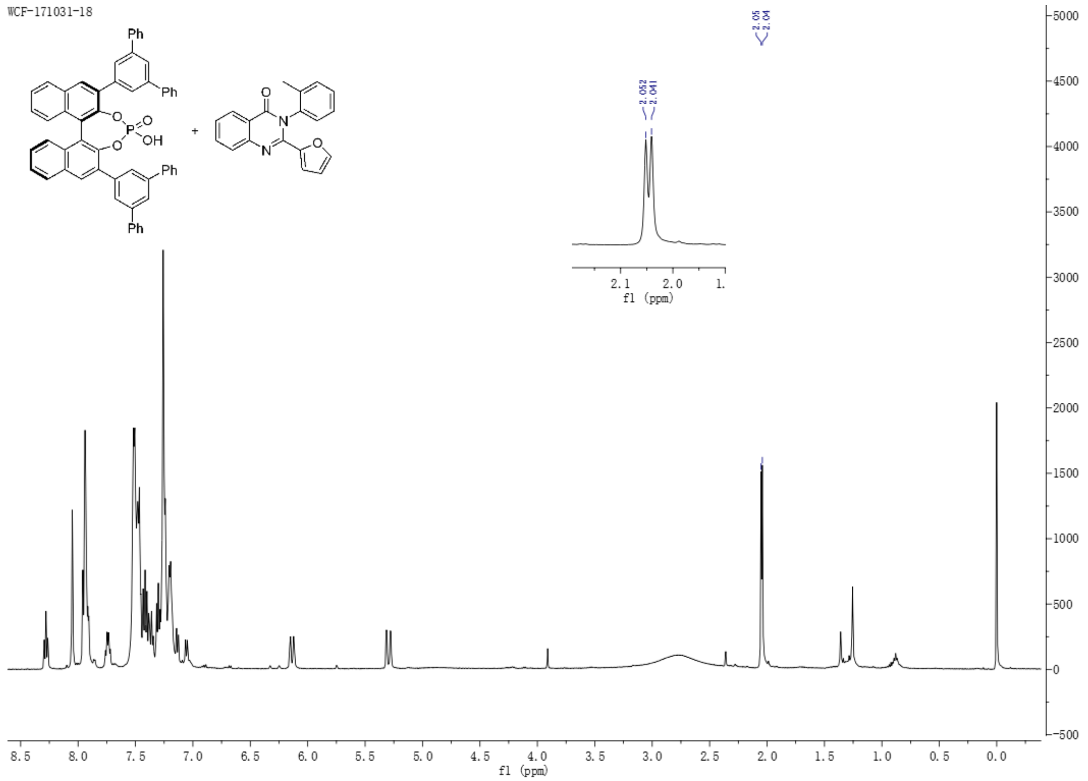

5.6.^{1}H NMR (500 MHz, $CDCl_3$) of CPA 1e and guest 2a.

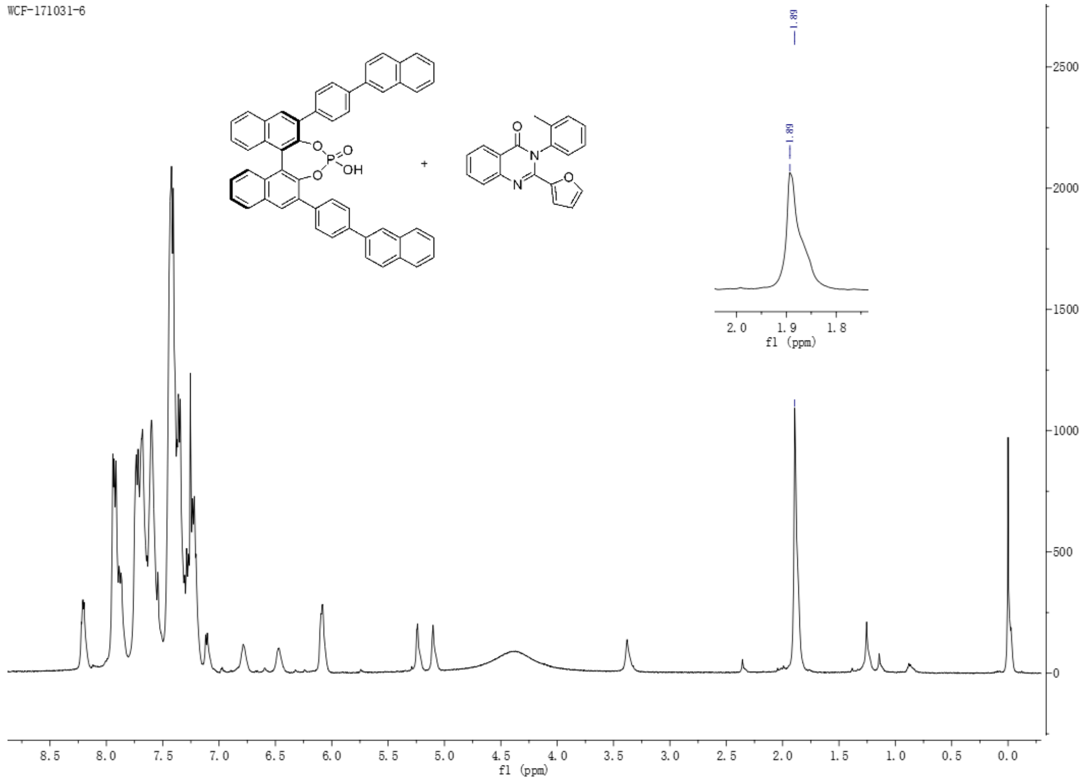

5.7. ^{1}H NMR (500 MHz, CPA 1d) of CPA 1f and guest 2a.

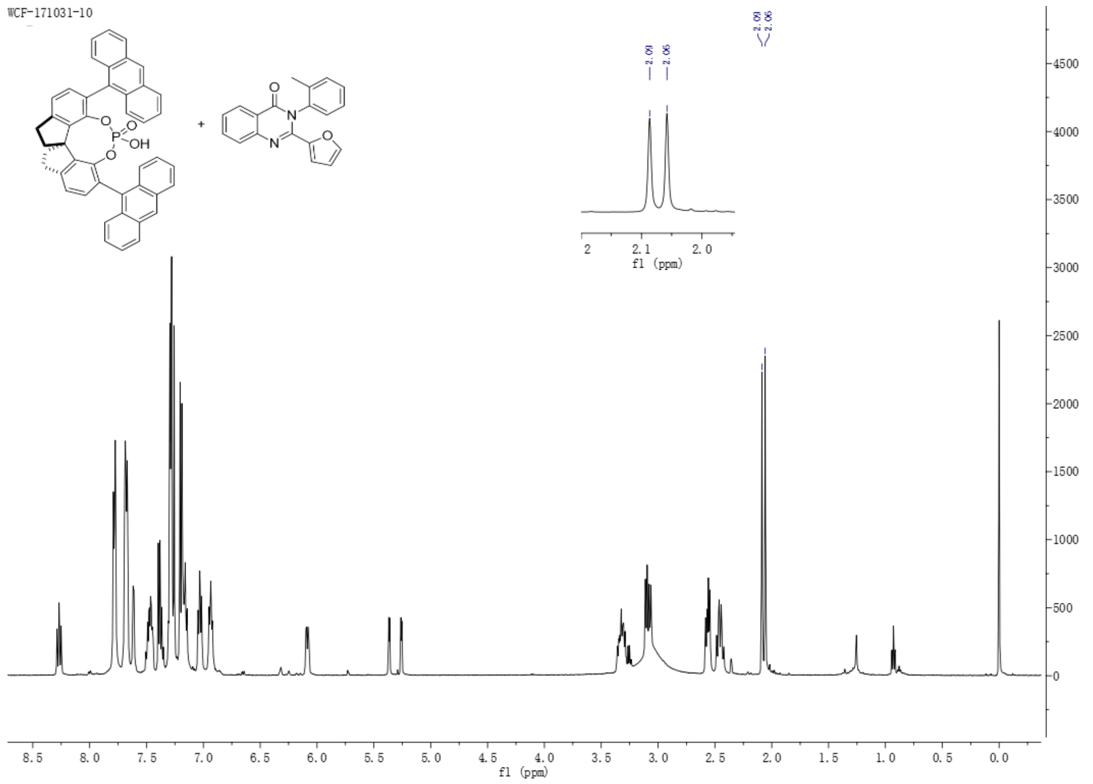

5.8. ^{1}H NMR (500 MHz, $CDCl_3$) of CPA 1g and guest 2a.

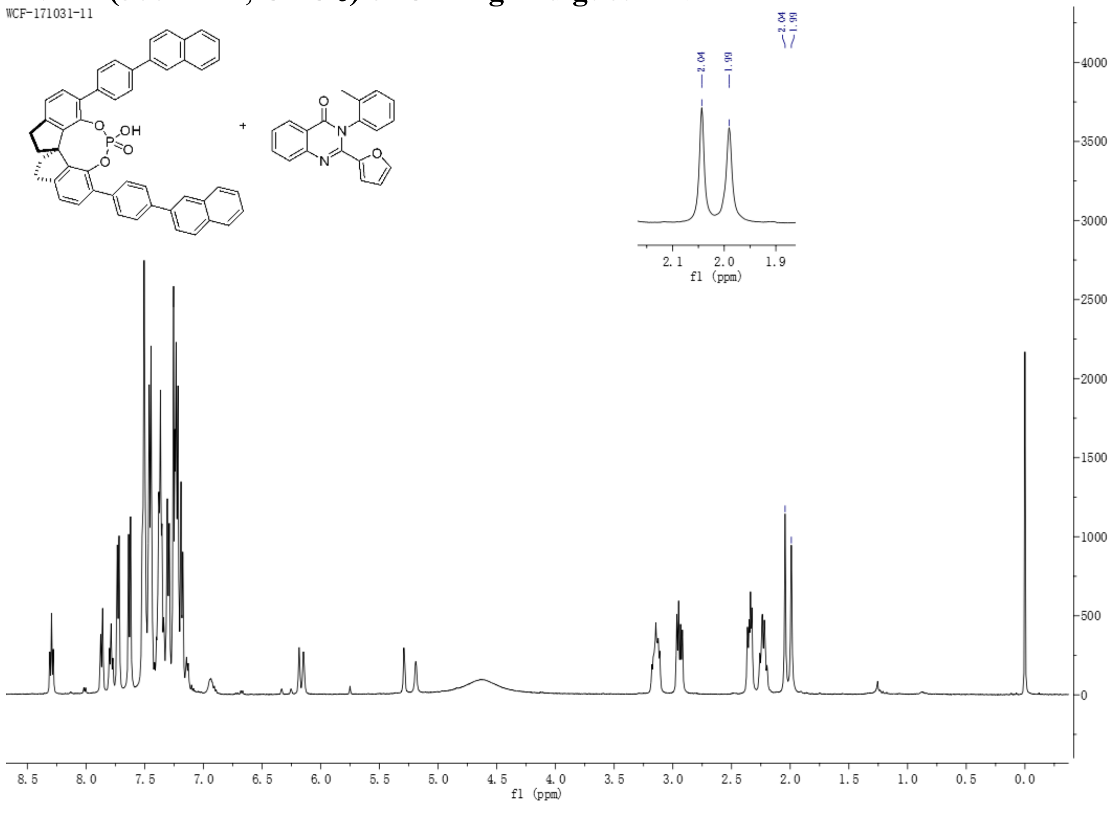

5.9. ^{1}H NMR (500 MHz, CDCl₃) of CPA 1h and guest 2a.

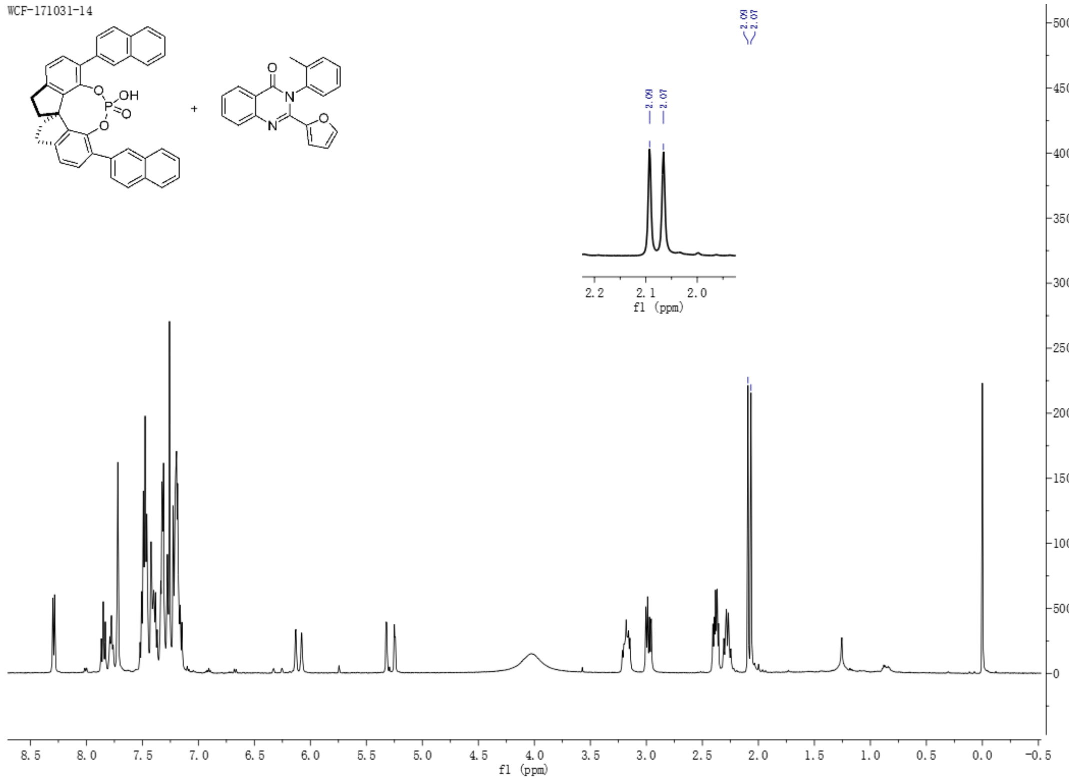

5.10. ^{1}H NMR (500 MHz, CDCl₃) of CPA 1i and guest 2a.

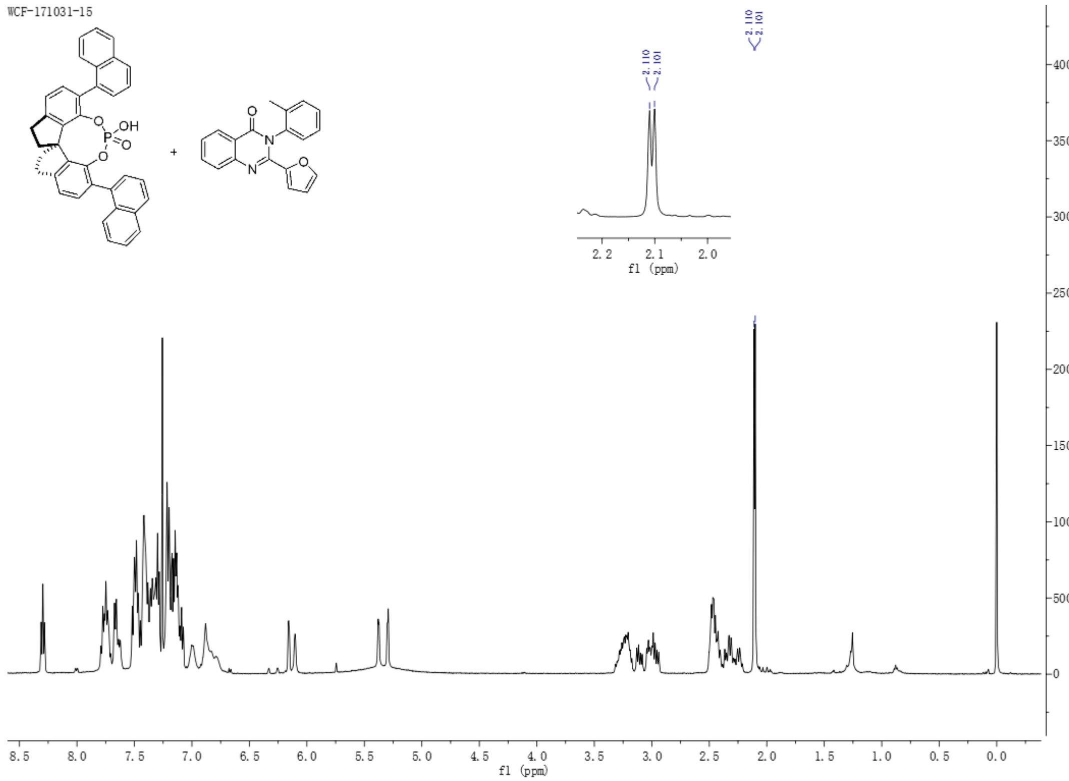

5.11. ^{1}H NMR (500 MHz, $CDCl_3$) of CPA 1j and guest 2a.

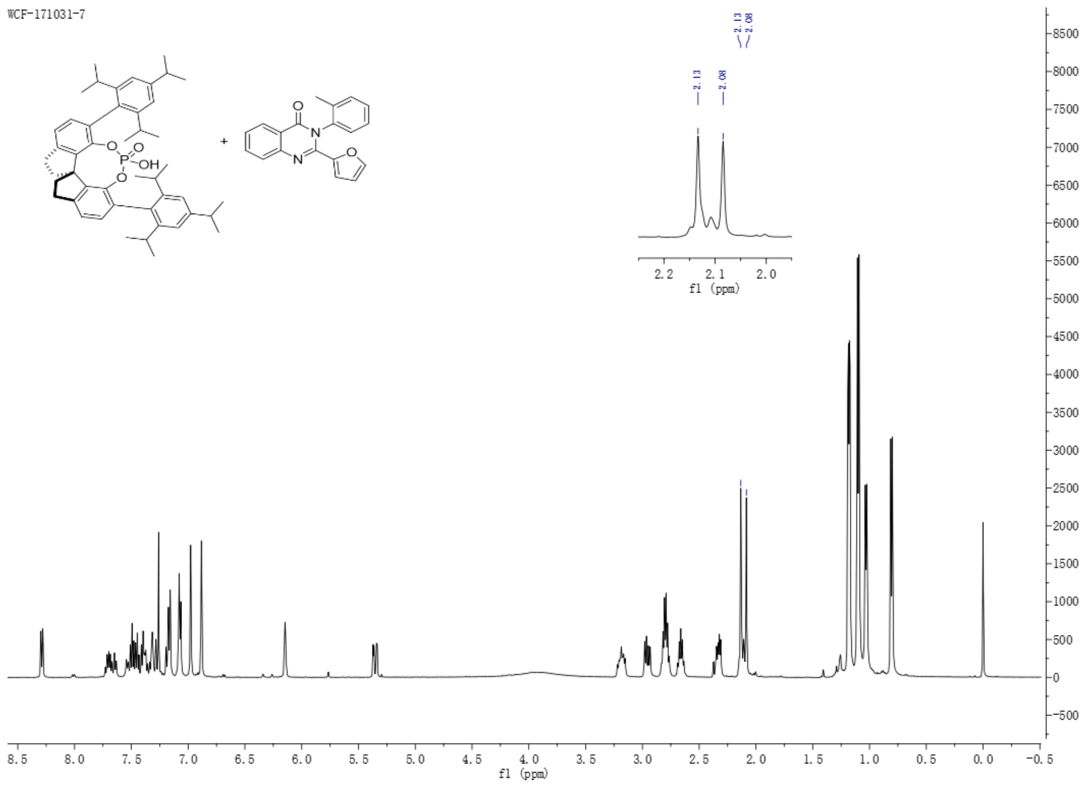

5.12. ^{1}H NMR (500 MHz, $CDCl_3$) of CPA 1k and guest 2a.

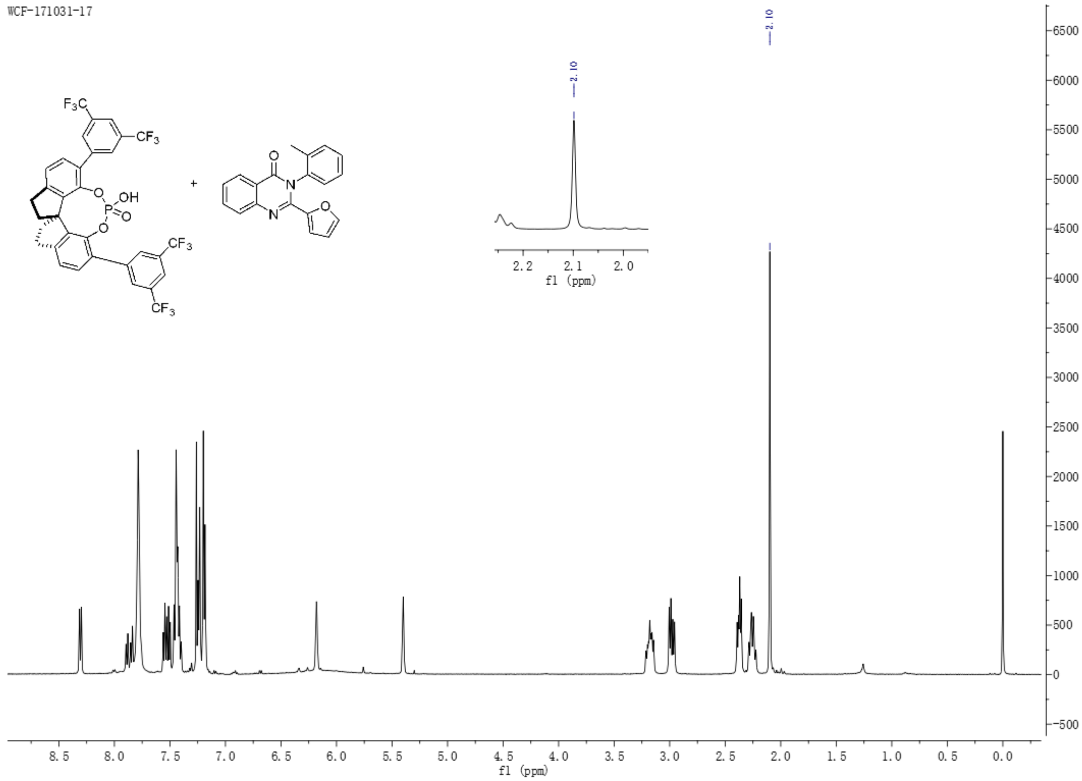

5.13. ^{1}H NMR (500 MHz, $CDCl_3$) of CPA 1l and guest 2a.

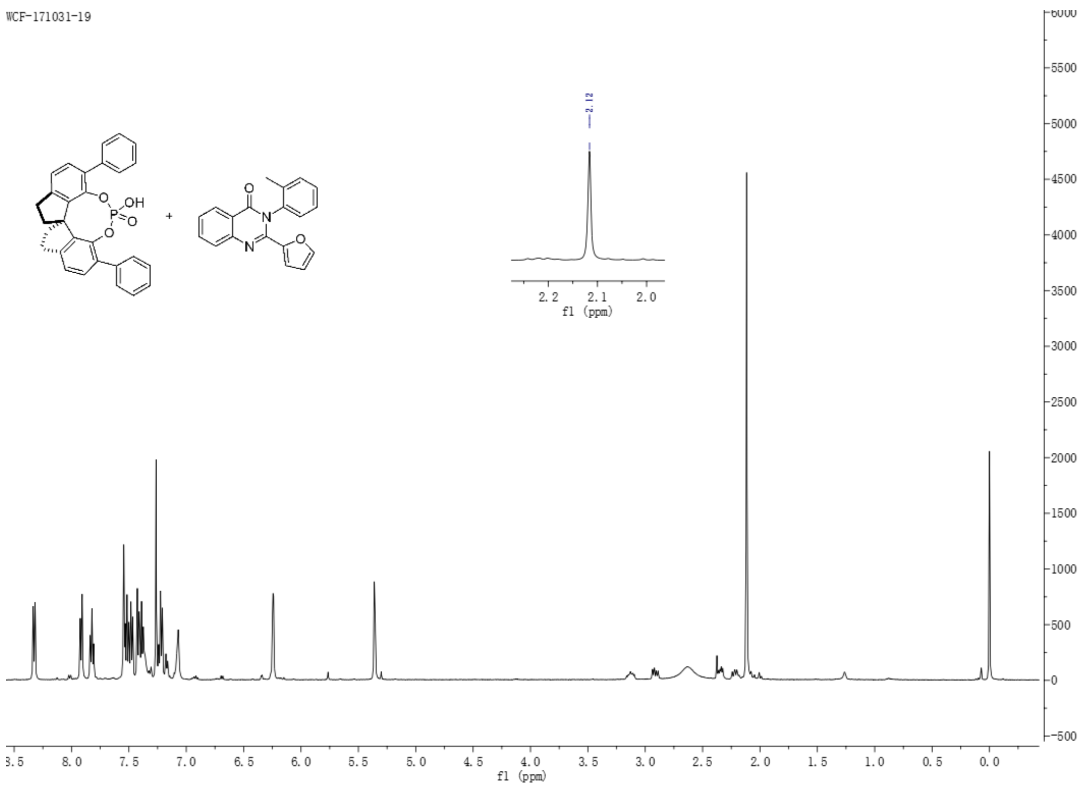

5.14. ^{1}H NMR (500 MHz, DMSO-D6) of CPA 1a and guest 2a.

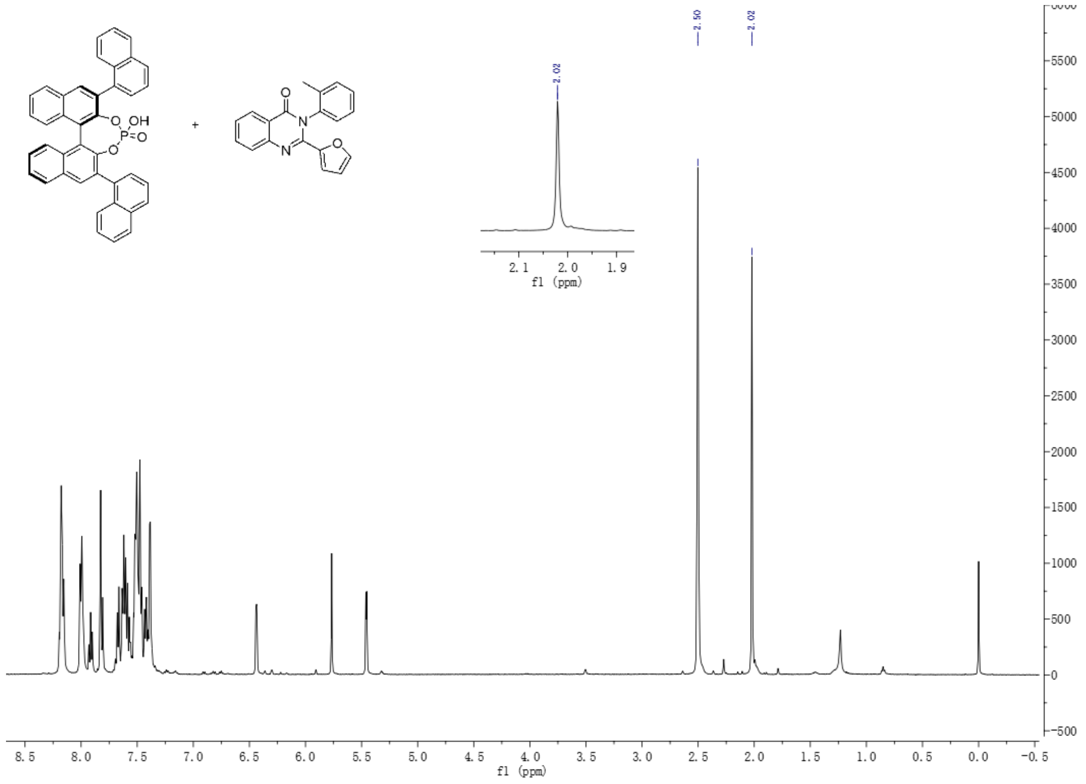

5.15. ^{1}H NMR (500 MHz, CD3OD) of CPA 1a and guest 2a.

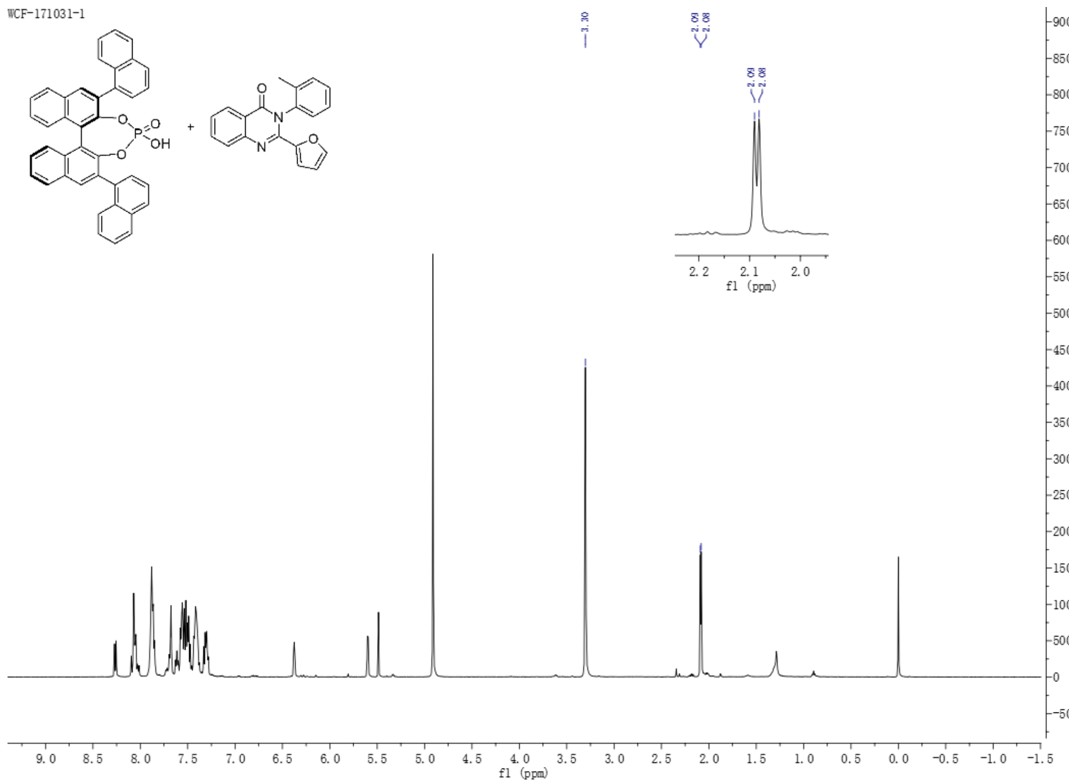

5.16. ^{1}H NMR (500 MHz, ACETONE-D6) of CPA 1a and guest 2a.

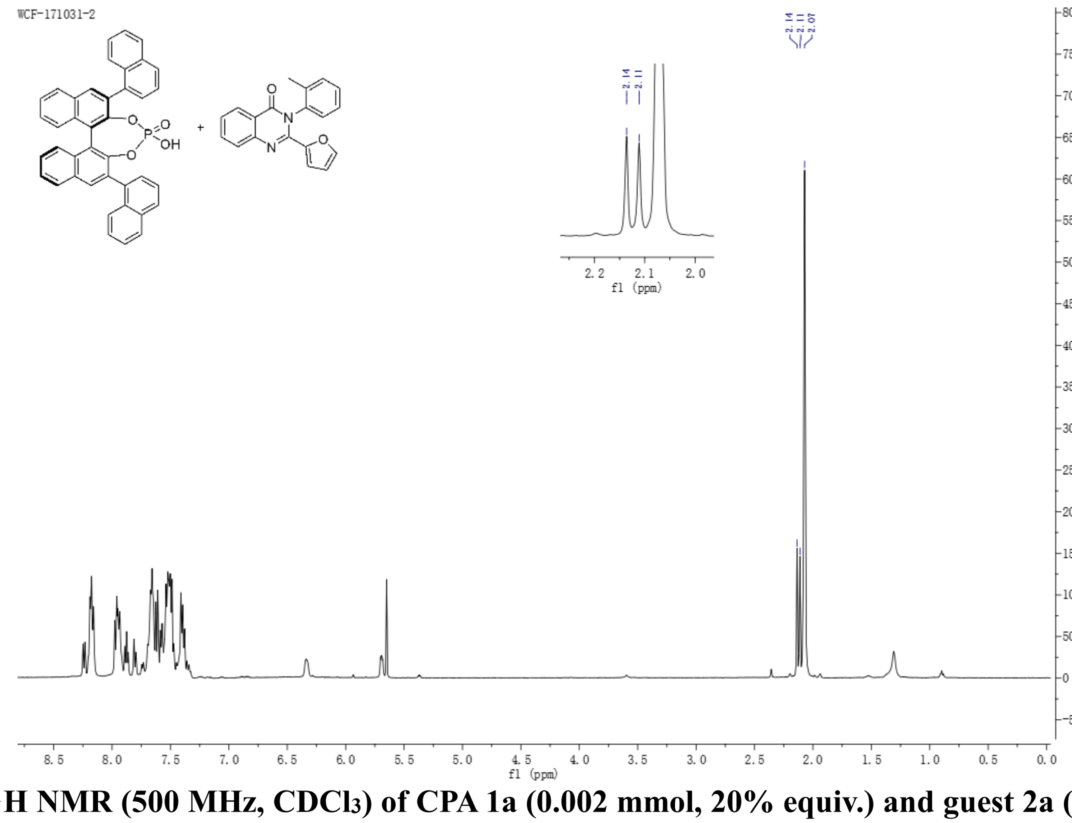

5.17. ^{1}H NMR (500 MHz, CDCl3) of CPA 1a (0.002 mmol, 20% equiv.) and guest 2a (0.01 mmol, 1.0 equiv.).

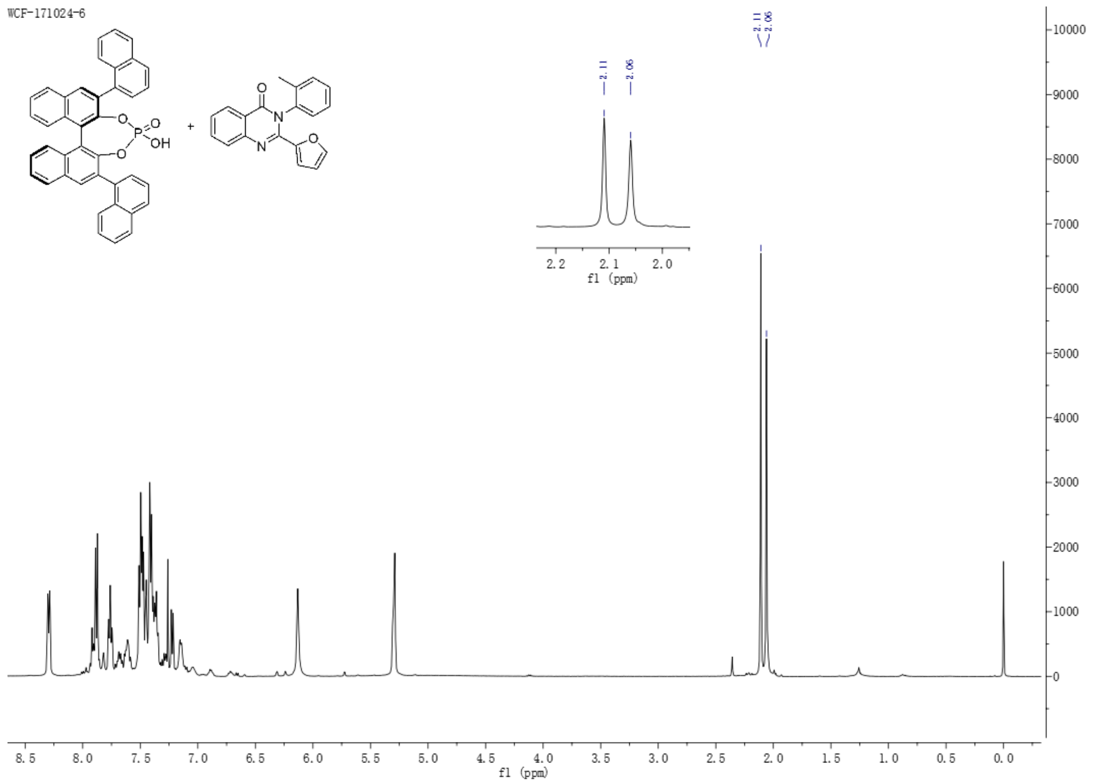

5.18. ^{1}H NMR (500 MHz, $CDCl_3$) of CPA 1a (0.005 mmol, 50% equiv.) and guest 2a (0.01 mmol, 1.0 equiv.).

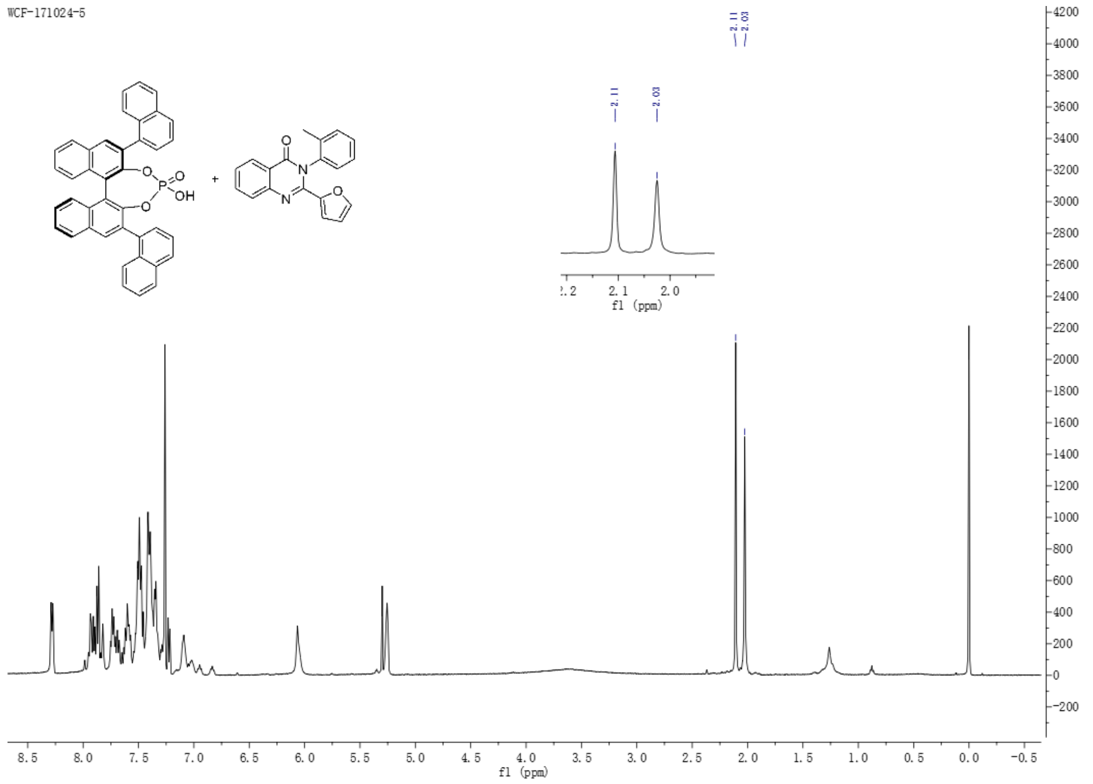

5.19. ^{1}H NMR (500 MHz, $CDCl_3$) of CPA 1a (0.015 mmol, 1.5 equiv.) and guest 2a (0.01 mmol, 1.0 equiv.).

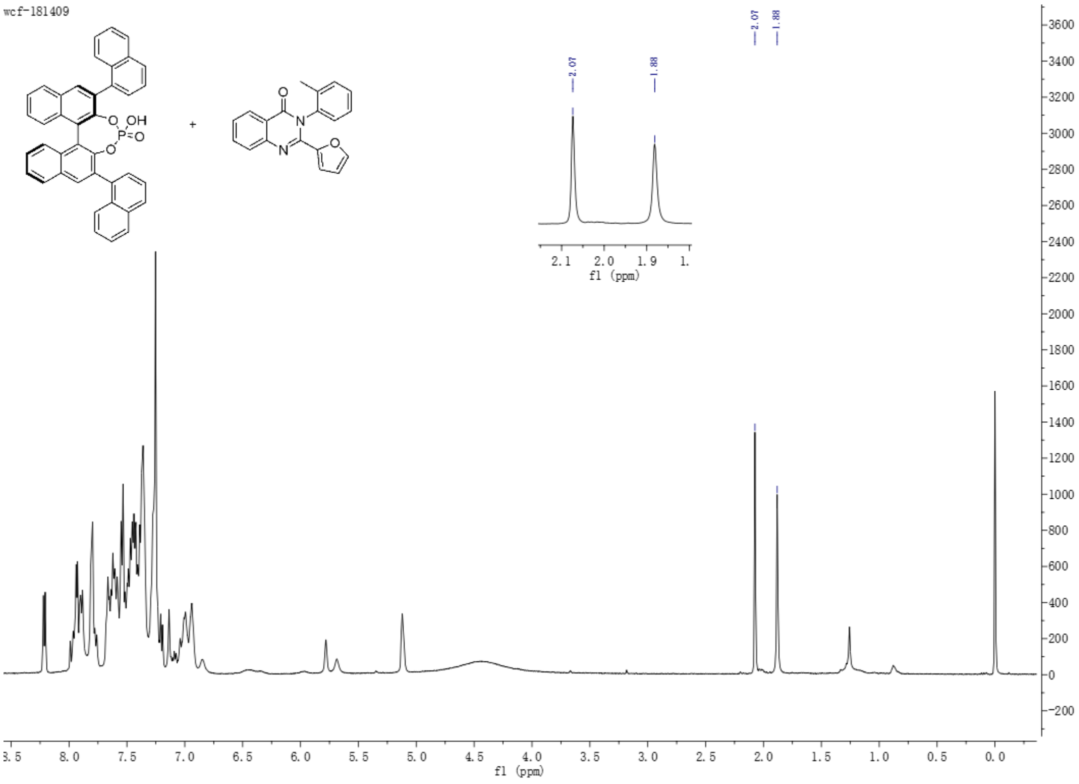

6. ^{1}H NMR spectra of 1a (0.01 mmol, 1.0 equiv.) and racemic 2 (0.01 mmol, 1.0 equiv.).

6.1. ^{1}H NMR (500 MHz, $CDCl_3$) of racemic guest 2b.

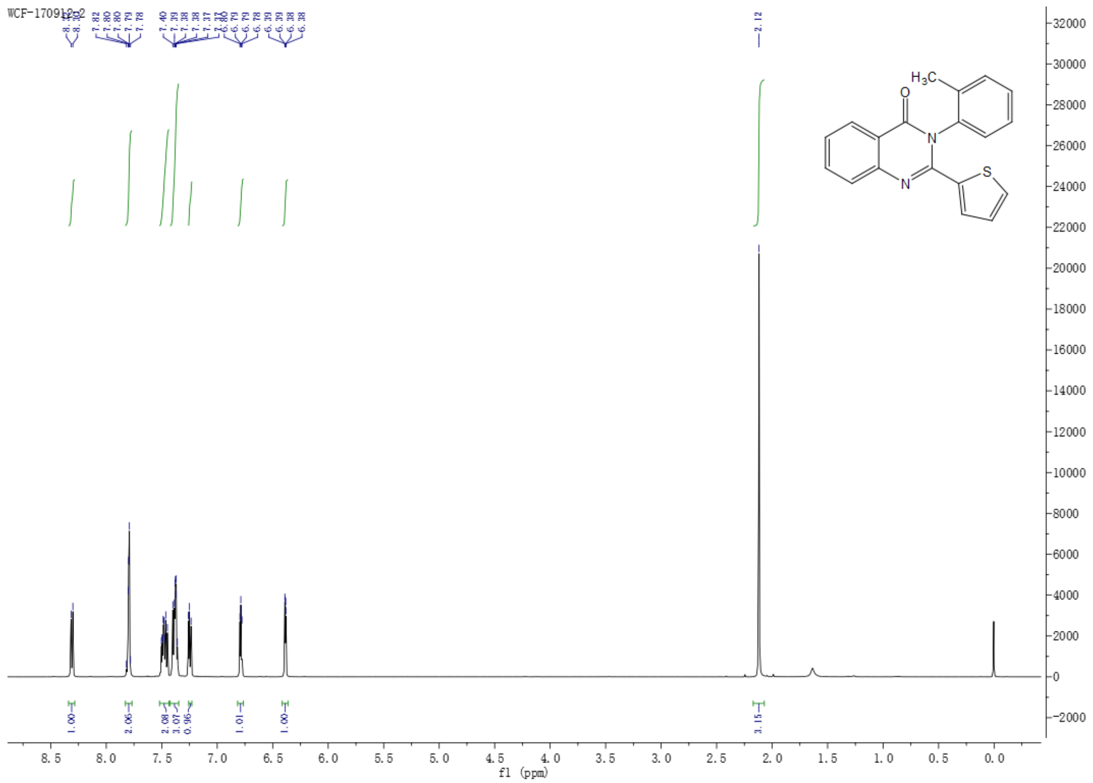

6.2. ^{1}H NMR (500 MHz, $CDCl_3$) of CPA 1a and racemic guest 2b.

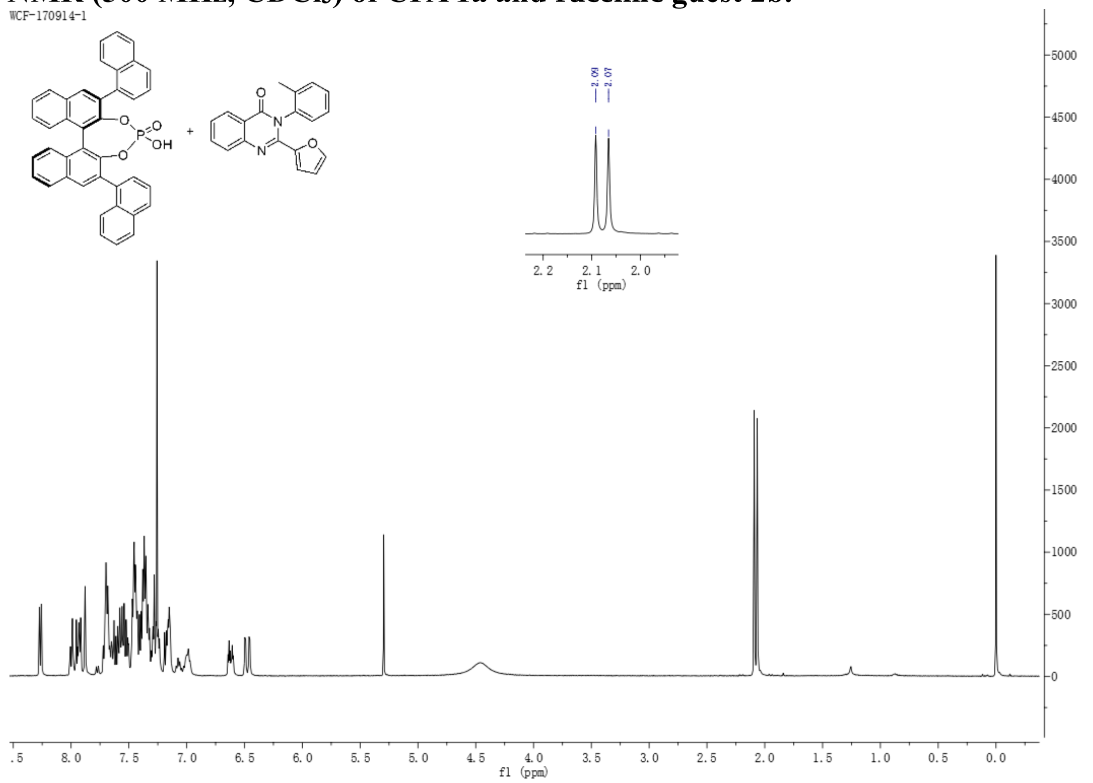

6.3. ^{1}H NMR (500 MHz, $CDCl_3$) of racemic 2c.

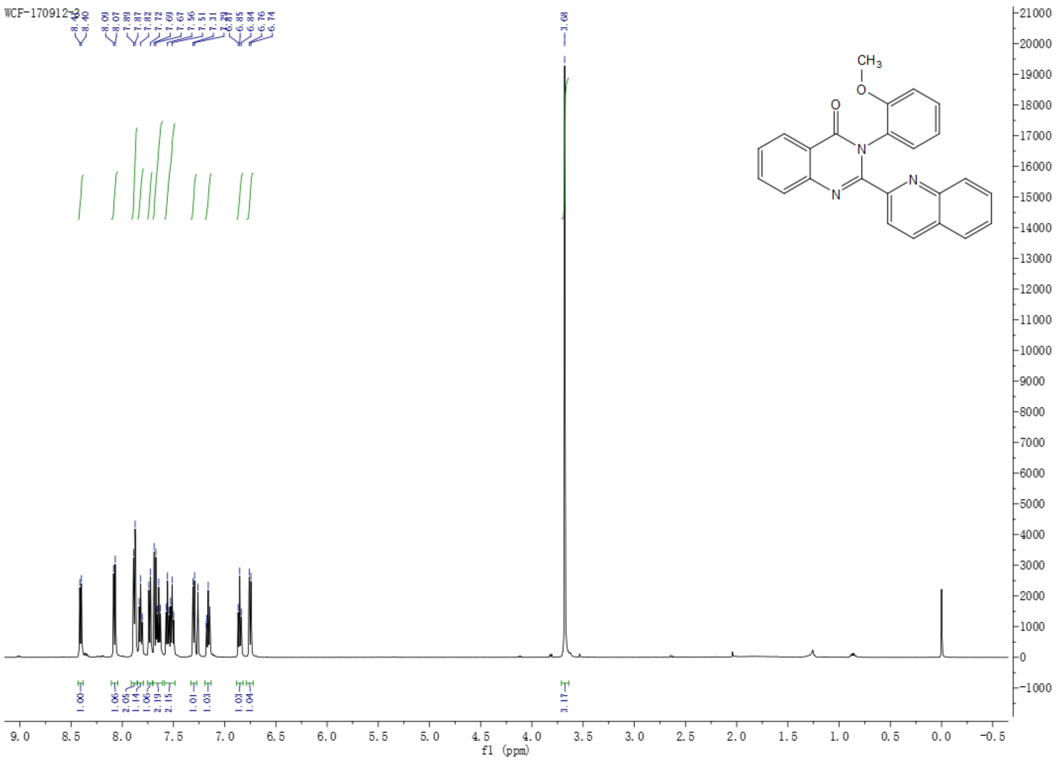

6.4. ^{1}H NMR (500 MHz, $CDCl_3$) of CPA 1a and racemic guest 2c.

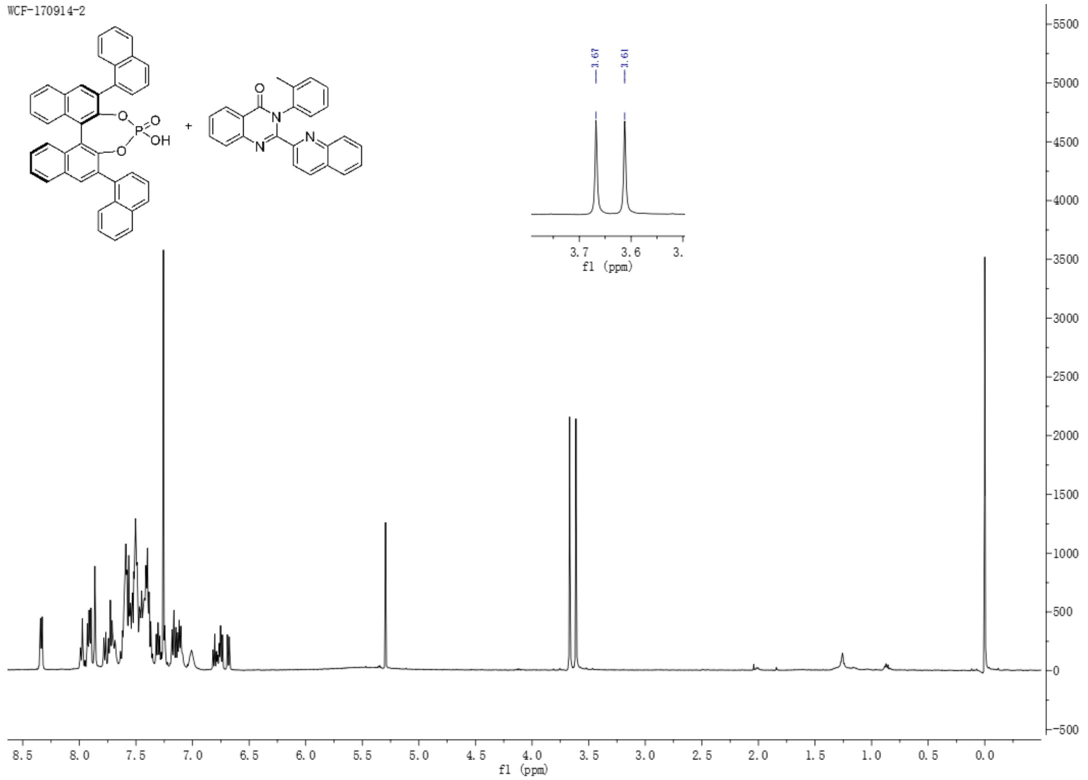

6.5. ^{1}H NMR (500 MHz, $CDCl_3$) of racemic 2d.

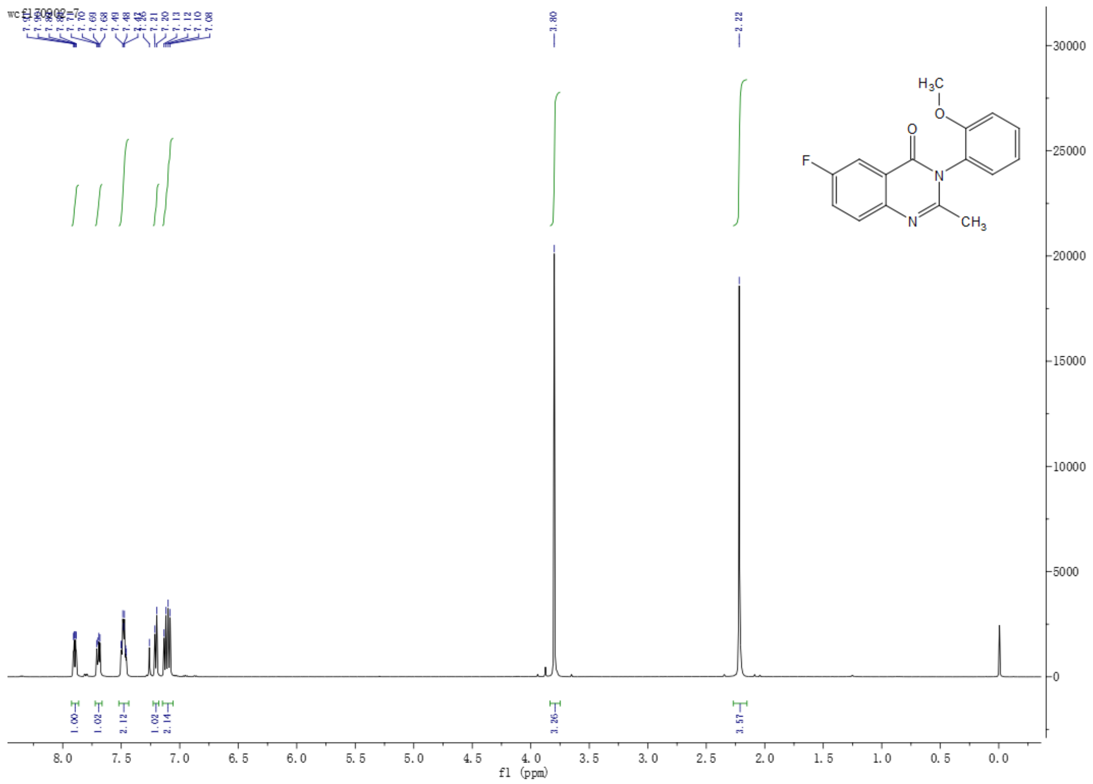

6.6. ¹H NMR (500 MHz, $CDCl_3$) of CPA 1a and racemic guest 2d.

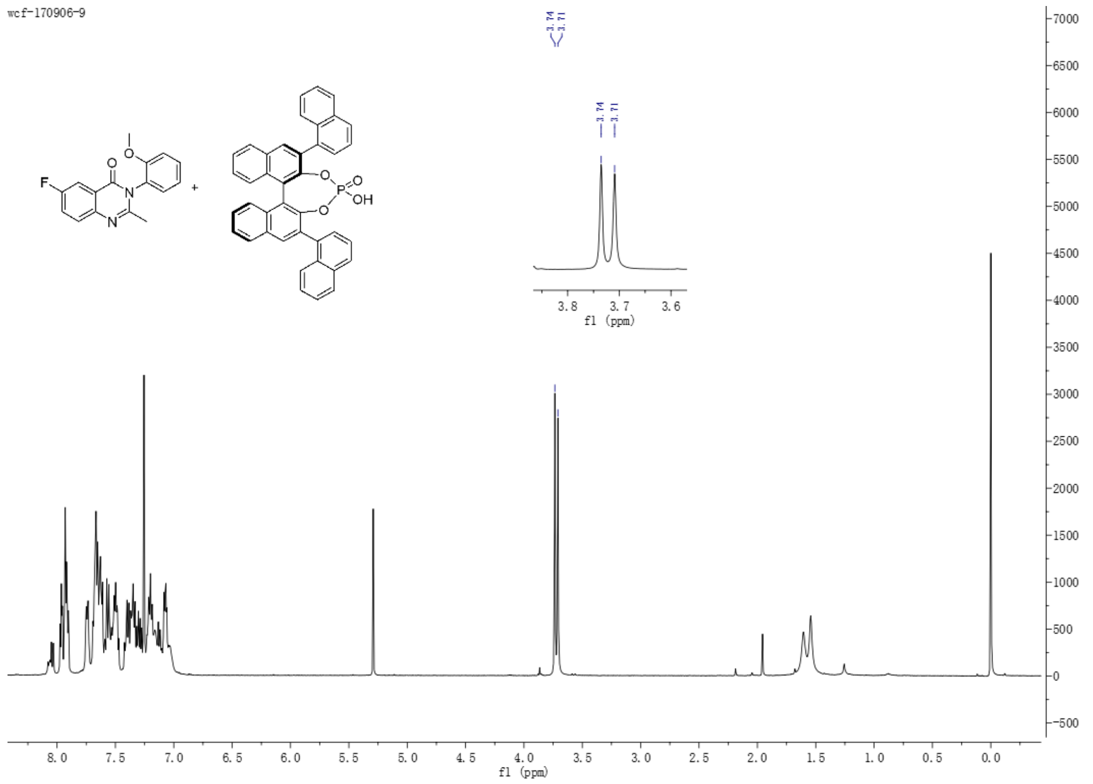

6.7. ¹H NMR (500 MHz, $CDCl_3$) of racemic 2e.

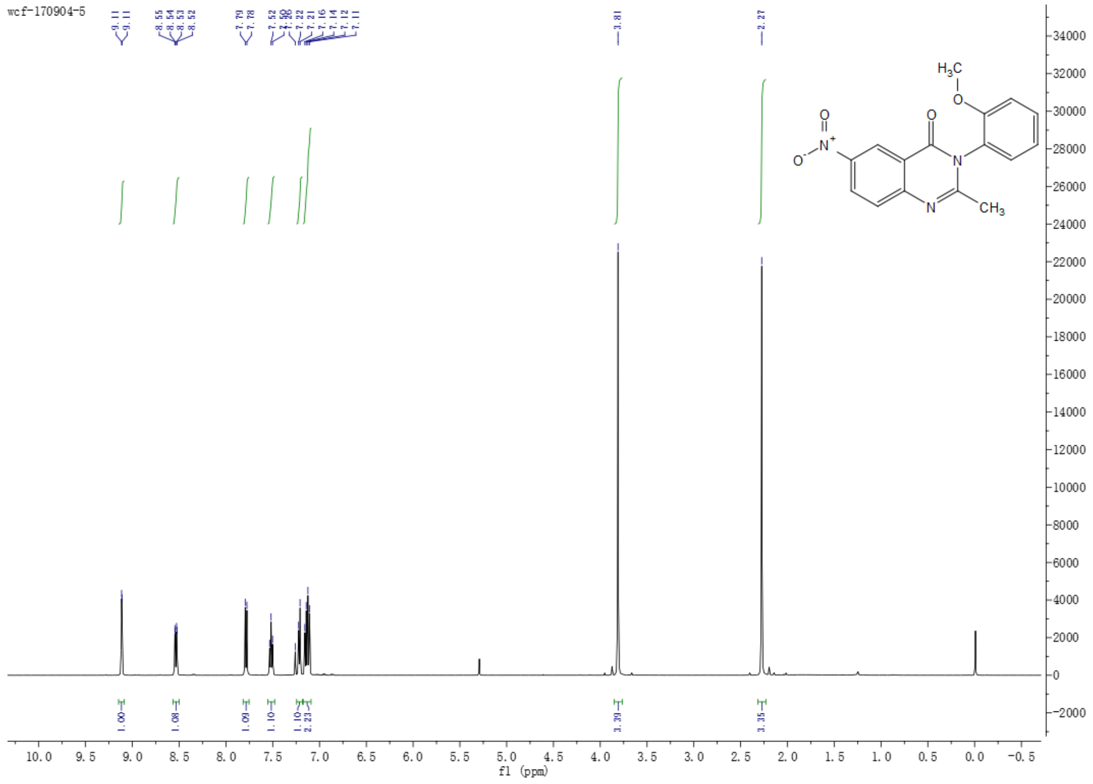

6.8. ^{1}H NMR (500 MHz, $CDCl_3$) of CPA 1a and racemic guest 2e.

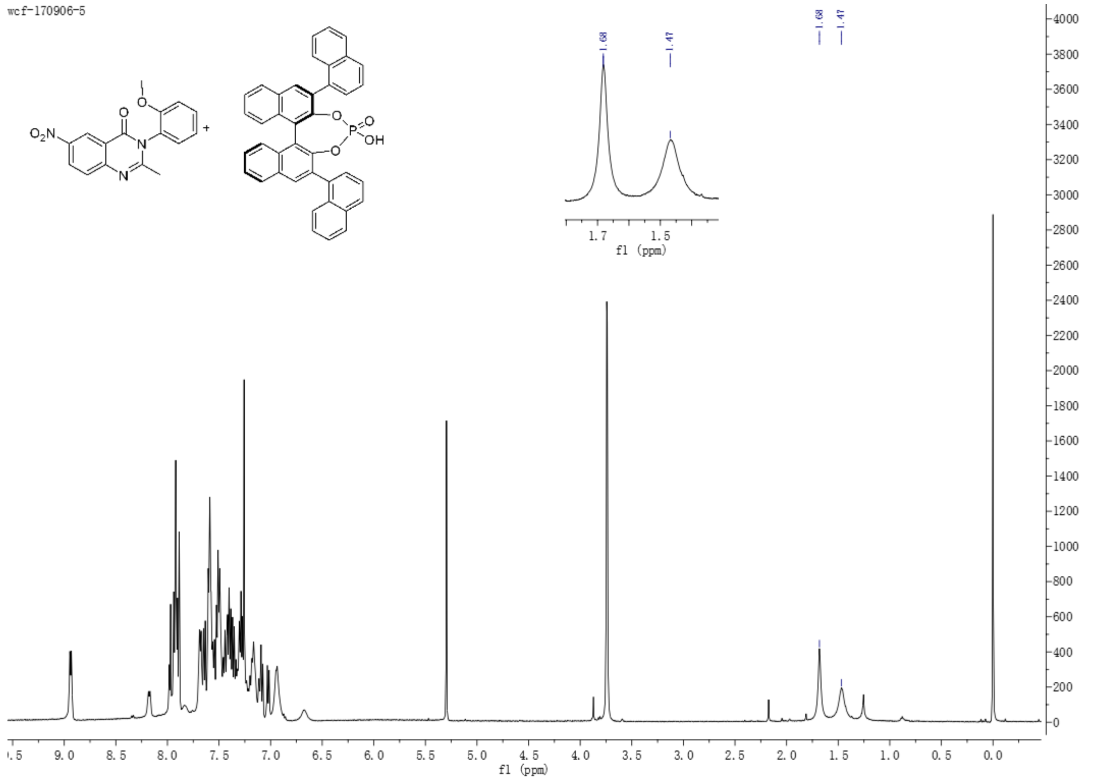

6.9. ^{1}H NMR (500 MHz, $CDCl_3$) of racemic 2f.

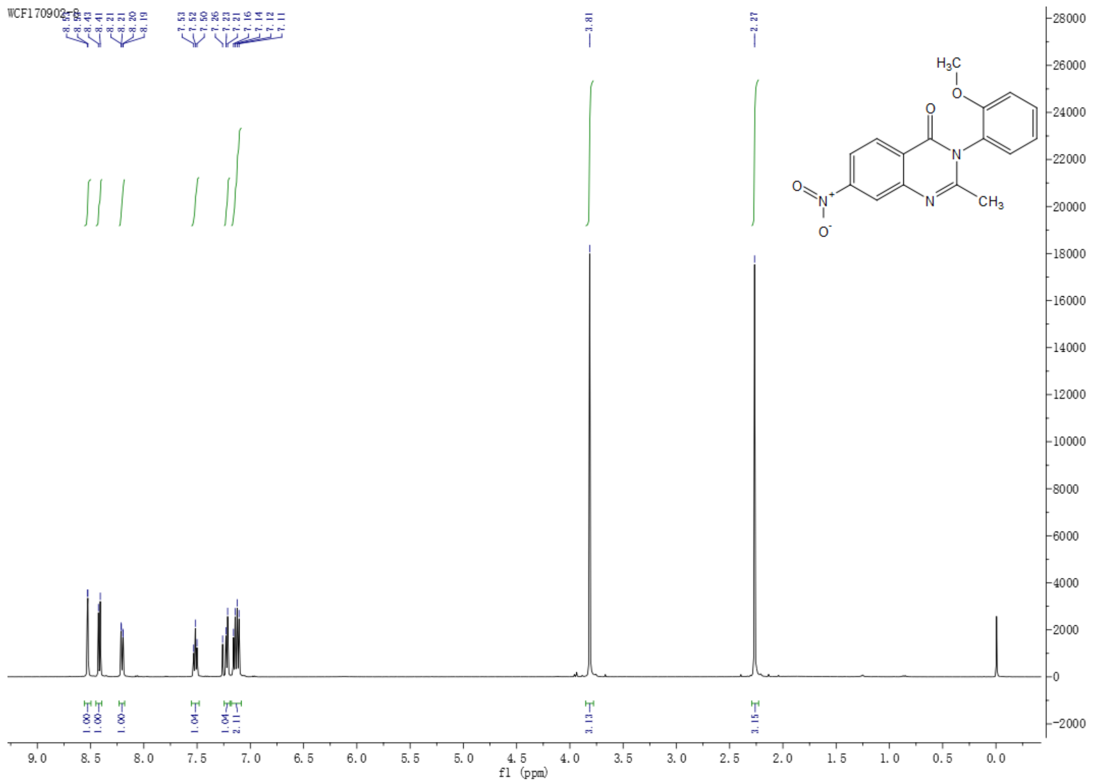

6.10. ^{1}H NMR (500 MHz, $CDCl_3$) of CPA 1a and racemic guest 2f.

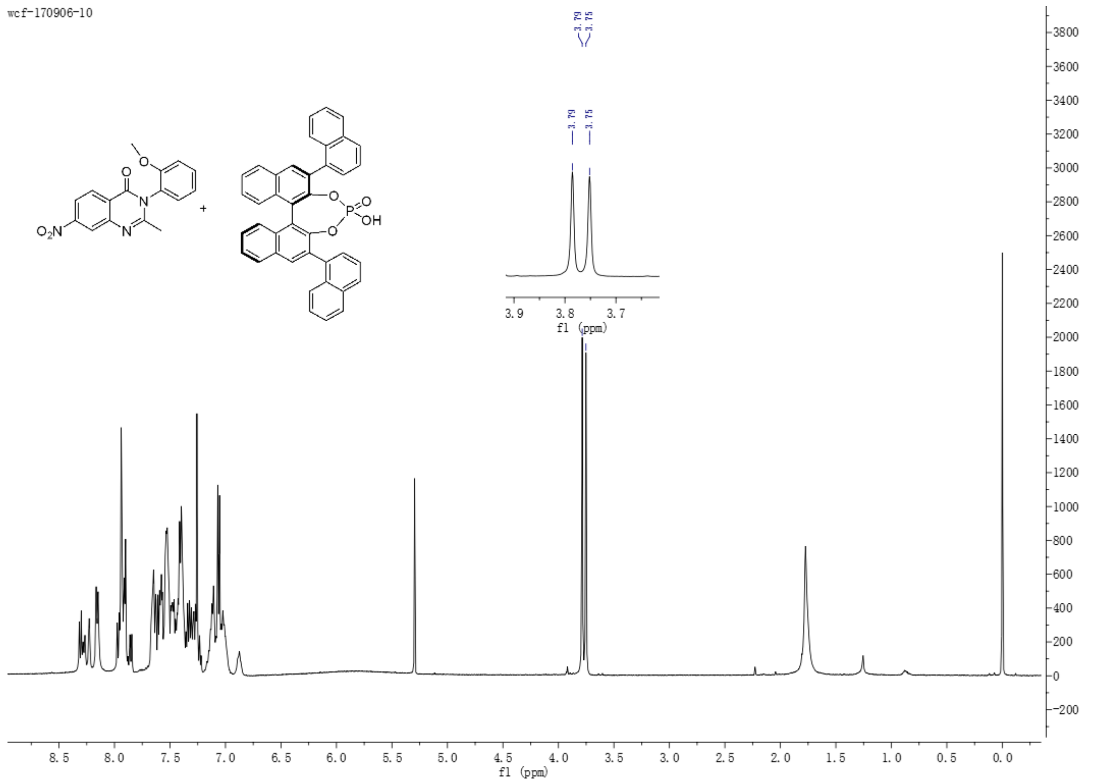

6.11. ^{1}H NMR (500 MHz, $CDCl_3$) of racemic 2g.

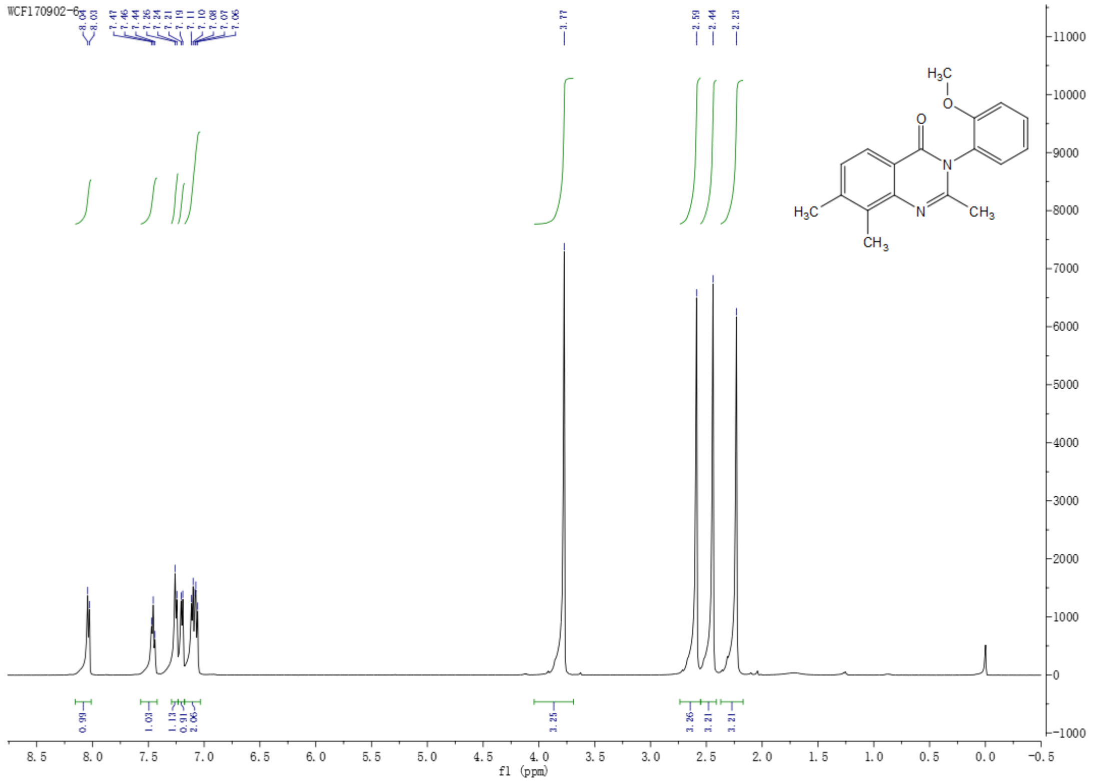

6.12. ^{1}H NMR (500 MHz, CDCl3) of CPA 1a and racemic guest 2g.

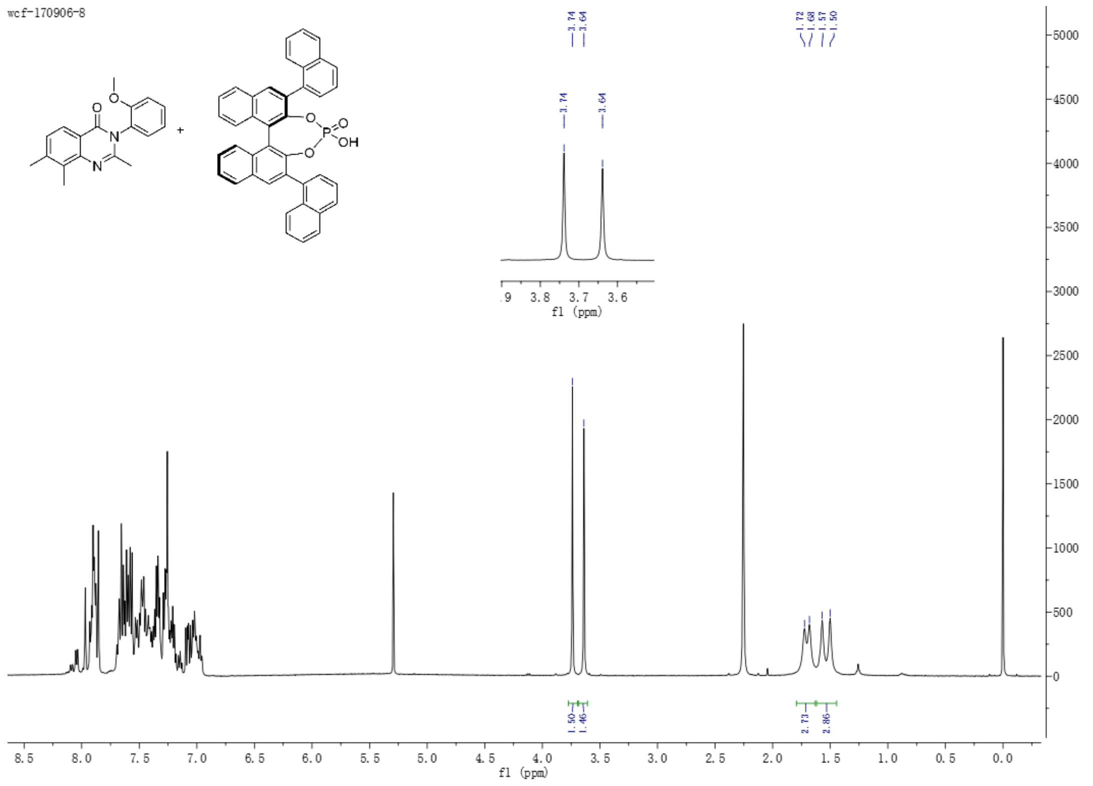

6.13. ^{1}H NMR (500 MHz, CDCl3) of racemic 2h.

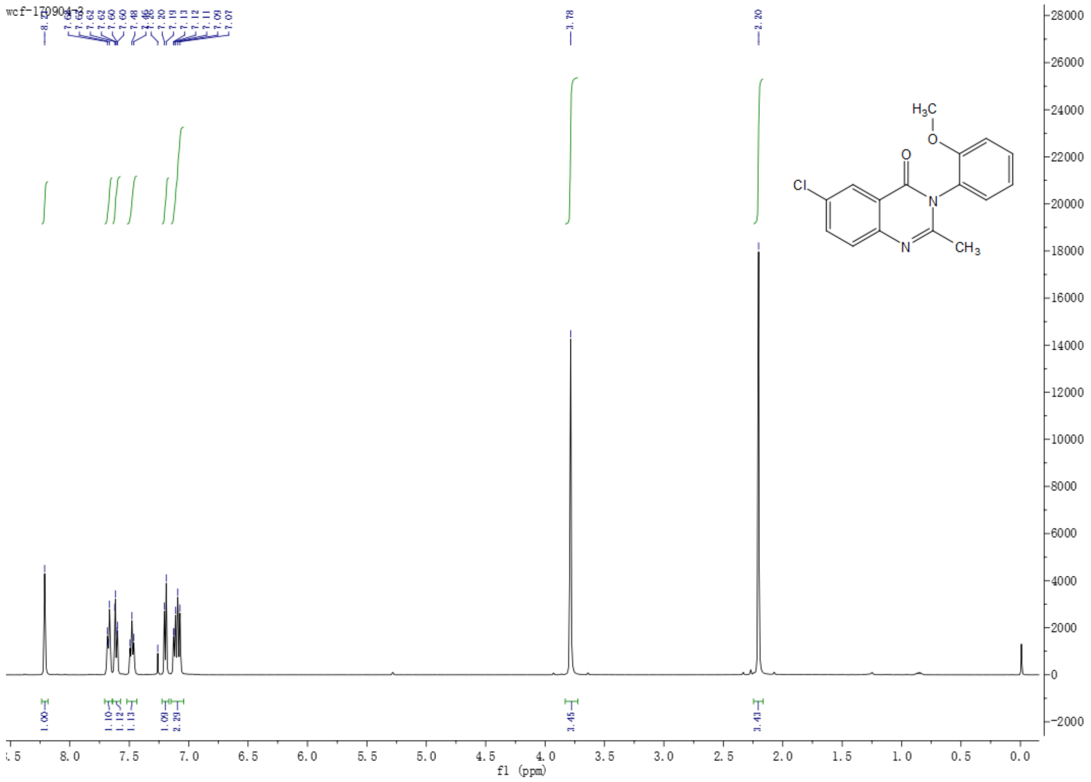

6.14. ^{1}H NMR (500 MHz, $CDCl_3$) of CPA 1a and racemic guest 2h.

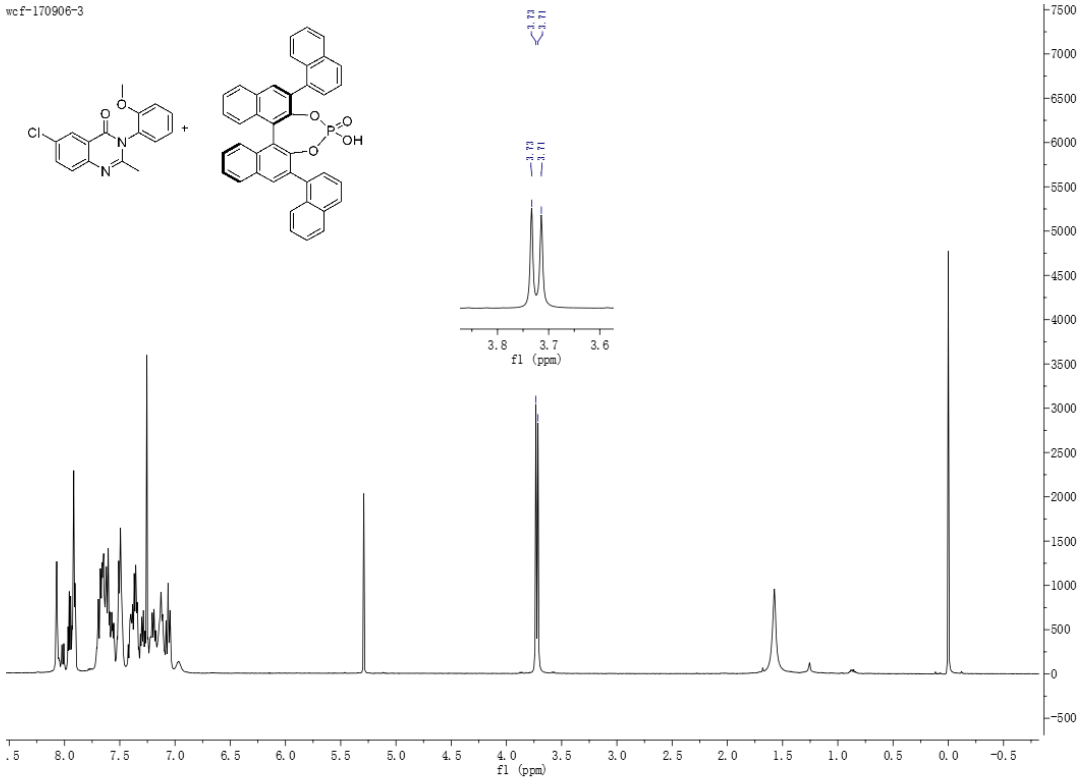

6.15. ^{1}H NMR (500 MHz, $CDCl_3$) of racemic 2i.

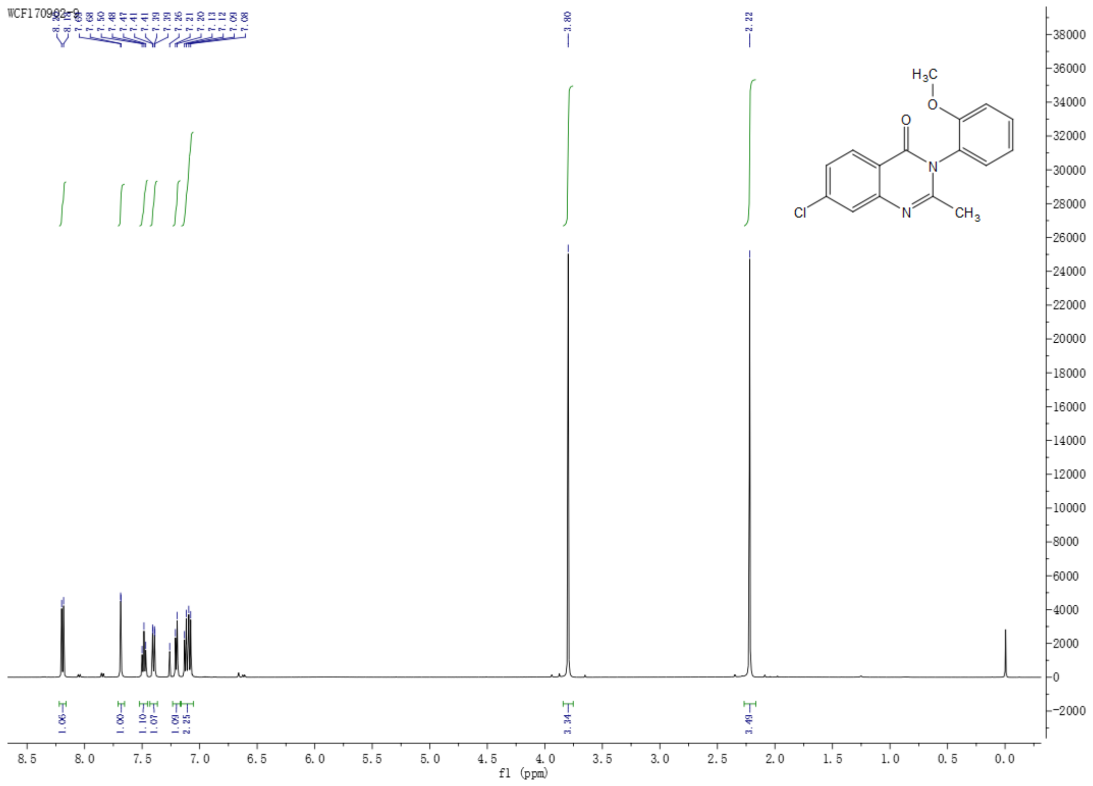

6.16. ^{1}H NMR (500 MHz, CDCl₃) of CPA 1d and racemic guest 2i.

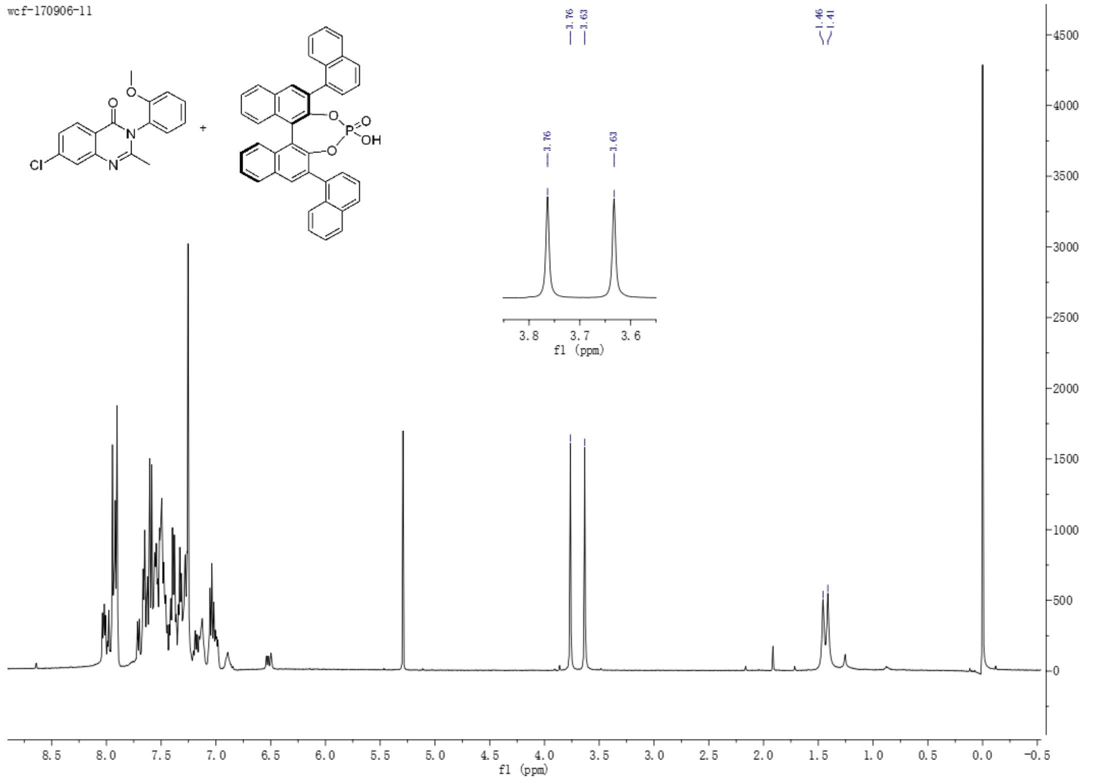

6.17. ^{1}H NMR (500 MHz, CDCl₃) of racemic 2j.

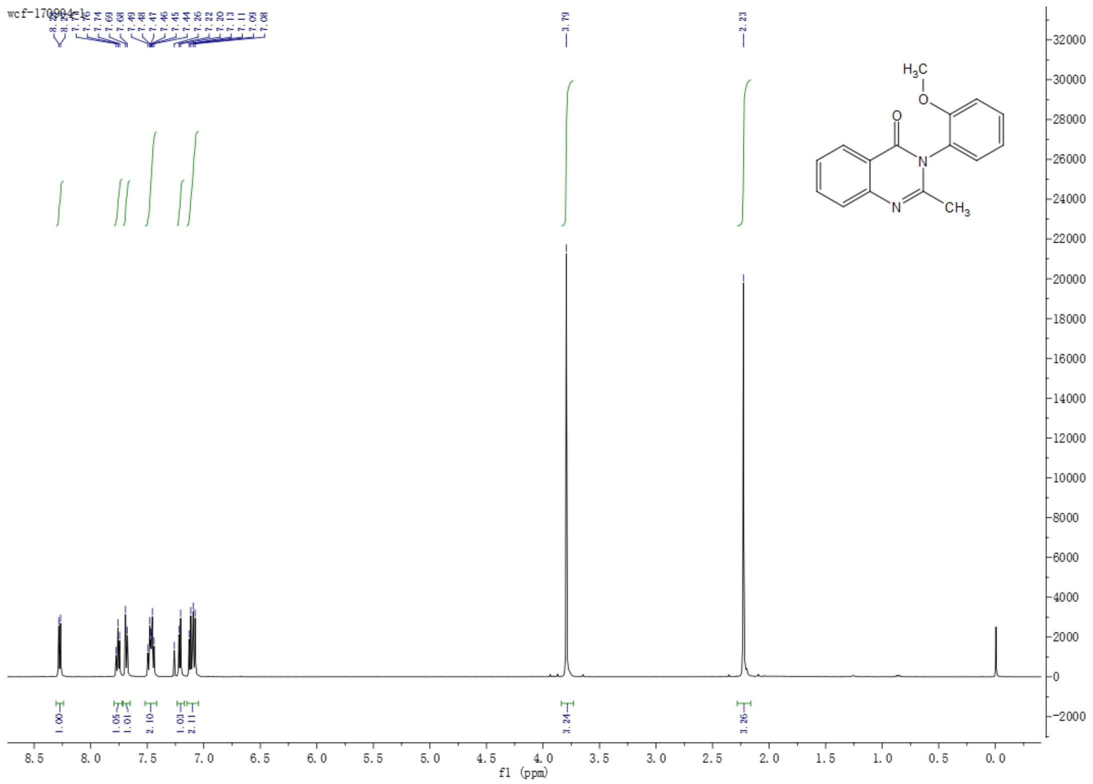

6.18. ^{1}H NMR (500 MHz, $CDCl_3$) of CPA 1a and racemic guest 2j.

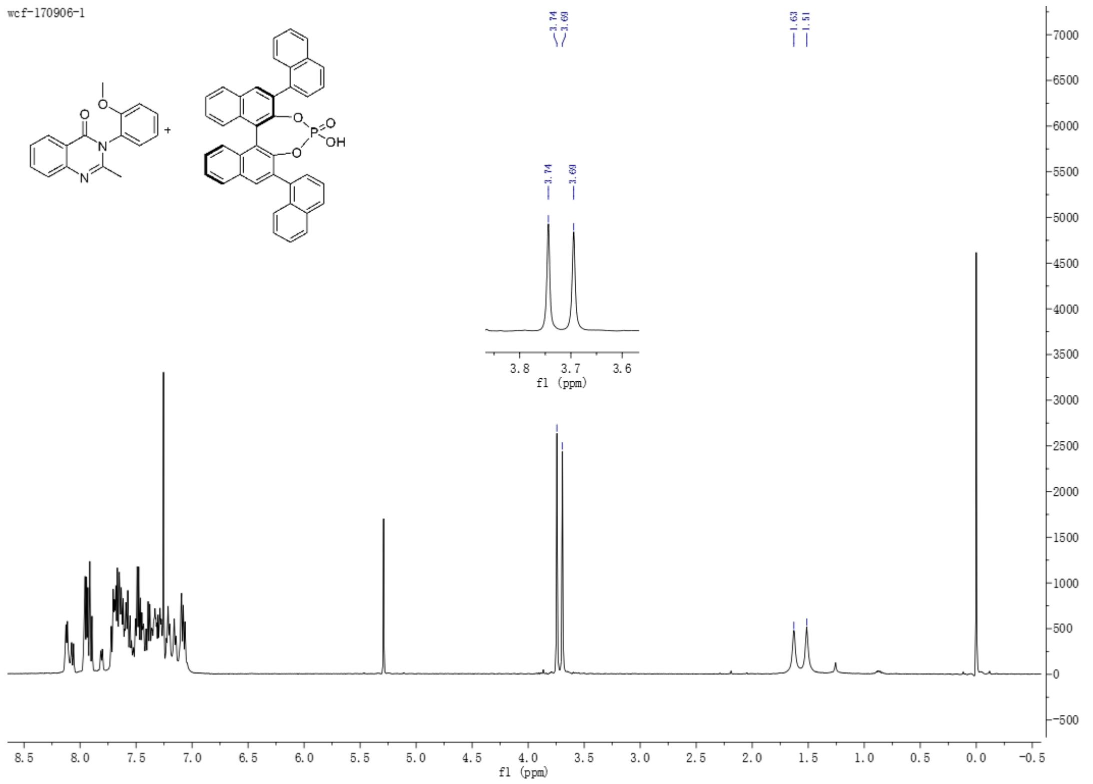

6.19. ^{1}H NMR (500 MHz, $CDCl_3$) of racemic 2k.

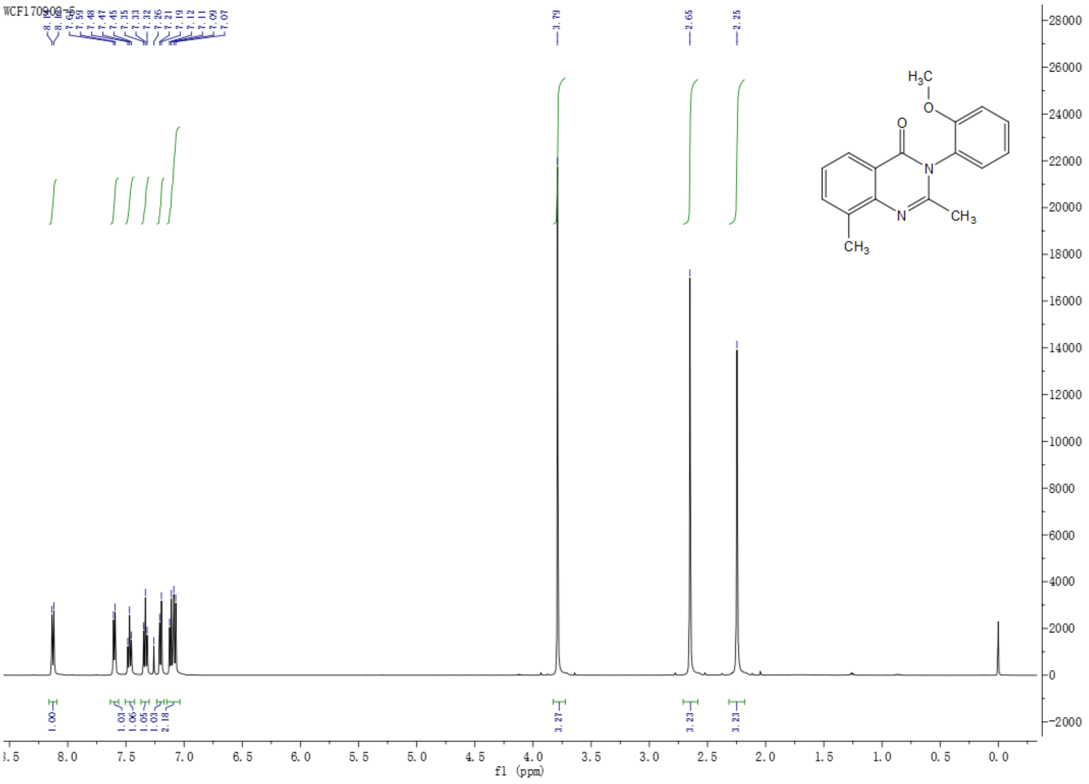

6.20. ^{1}H NMR (500 MHz, CDCl3) of CPA 1a and racemic guest 2k.

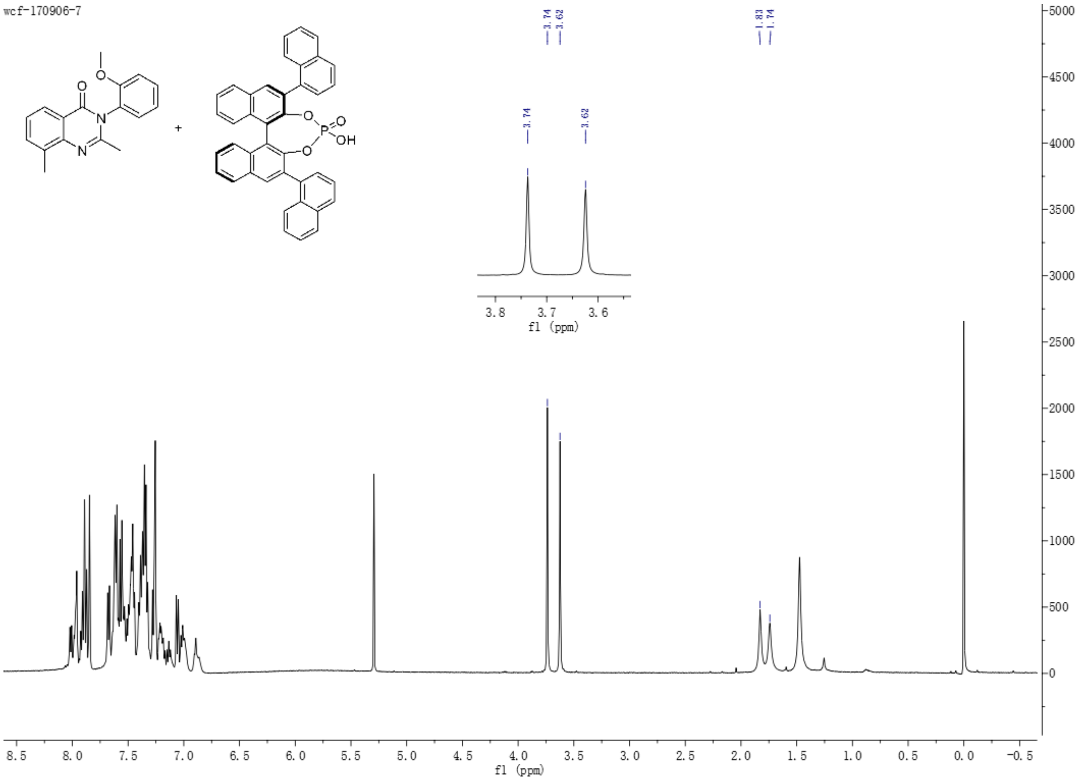

6.21. ^{1}H NMR (500 MHz, CDCl3) of racemic 2l.

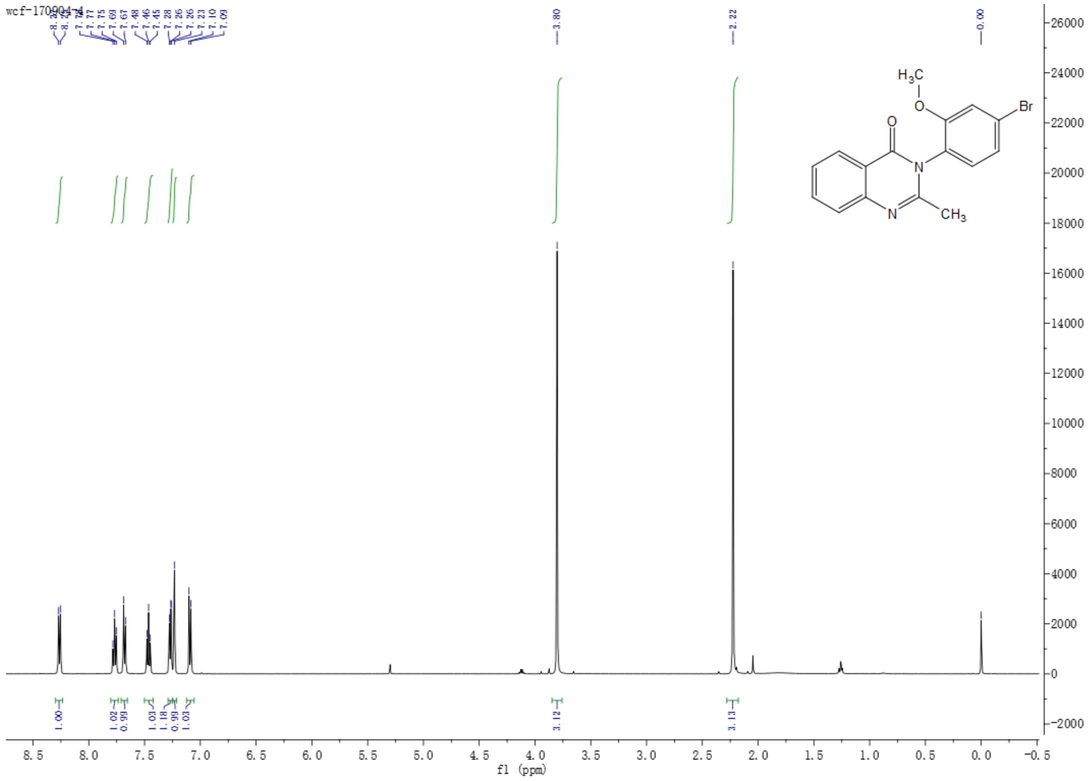

6.22. ^{1}H NMR (500 MHz, $CDCl_3$) of CPA 1a and racemic guest 2l.

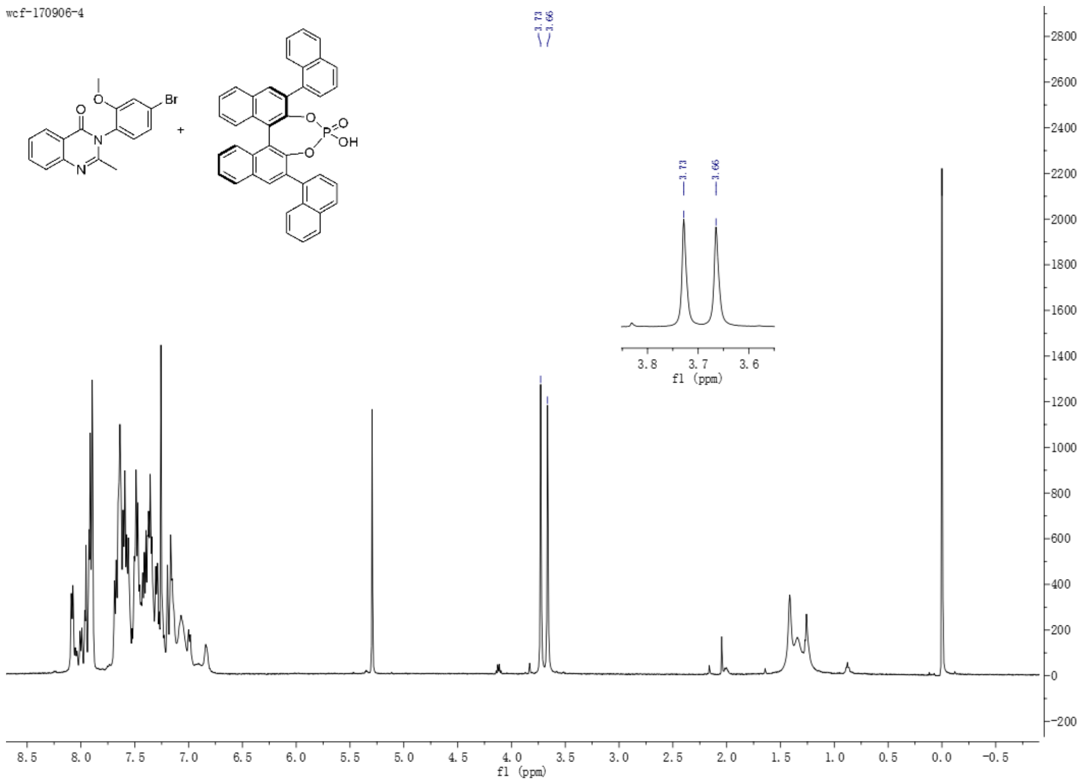

6.23. ^{1}H NMR (500 MHz, $CDCl_3$) of racemic 2m.

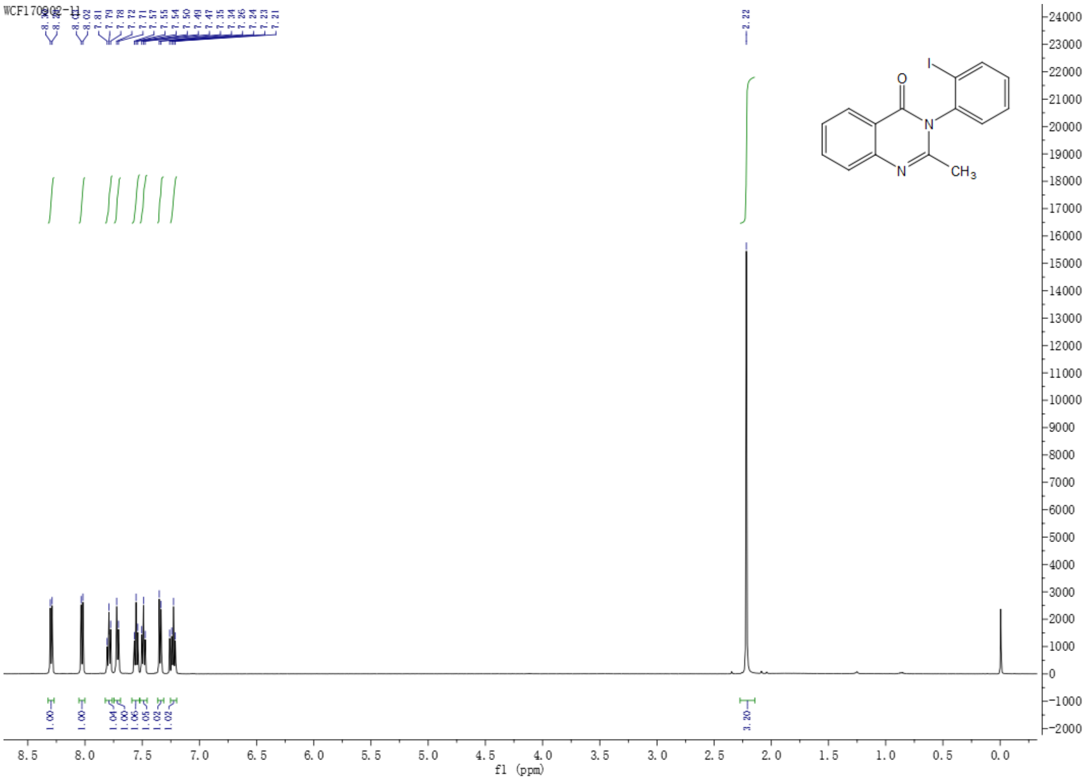

6.24. ^{1}H NMR (500 MHz, CDCl₃) of CPA 1a (0.015 mmol, 1.0 equiv.) and racemic guest 2m (0.01 mmol, 1.0 equiv.).

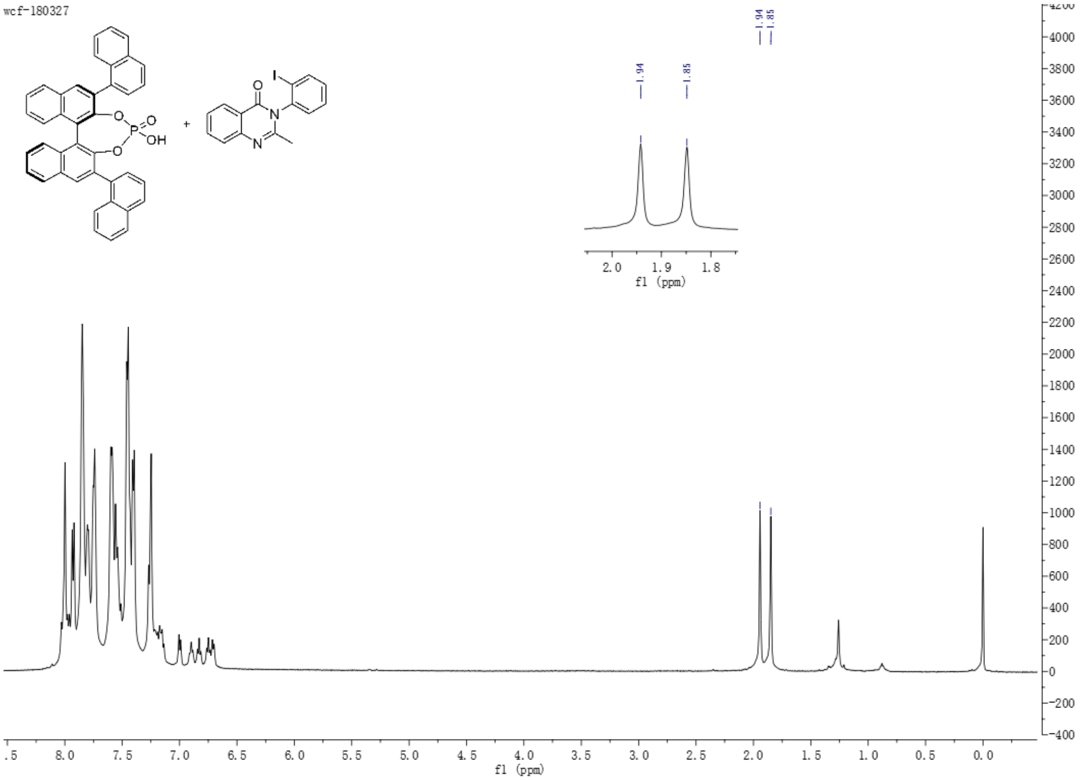

6.25. ^{1}H NMR (500 MHz, CDCl₃) of racemic afloqualone.

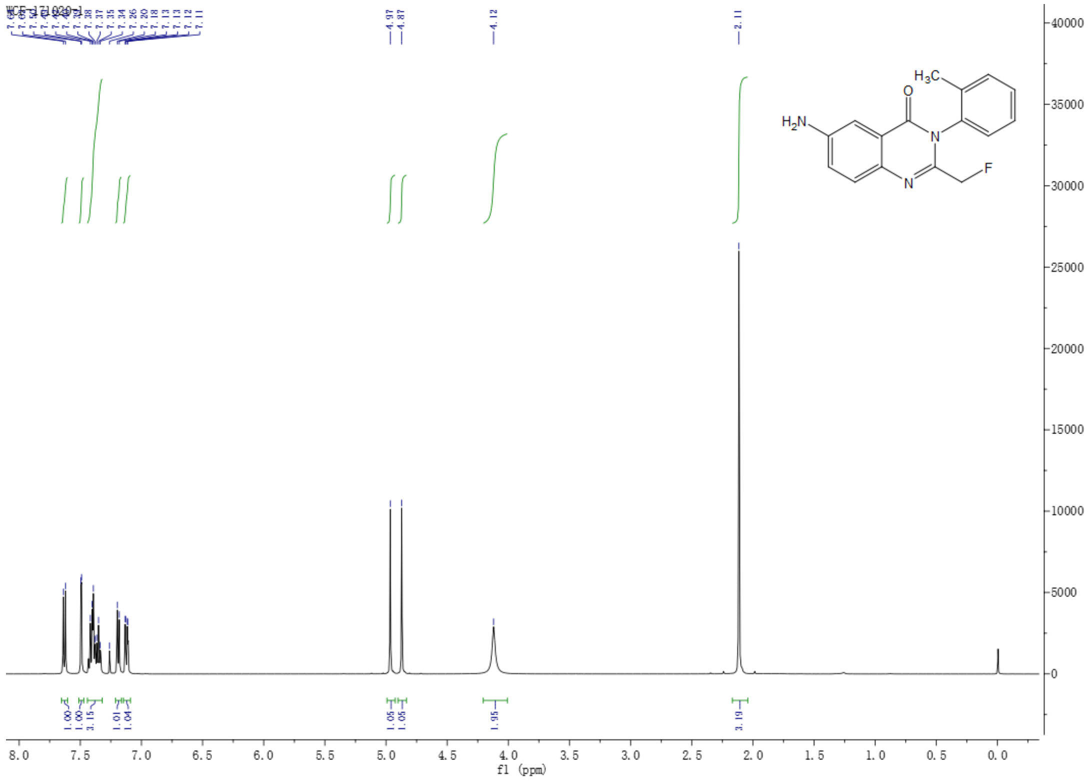

6.26. ^{1}H NMR (500 MHz, CDCl3) of CPA 1a and racemic afloqualone.

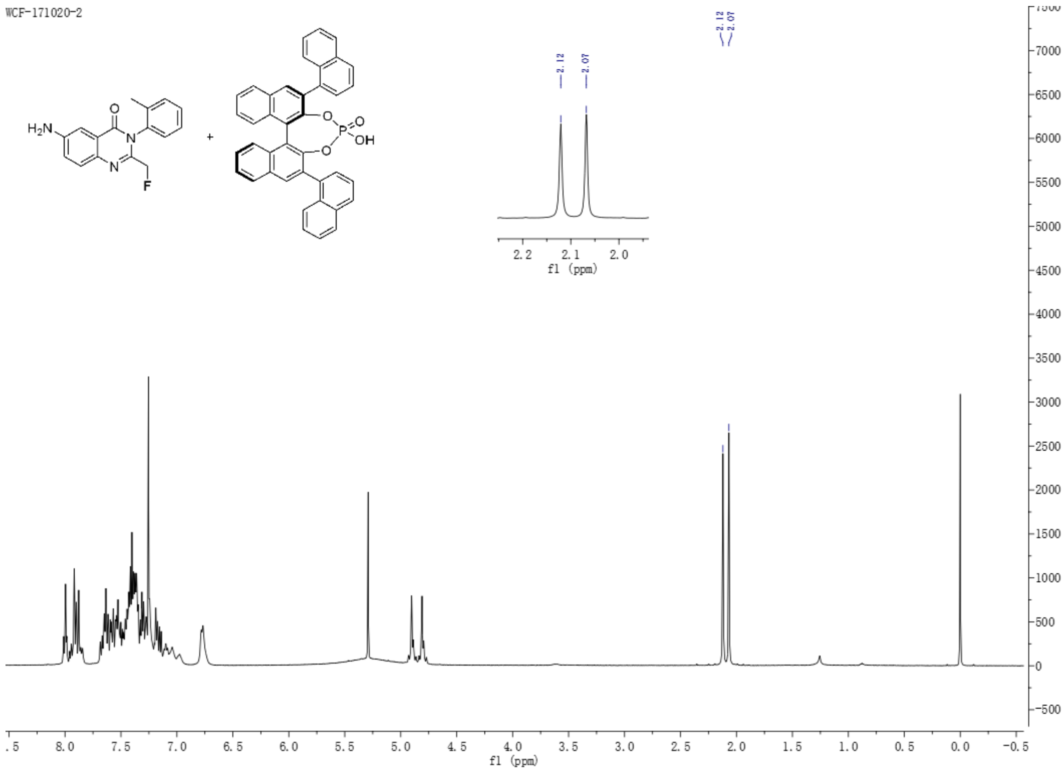

6.27. ^{1}H NMR (500 MHz, CDCl3) of racemic IC-87114.

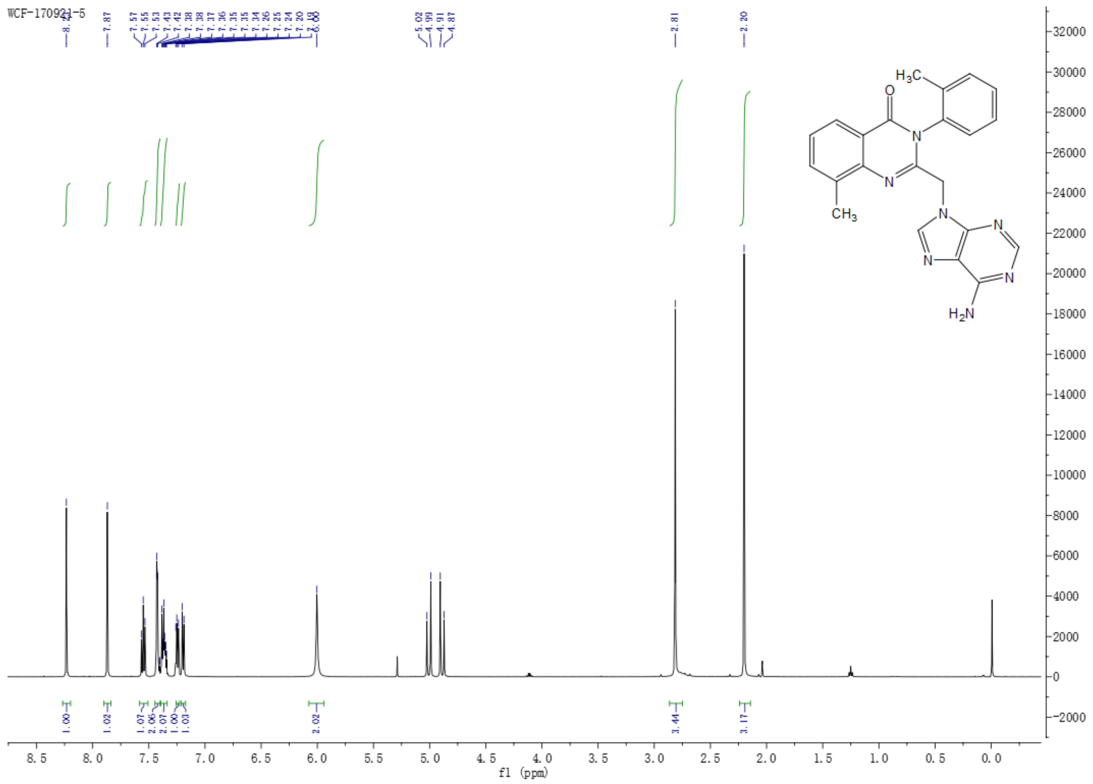

6.18. ^{13}C NMR (126 MHz, $CDCl_3$) of CPA 1a and racemic IC-87114.

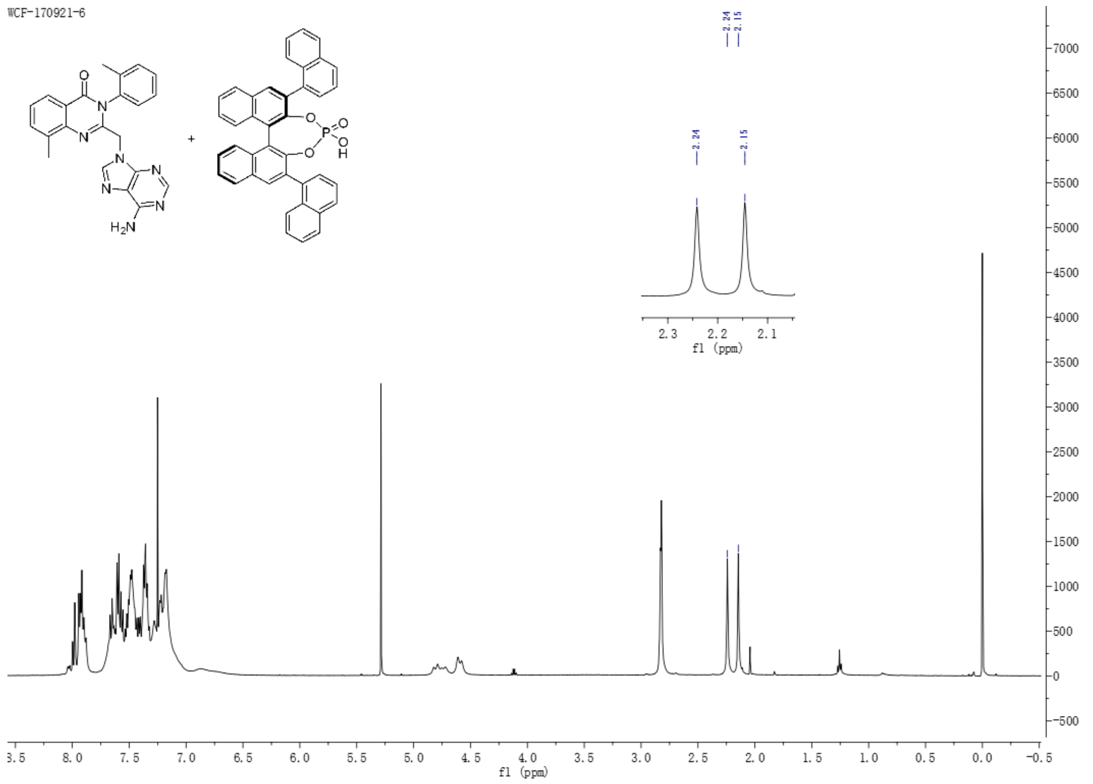

6.29. ^{13}C NMR (125 MHz, $CDCl_3$) of racemic 2e.

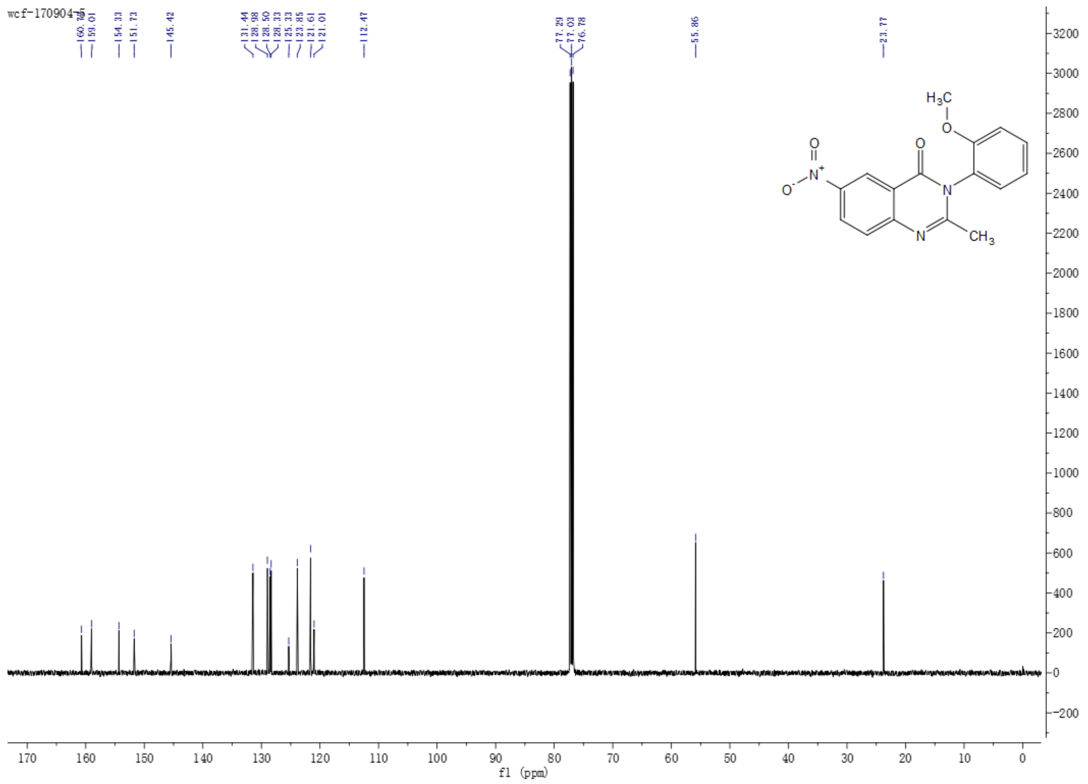

6.30. ^{13}C NMR (125 MHz, $CDCl_3$) of racemic 2c.

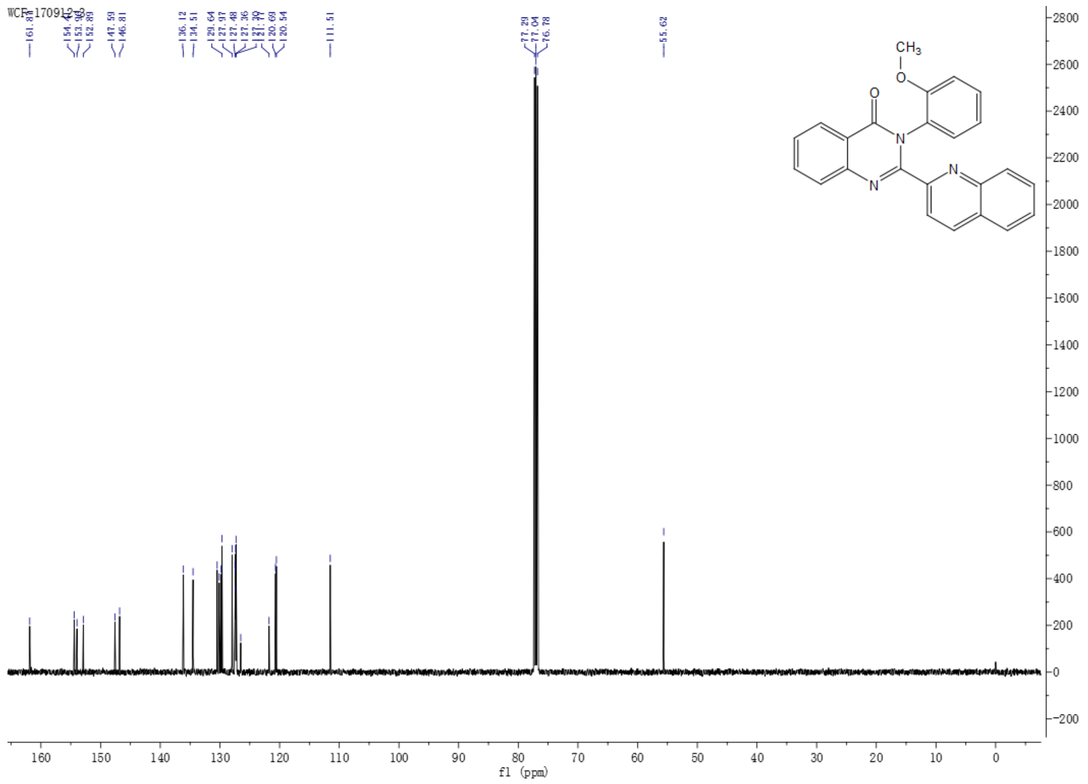

6.31. ^{13}C NMR (125 MHz, $CDCl_3$) of racemic 2g.

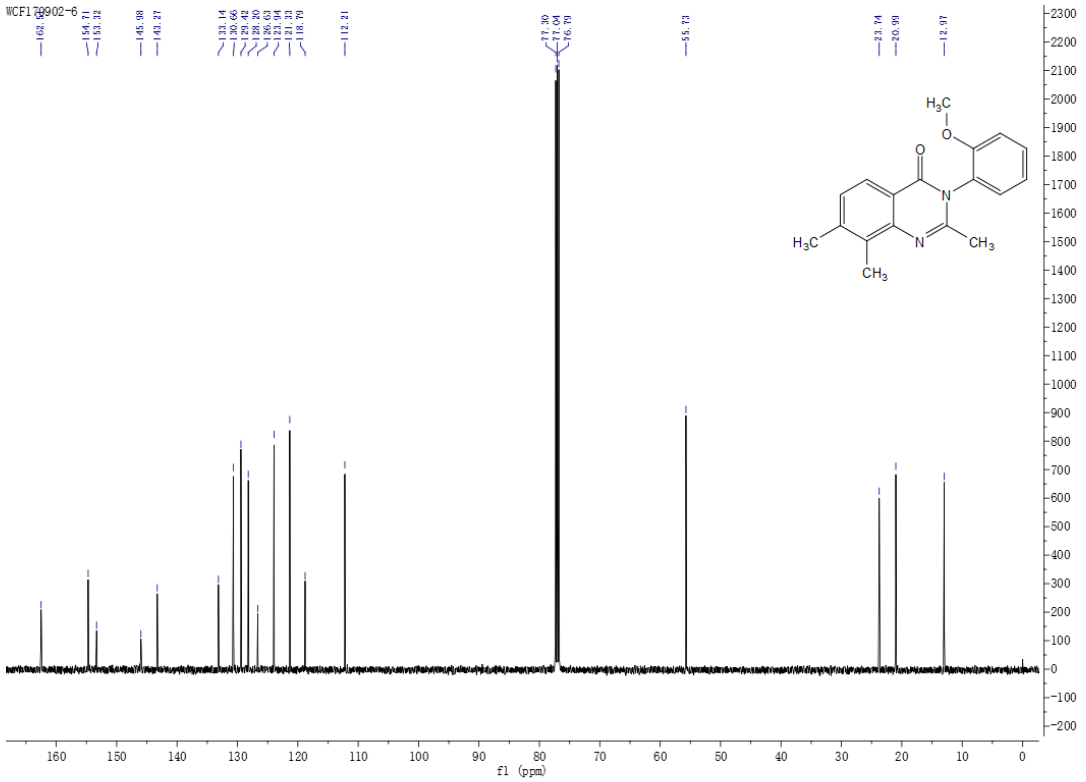

6.32. ^{13}C NMR (125 MHz, $CDCl_3$) of racemic 2i.

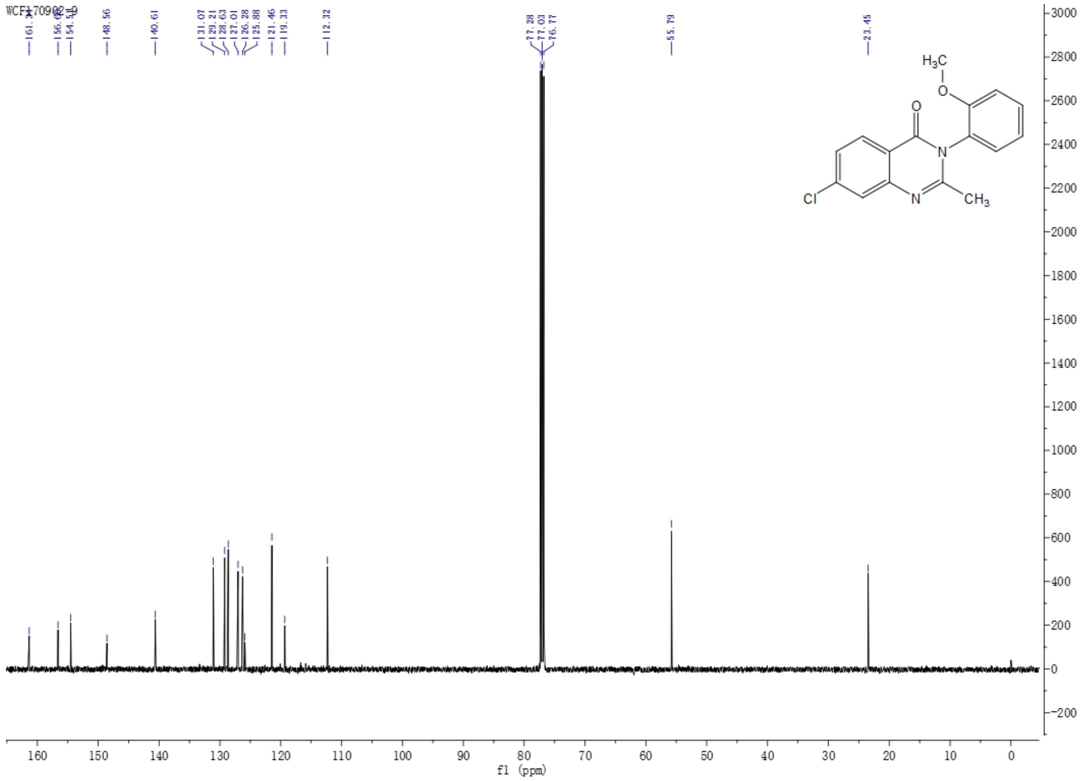

6.33. ^{13}C NMR (125 MHz, $CDCl_3$) of racemic 2k.

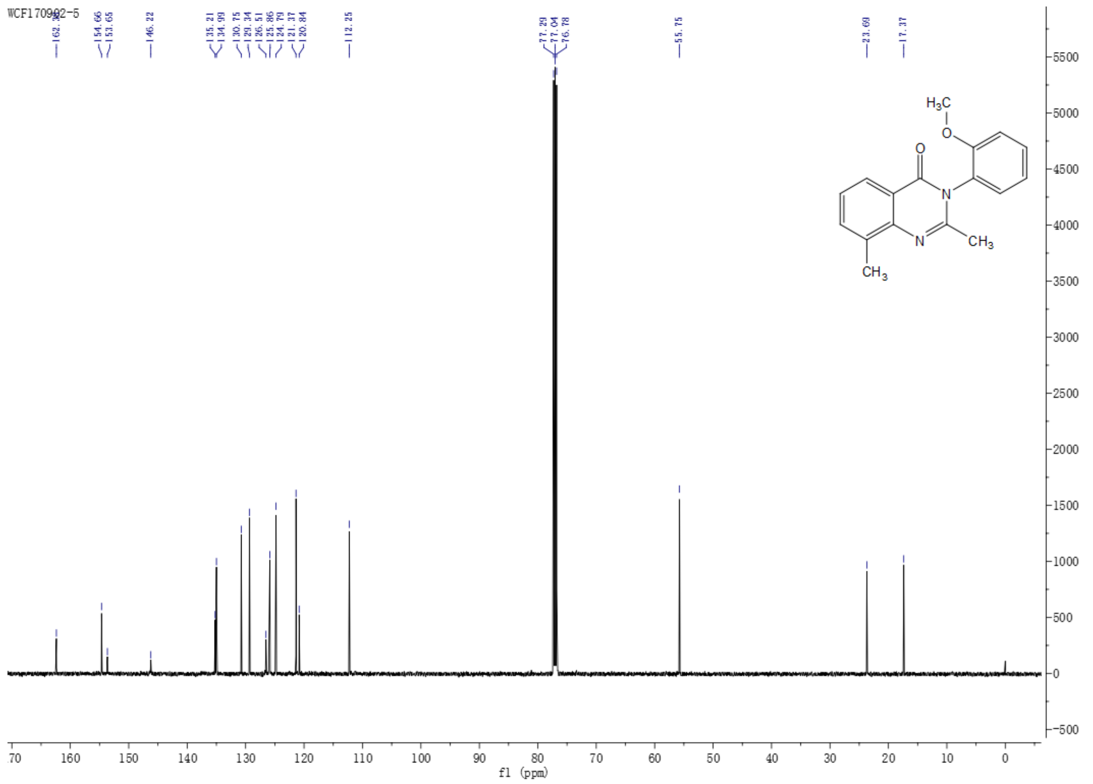

6.34. ^{13}C NMR (125 MHz, $CDCl_3$) of racemic 2l.

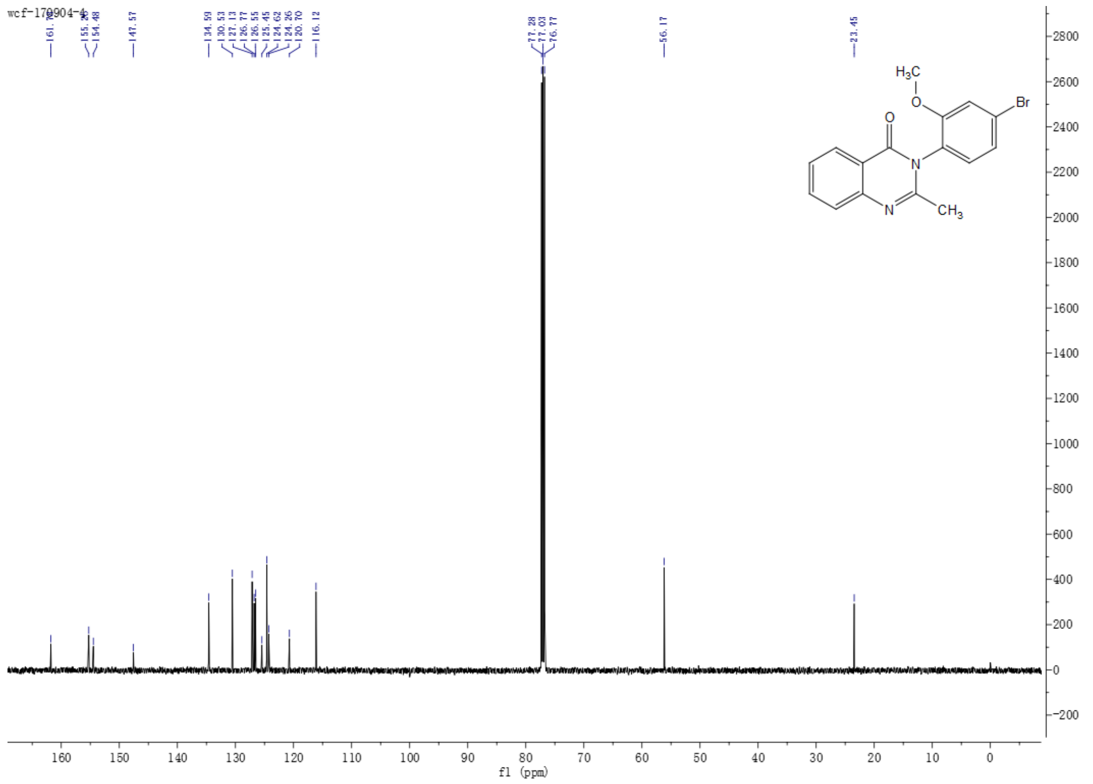

7. ^{1}H NMR spectra/HPLC data of 1a (0.01 mmol, 1.0 equiv.) and 2 (0.01 mmol, 1.0 equiv.) with different optical purities.

7.1. ^{1}H NMR (500 MHz, $CDCl_3$) of CPA 1a and guest 2a (Sample 1, ee 88.7%).

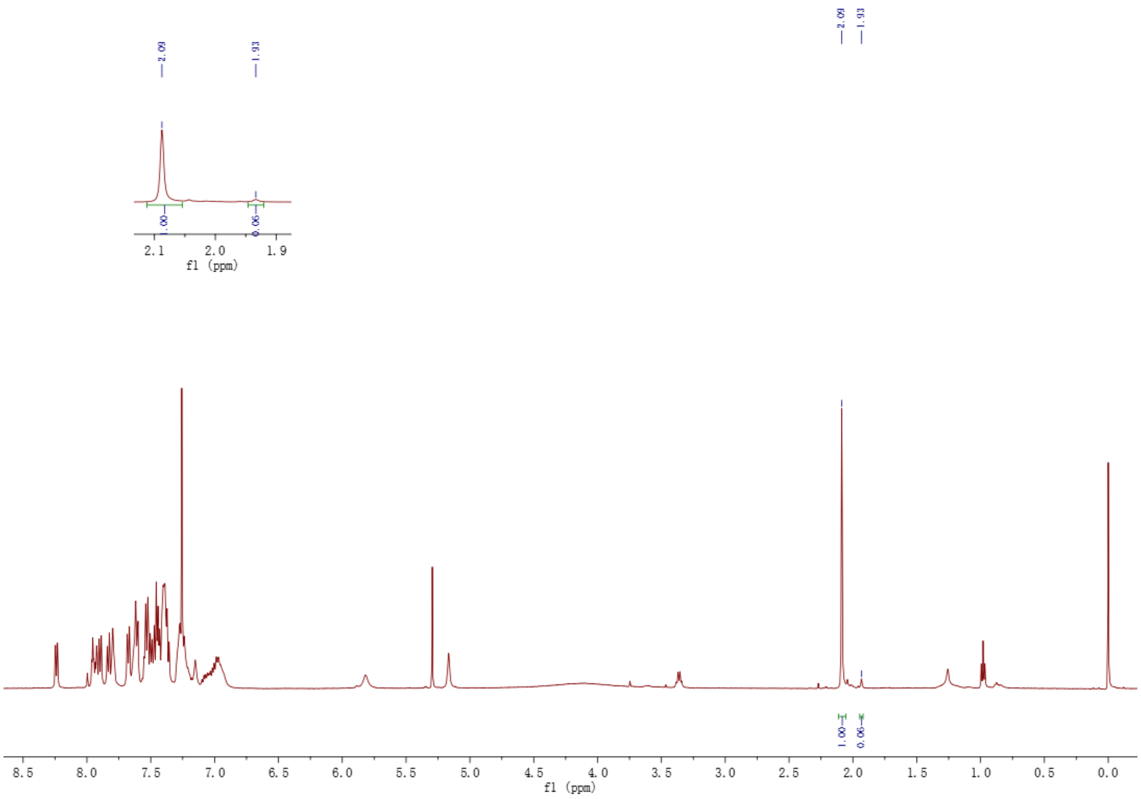

7.2. HPLC of guest 2a' (Sample 1, ee 89.7%).

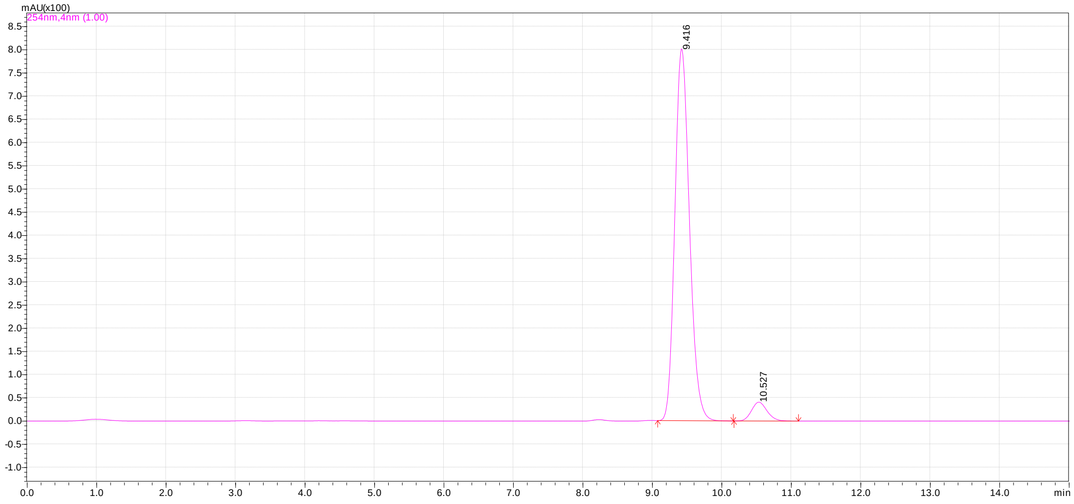

Peak	Ret. Time	Area	Height	Peak Start	Peak End	Area%
1	9.416	11734783	801261	9.077	10.165	94.8523
2	10.527	636859	40677	10.176	11.104	5.1477

7.3. ^{1}H NMR (500 MHz, $CDCl_3$) of CPA 1a and guest 2a (Sample 2, ee 81.8%).

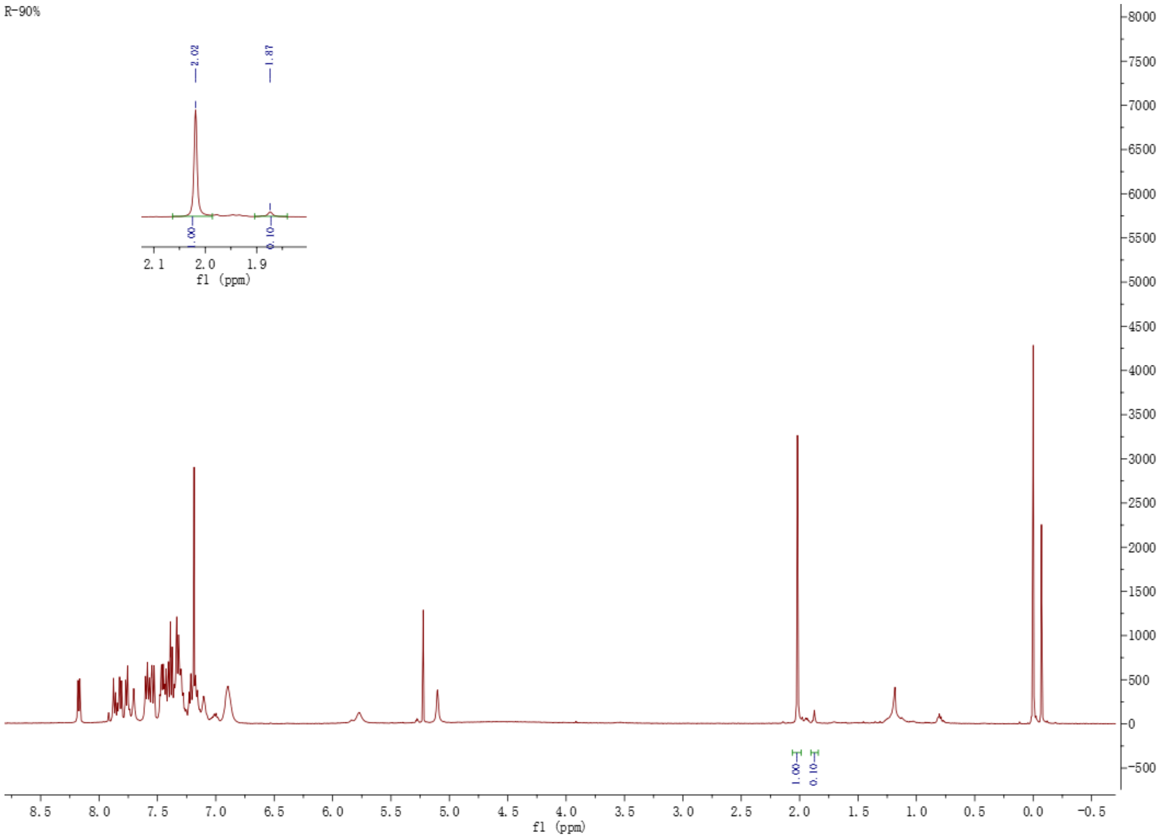

7.4. HPLC of guest 2a' (Sample 2, ee 82.1%).

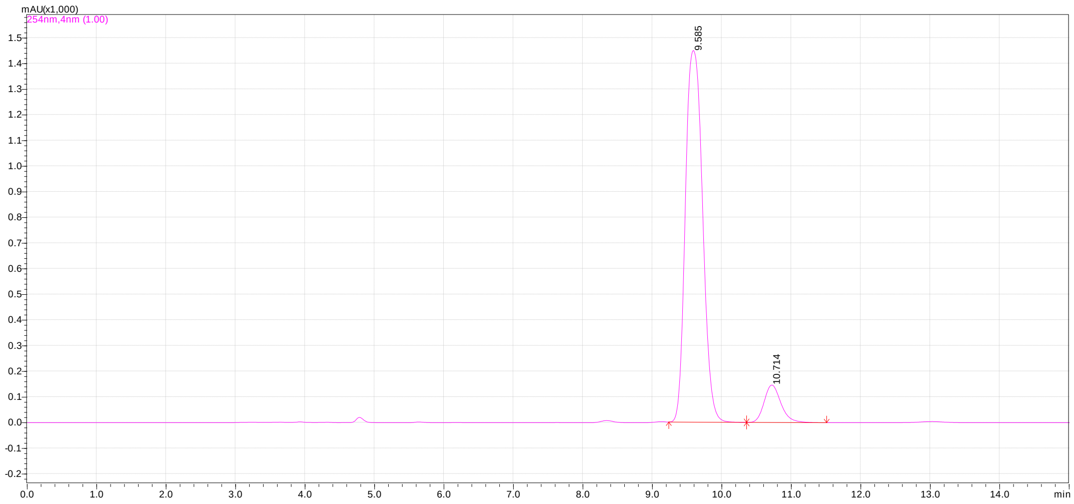

Peak	Ret. Time	Area	Height	Peak Start	Peak End	Area%
1	9.585	24945918	1449337	9.237	10.357	91.0520
2	10.714	2451514	146255	10.357	1.509	8.9480

7.5. ^{1}H NMR (500 MHz, $CDCl_3$) of CPA 1a and guest 2a (Sample 3, ee 61.3%).

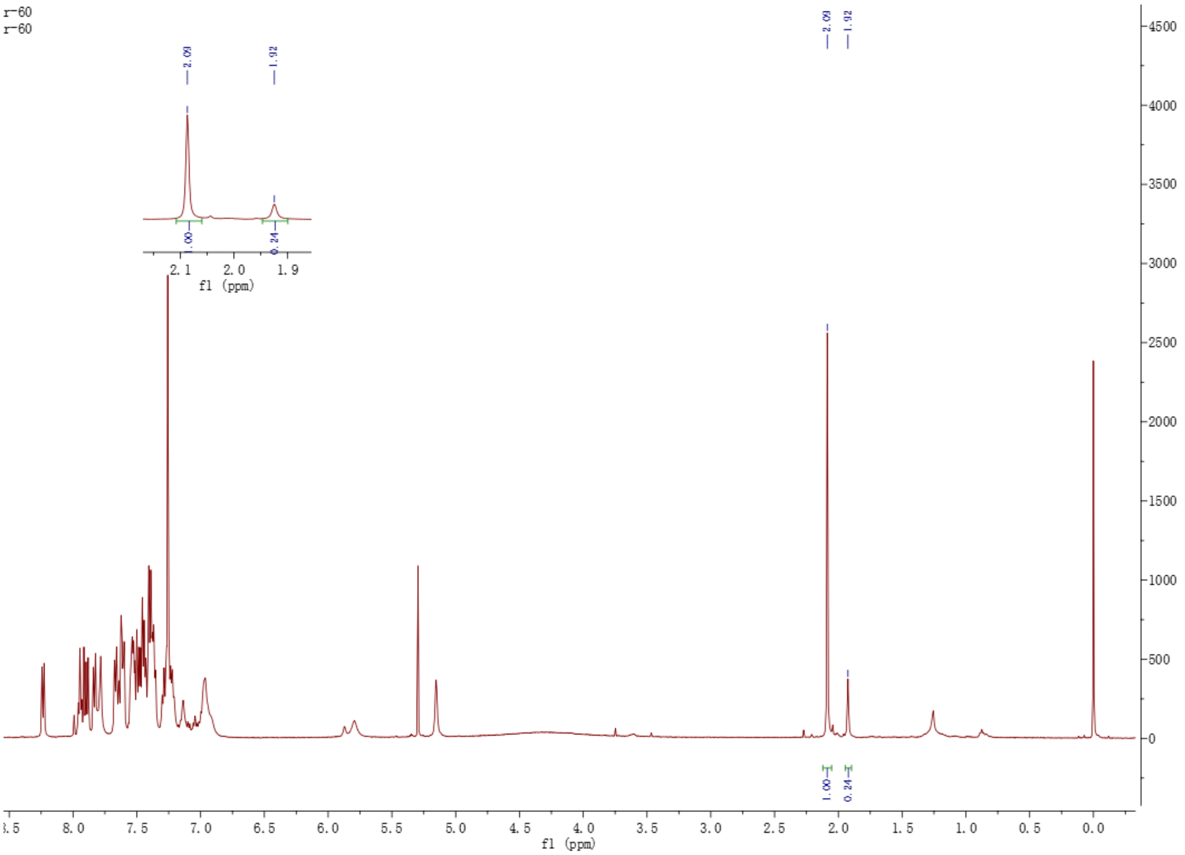

7.6. HPLC of guest 2a'(Sample 3, ee 60.6%).

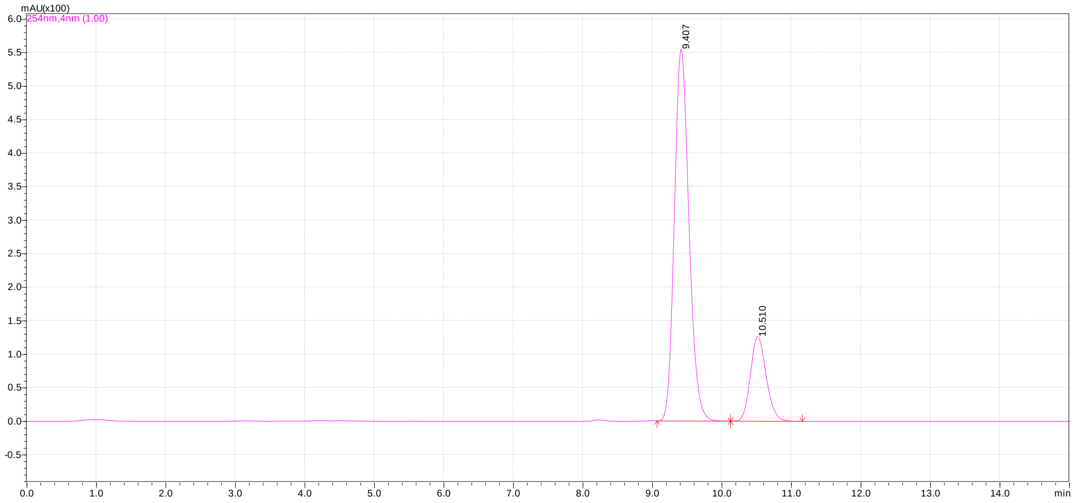

Peak	Ret. Time	Area	Height	Peak Start	Peak End	Area%
1	9.407	8113734	554175	9.067	10.123	80.2617
2	10.510	1995364	126431	10.123	11.157	19.7383

7.7. ^{1}H NMR (500 MHz, $CDCl_3$) of CPA 1a and guest 2a (Sample 4, ee 37.9%).

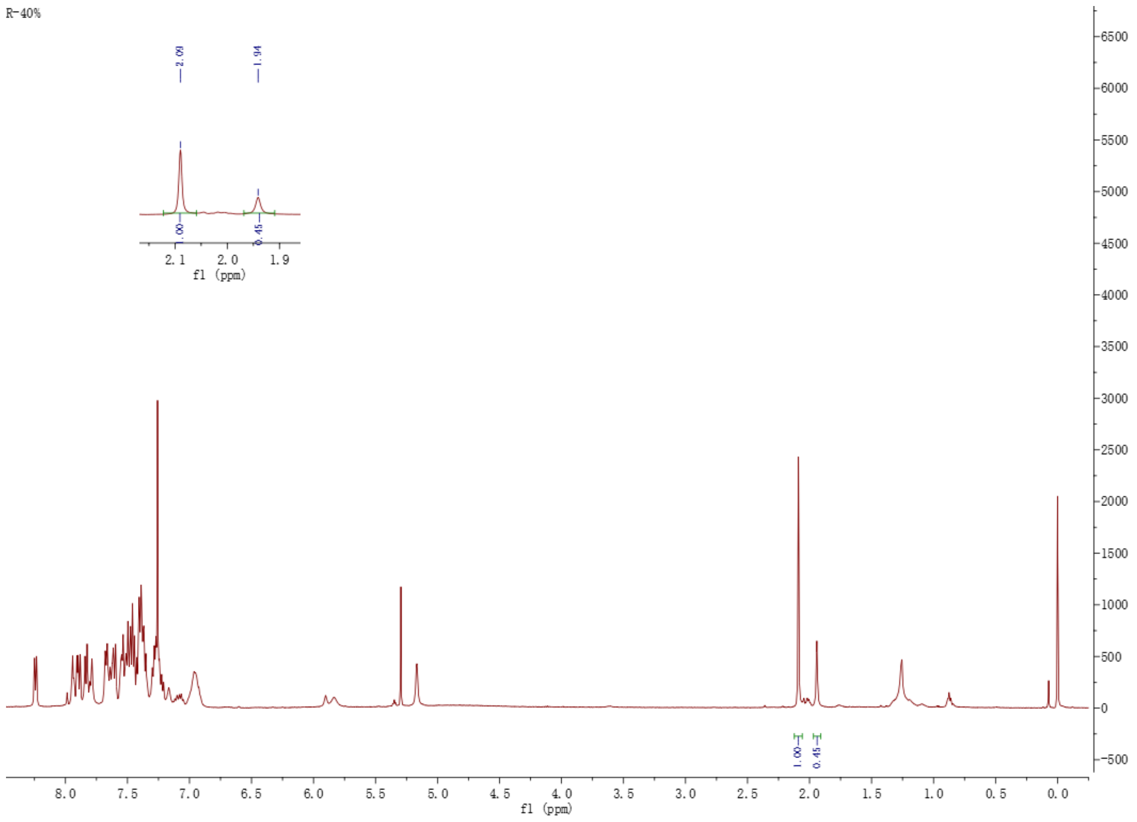

7.8. HPLC of guest 2a' (Sample 4, ee 39.2%).

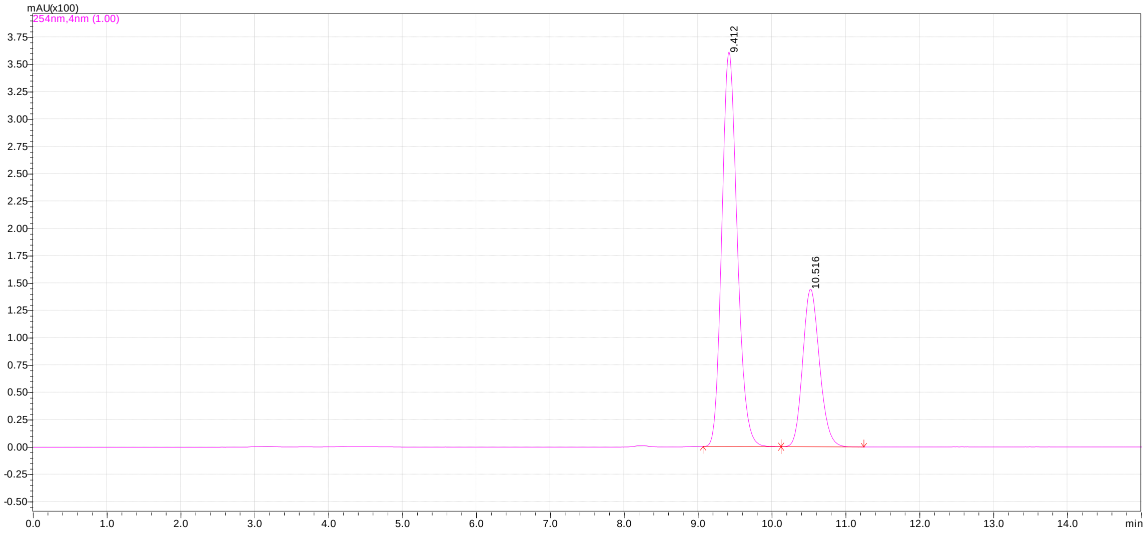

Peak	Ret. Time	Area	Height	Peak Start	Peak End	Area%
1	9.412	5237274	361063	9.067	10.123	69.6105
2	10.516	2286413	144198	10.123	11.243	30.3895

7.9. ^{1}H NMR (500 MHz, $CDCl_3$) of CPA 1a and guest 2a (Sample 5, ee 25.0%).

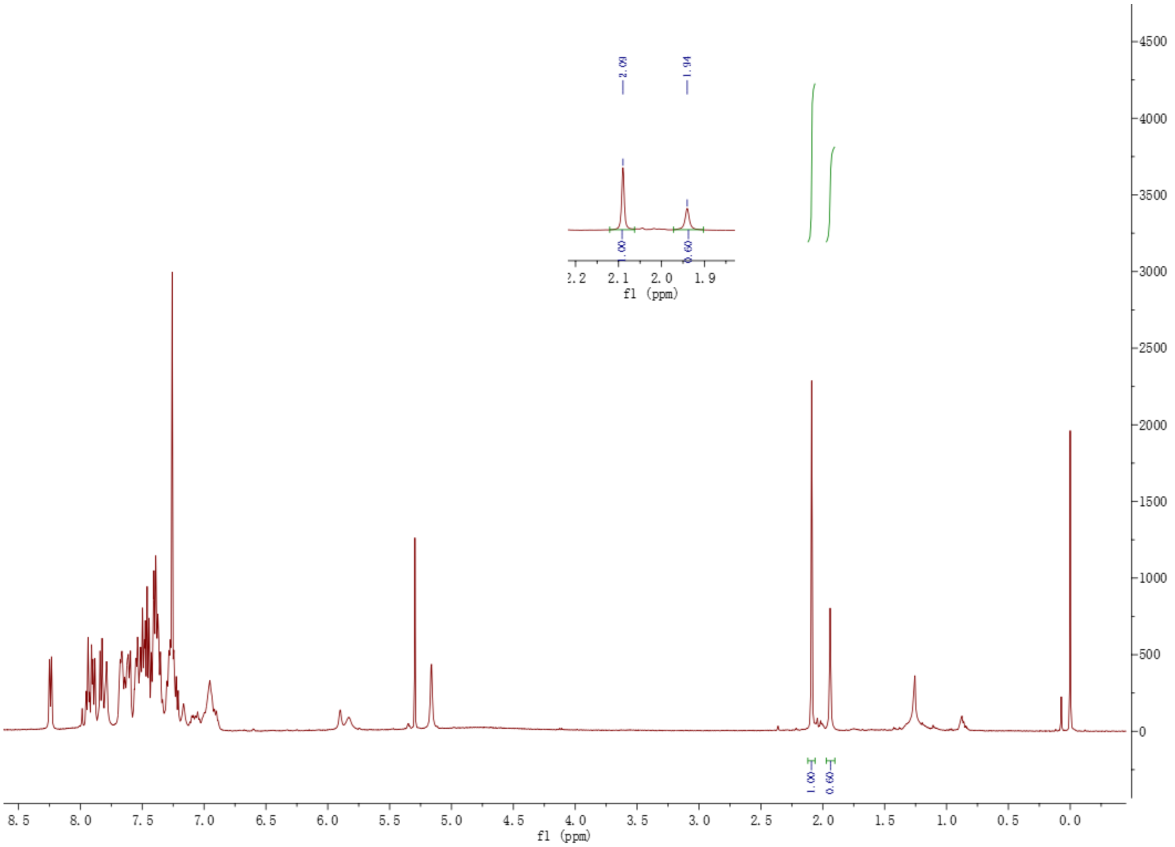

7.10. HPLC of guest 2a' (Sample 5, ee 24.4%).

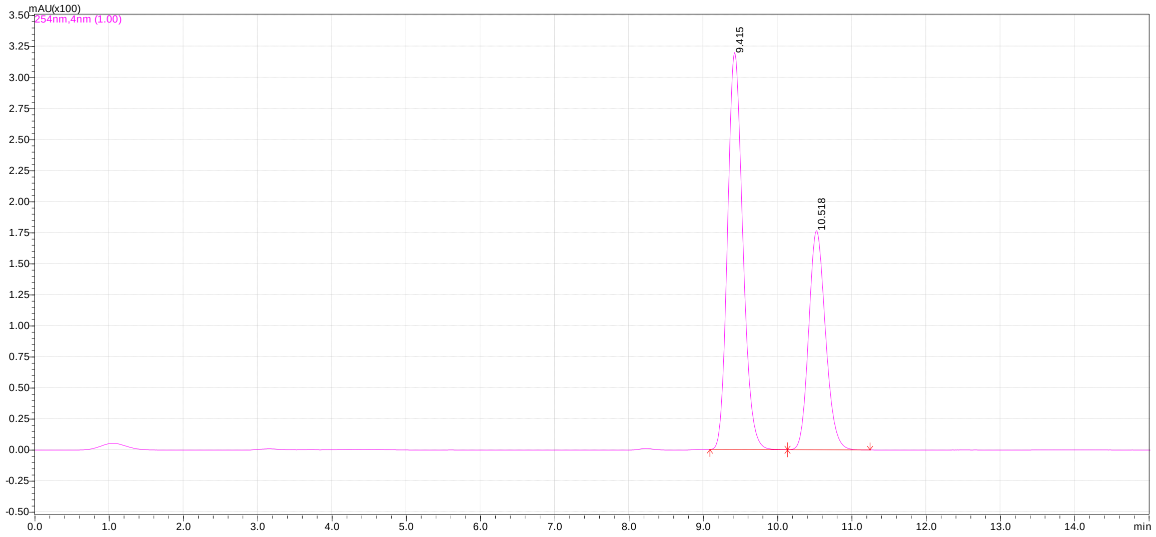

Peak	Ret. Time	Area	Height	Peak Start	Peak End	Area%
1	9.415	4584414	319843	9.088	10.133	62.2454
2	10.518	2780648	176391	10.133	11.243	37.7546

7.11.^{1}H NMR (500 MHz, $CDCl_3$) of CPA 1a and guest 2a (Sample 6, ee -5.6%).

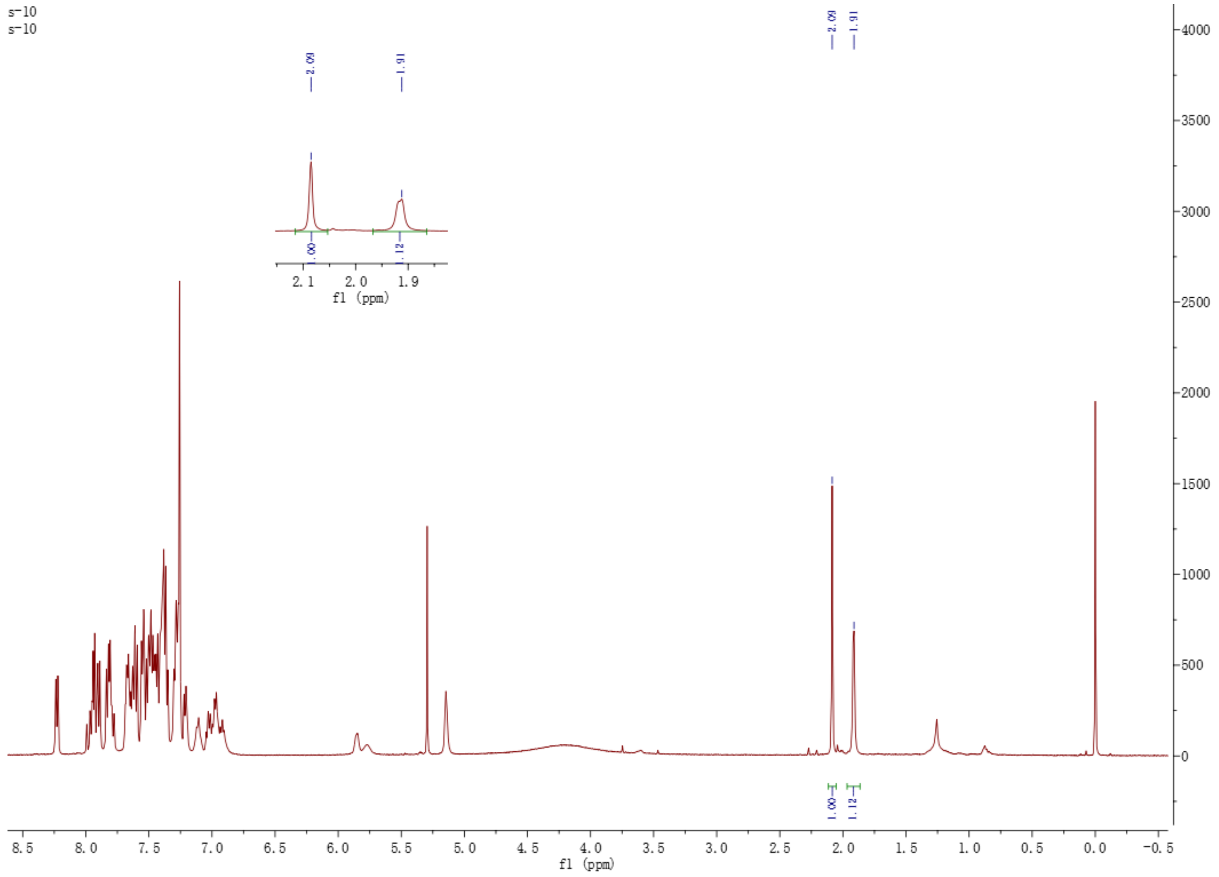

7.12. HPLC of guest 2a'(Sample 6, ee -6.0%)

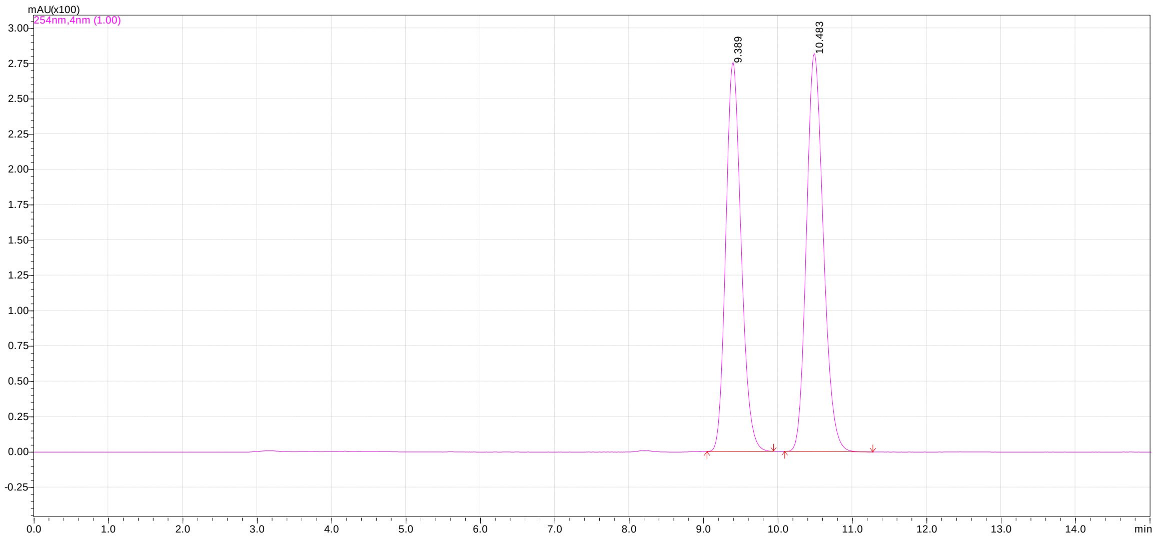

Peak	Ret. Time	Area	Height	Peak Start	Peak End	Area%
1	9.389	3956506	275337	9.045	9.941	46.9893
2	10.483	4463509	281690	10.091	11.275	53.0307

7.13. ^{1}H NMR (500 MHz, $CDCl_3$) of CPA 1a and guest 2a (Sample 7, ee -18.0%).

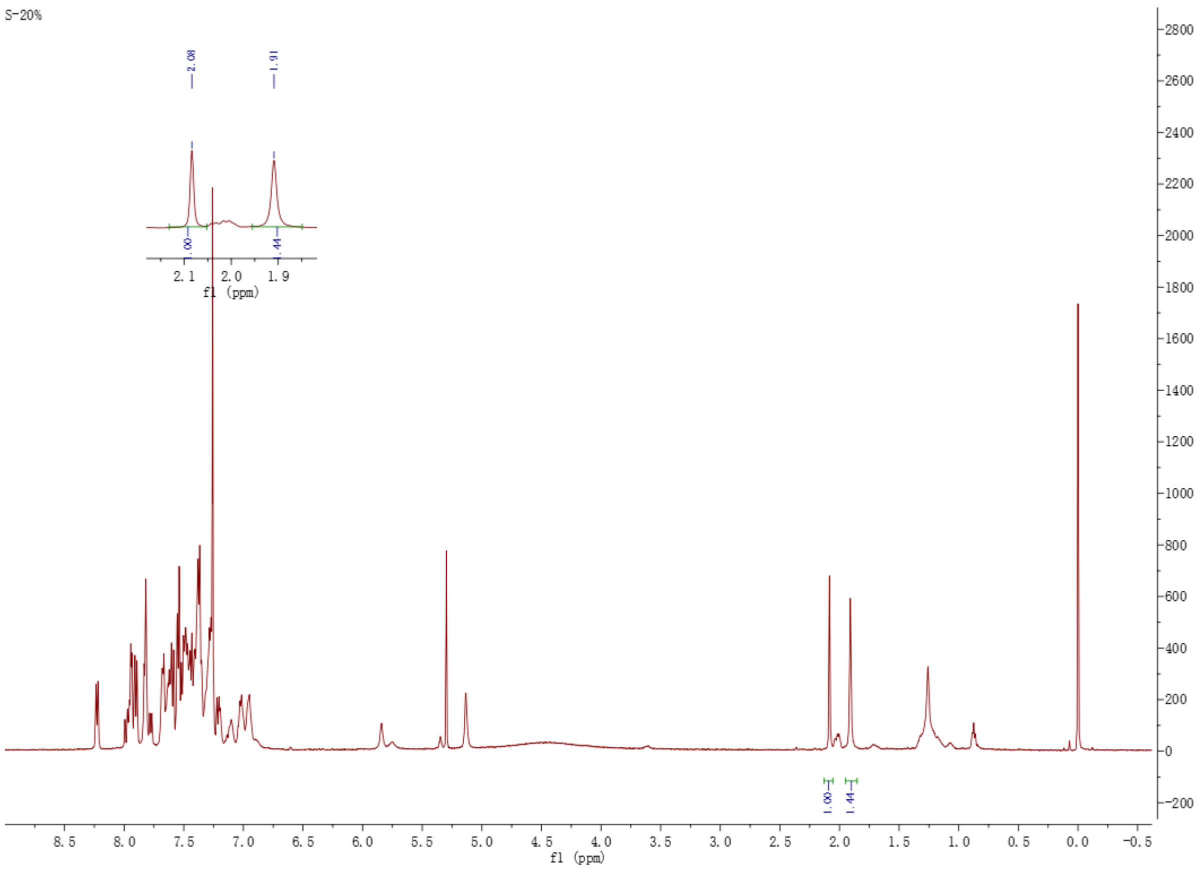

7.14. HPLC of guest 2a' (Sample 7, ee -16.6%).

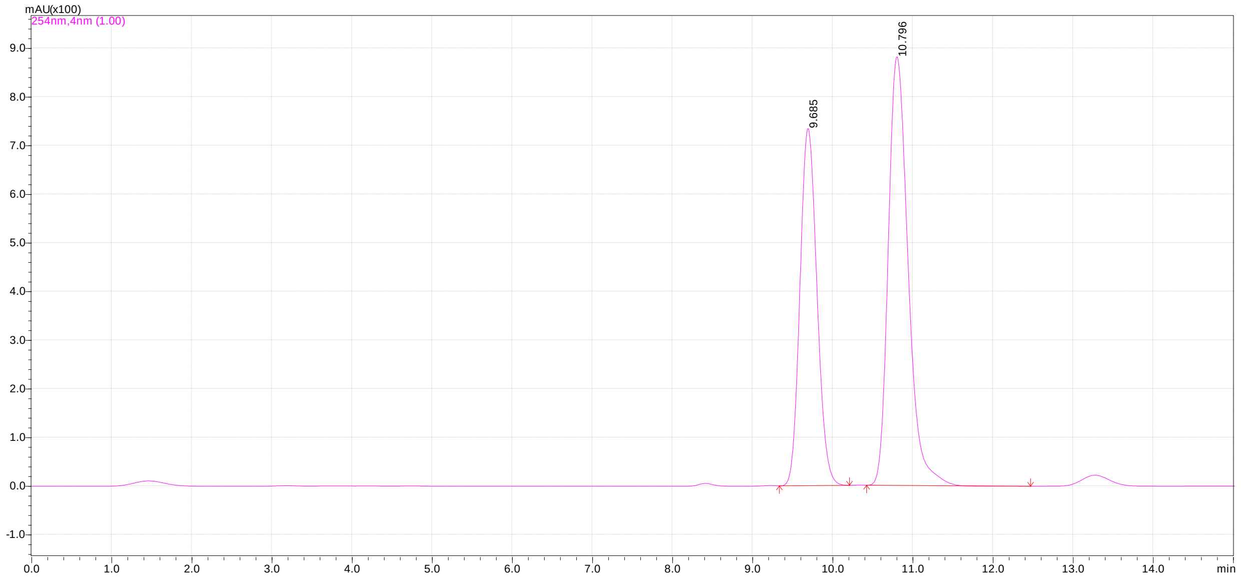

Peak	Ret. Time	Area	Height	Peak Start	Peak End	Area%
1	9.685	10830201	733612	9.333	10.208	41.6902
2	10.796	15147612	880733	10.421	12.469	58.3098

7.15. ^{1}H NMR (500 MHz, $CDCl_3$) of CPA 1a and guest 2a (Sample 8, ee -37.1%).

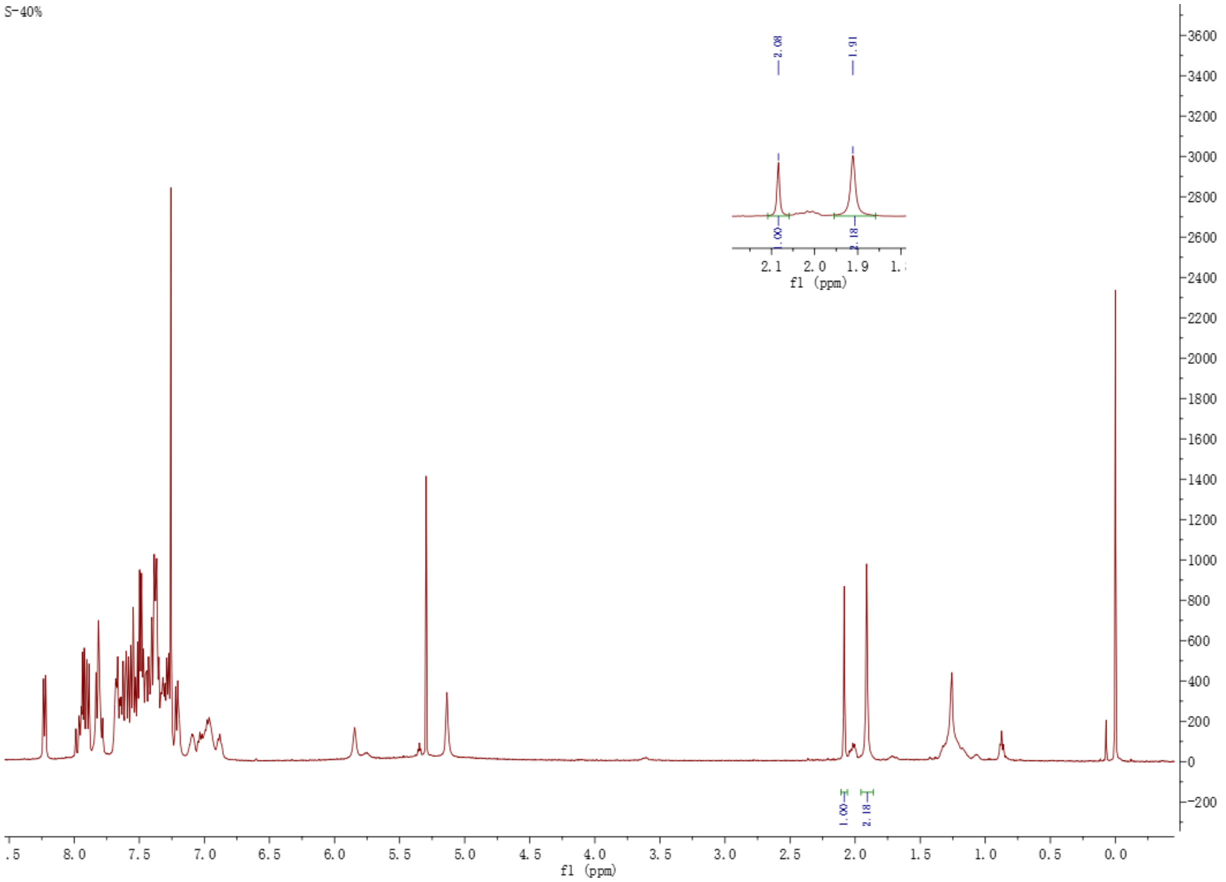

7.16. HPLC of guest 2a' (Sample 8, ee -37.8%).

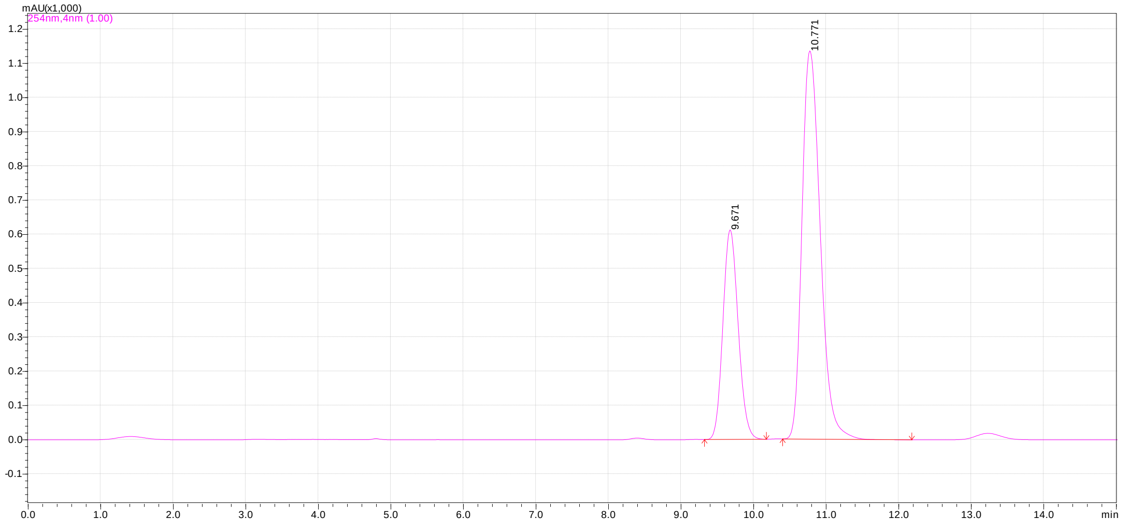

Peak	Ret. Time	Area	Height	Peak Start	Peak End	Area%
1	9.671	8971616	612573	9.323	10.176	31.0917
2	10.771	19883755	1134501	10.400	12.181	68.9083

7.17. ^{1}H NMR (500 MHz, $CDCl_3$) of CPA 1a and guest 2a (Sample 9, ee -59.8%).

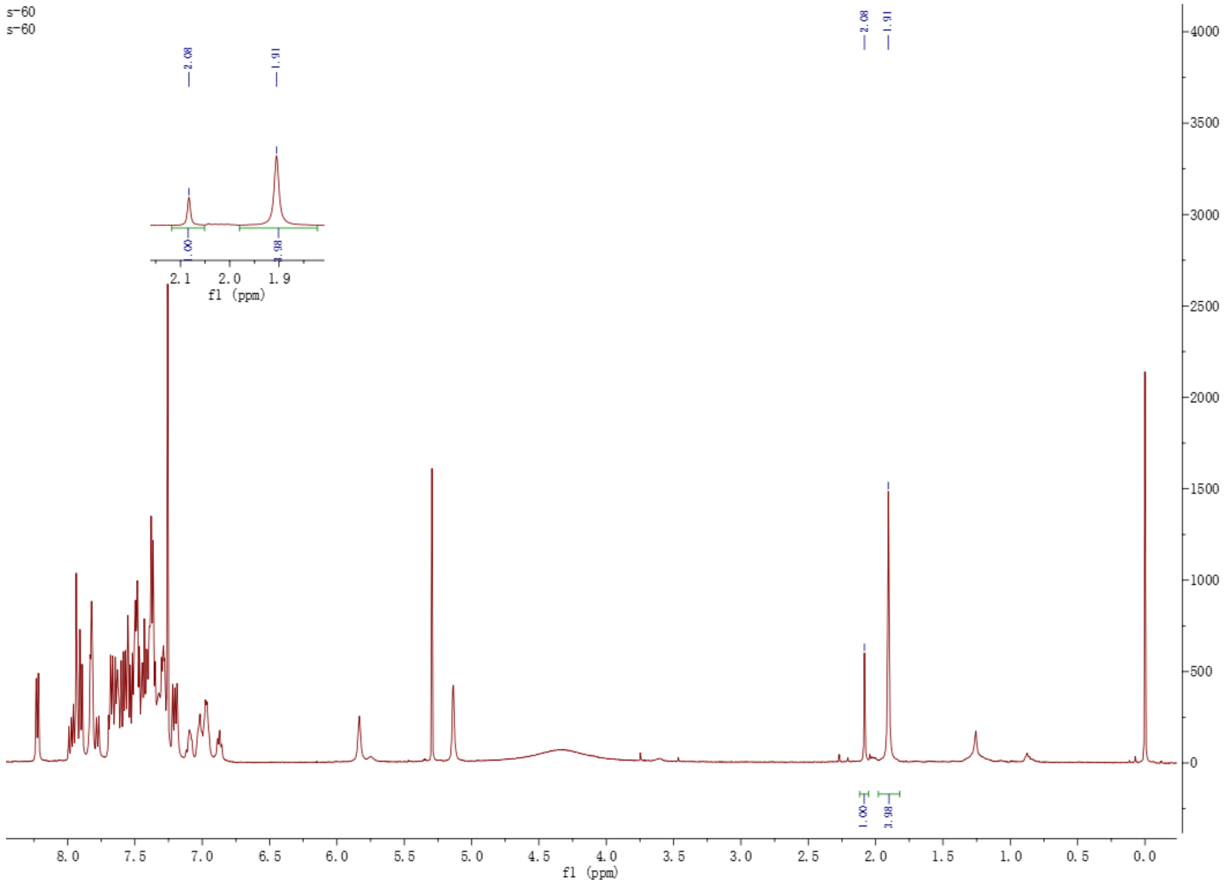

7.18. HPLC of guest 2a' (Sample 9, ee -60.8%).

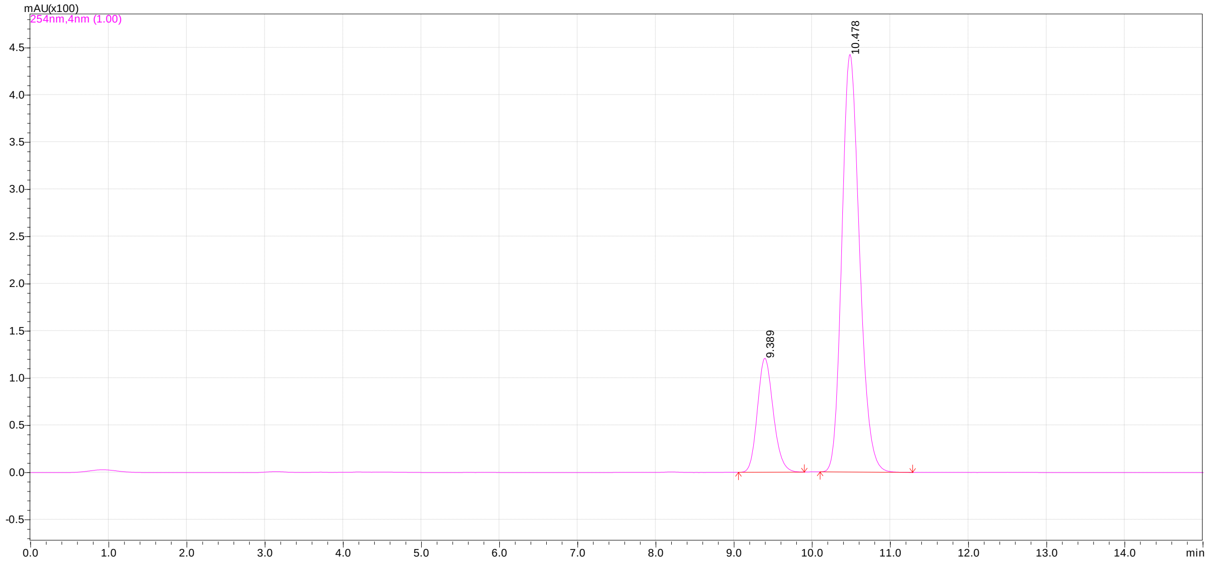

Peak	Ret. Time	Area	Height	Peak Start	Peak End	Area%
1	9.389	1701886	120710	9.056	9.899	19.6403
2	10.478	6963390	442574	10.101	11.285	80.3597

7.19. ^{1}H NMR (500 MHz, $CDCl_3$) of CPA 1a and guest 2a (Sample 10, ee -77.9%).

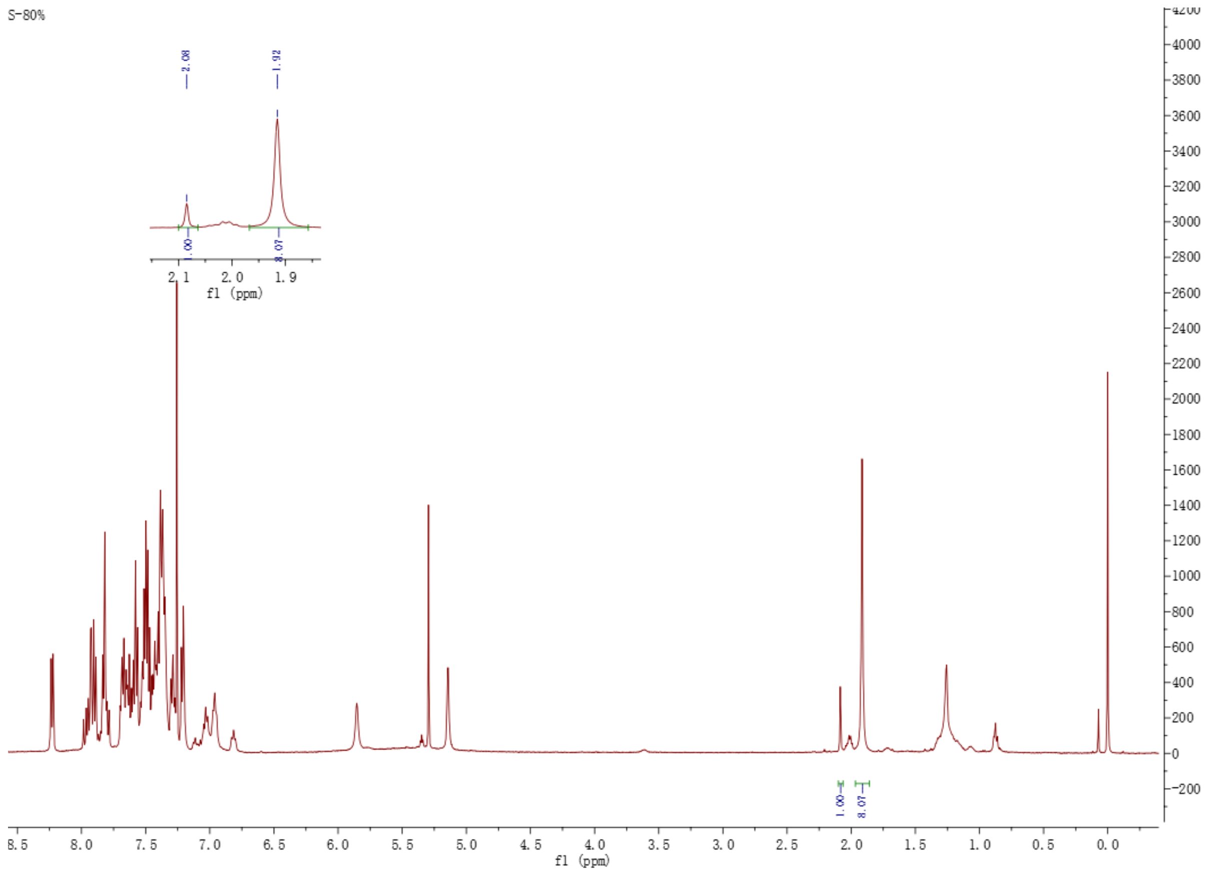

7.20. HPLC of guest 2a' (Sample 10, ee -78.6%).

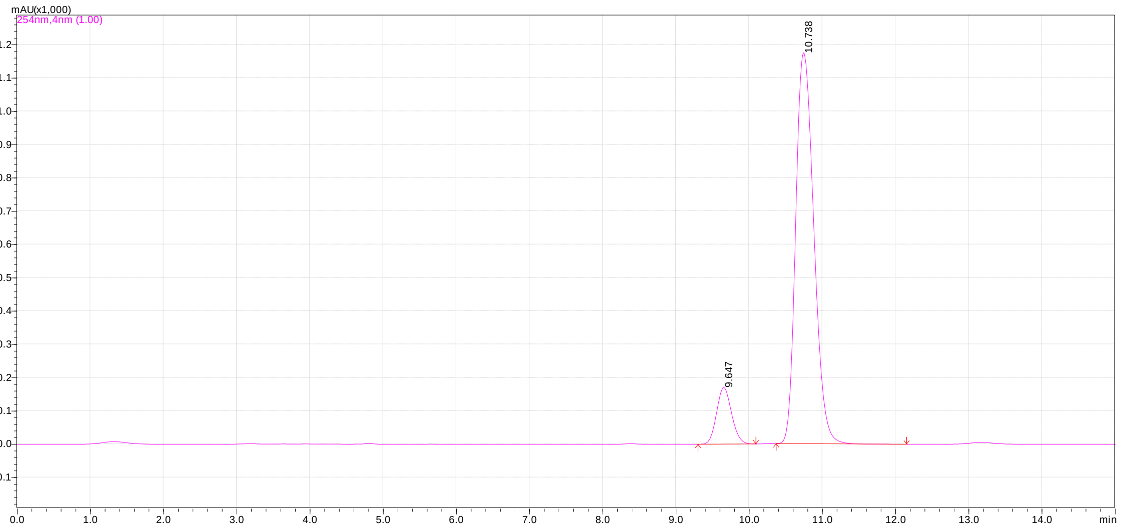

Peak	Ret. Time	Area	Height	Peak Start	Peak End	Area%
1	9.647	2424371	169878	9.301	10.091	10.7309
2	10.738	20168105	1174081	10.368	12.149	89.2691

7.21. ^{1}H NMR (500 MHz, $CDCl_3$) of CPA 1a and guest 2a (Sample 10, ee -87.1%).

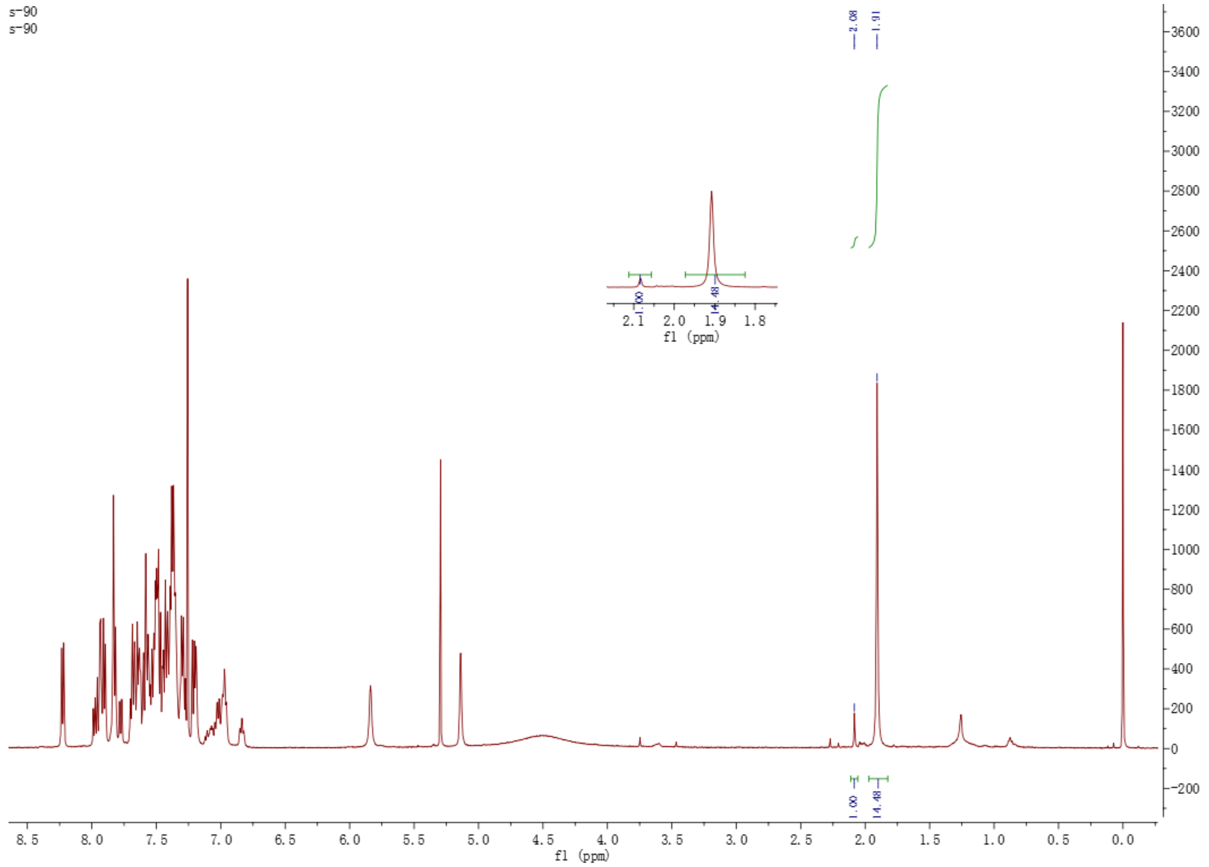

7.22. HPLC of guest 2a' (Sample 10, ee -87.2%).

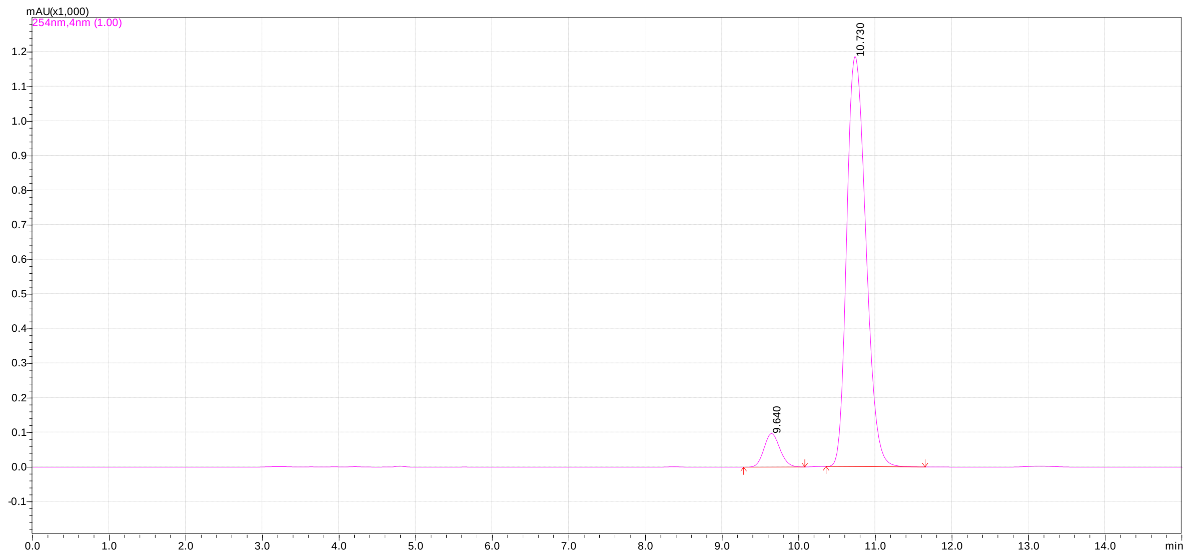

Peak	Ret. Time	Area	Height	Peak Start	Peak End	Area%
1	9.640	1401242	96853	9.280	10.080	6.3669
2	10.738	20607052	1184646	10.357	11.648	93.6331
